# Non-steroidal anti-inflammatory drugs and bone healing in animal models—a systematic review and meta-analysis

**DOI:** 10.1186/s13643-021-01690-w

**Published:** 2021-07-08

**Authors:** Haider Al-Waeli, Ana Paula Reboucas, Alaa Mansour, Martin Morris, Faleh Tamimi, Belinda Nicolau

**Affiliations:** 1 Faculty of Dentistry, Dalhousie University, 5981 University Ave, Halifax, Nova Scotia B3H 4R2 Canada; 2 Faculty of Dentistry, Federal University of Minas Gerais, Minas Gerais, Brazil; 3 School of Dental Medicine, University at Buffalo, Buffalo, NY 14214 USA; 4 Schulich Library, McGill University, 2001 Avenue McGill College Suite 500, Montréal, QC H3A 1G1 Canada; 5 College of Dental Medicine, Qatar University, University Street, Doha, Qatar; 6 Faculty of Dentistry, McGill University, 2001 Avenue McGill College Suite 500, Montréal, QC H3A 1G1 Canada

**Keywords:** Fracture, NSAID, Cyclooxygenase, Prostaglandin, Bone, Systematic review and meta-analysis

## Abstract

**Background:**

Non-steroidal anti-inflammatory drugs (NSAID) have excellent anti-inflammatory and analgesic properties and are extensively used to treat post-traumatic or surgical musculoskeletal pain. Although an extensive literature exists on the administration of NSAID on animal bone healing, no systematic review and meta-analysis of animal studies that investigate the effect of NSAID administration on bone fracture healing. Objective of this study is to conduct a systematic review and meta-analysis to estimate the effect of NSAIDs administration on bone healing biomechanical and histomorphometric measurements in different animal models after bone fracture surgery.

**Methods:**

We performed a systematic review and meta-analysis of animal studies to estimate the effect of NSAID administration after bone fracture on healing outcomes. We searched eight databases without limiting the search to starting date up to 1 February 2021 for articles on fractured bone healing in animal models in which NSAID were administered.

**Results:**

Out of 6732 articles screened, 47 were included and 3 common bone healing outcomes were analysed: biomechanical properties (maximum force to break, stiffness, and work-to-failure), micro-computed tomography (μ-CT), and histomorphometric measurements. The studies were generally of low-quality scores because crucial information, especially concerning randomization, blinding, and allocation concealment, was poorly reported. Our results show that the negative effects of NSAID after bone fracture on certain biomechanical properties of the healing bones was not statistically significant in mice compared with other animals, in females compared with males, and in younger compared with older animals.

**Conclusion:**

The findings demonstrated that NSAIDs administration decreased the biomechanical properties of healing bones after fracture surgery in comparison to the control group. Moreover, different effect on certain outcomes was detected among different sites, sex of the animals, and the time of assessment.

**Trial registration:**

Protocol published and registered in SYstematic Review Center for Laboratory animal Experimentation (SYRCLE) in 2017, https://www.radboudumc.nl/getmedia/757ec408-7a9e-4635-8233-ae951effea54/Non-Steroidal-Anti-inflammatory-Drugs-and-bone-healing-in-animal-Models-Systematic-Review-and-Meta-Analysis.aspx

**Supplementary Information:**

The online version contains supplementary material available at 10.1186/s13643-021-01690-w.

## Background

Non-steroidal anti-inflammatory drugs (NSAID) have been extensively used to treat post-traumatic and surgical musculoskeletal pain because of the excellent anti-inflammatory and analgesic properties of cyclooxygenase (COX) inhibitors [1–5]. However, clinical and biological data suggest that COX inhibition has a negative impact on bone tissue repair. Results from several animal trials have suggested that decreased bone healing, including biomechanical and histomorphometric properties, was associated with NSAID administration after bone fracture [6–12]. While few studies have investigated the effect of NSAIDs on bone healing in humans, their results are contradictory, possibly reflecting methodological limitations [13–16]. Indeed, authors have argued for well-designed, large, multicentric randomized controlled trials with appropriately defined endpoints [17]. To the best of our knowledge, there are no clinical studies to accurately determine the effect of NSAID on bone healing.

Several animal models including different animal strains, sex, and fracture techniques have been used to test the effect of types and administration durations of NSAID on bone healing outcomes [10, 18–24]. The assessments of bone healing in these studies include integrated multilevel measurements at the organ (e.g., biomechanical to tissue levels using micro-computed tomography (μ-CT) analysis), cellular (e.g., histology, histomorphometric analysis), and gene (e.g., mRNA microarray to explain the associated healing pathway) levels [25, 26]. Overall, the techniques used to assess bone healing can be divided into four main categories: imaging analyses, biomechanical tests, detection of serologic markers, and clinical examinations [25]. Among these categories, the biomechanical confirmed by histomorphometric analysis is the best way to determine the success of fracture healing [27, 28]. These tests are widely used in animal studies at different time points of fracture healing, which are selected according to the biological, physiological, and pathological changes of the fractured bone [28–31].

Although an extensive literature exists on the administration of NSAID on animal bone healing, no systematic review and meta-analysis have yet been conducted on the subject. Such work is important because it can identify the key histomorphometric and biomechanics characteristics during the healing process as well as it provides information on different factors that may affect the healing process after NSAID administration (e.g., NSAID type, animal type and strain, sex of the animal, fracture type, time of assessment). This information may help to design future experimental trials and facilitate knowledge translation. Therefore, we performed a systematic review and meta-analysis of all identified and available animal studies on the effect of NSAID administration after bone fracture on healing outcomes.

Specifically, our objectives were to estimate the extent to which the effect of NSAID administration after bone fracture using animal models: (i) results in less favorable bone-biomechanical and morphometric healing measurements; and (ii) differs by animal type, age and sex, type of NSAID, and length of follow-up.

## Methods

The systematic review methodology was specified and documented in advance using SYstematic Review Center for Laboratory animal Experimentation (SYRCLE)’s protocol template for animal studies and registered in the SYRCLE database (Supplementary material Table S1) [32, 33]. In our initial protocol, we had proposed to carry out a sensitivity analysis to assess whether the methodological quality of the studies included in the meta-analysis greatly influences the findings of the review. However, most of the studies in our systematic reviewed received low-quality scores; we therefore amended the review protocol to remove this part of the analysis. In addition, we mentioned using Holm-Bonferroni method in our protocol, but we did not apply it in our analysis.

### Search strategy and selection of studies

We searched eight databases (Embase, Scopus, MEDLINE, CINAHL, BIOSIS, Cochrane, Central, and DARE) for original articles concerning the effects of NSAID on fractured bone healing in animal models. We considered the period from the starting date of each database to 1 February 2021. The main terms used in the search strategy, developed with the help of the Liaison Librarian for Life Sciences at McGill University, were “anti-inflammatory agents,” “non-steroidal,” “bone,” and “animals” (the complete search strategy is provided in Supplementary material Table S2). We used a search filter to detect animal studies and exclude human studies. Although we did not impose language restrictions while searching, only English articles were reviewed. Also, we did not include conference abstracts because they do not provide sufficient data to allow for an evaluation. No other restrictions were used. Additionally, we hand searched the reference lists of eligible articles, and screened review articles for relevant references. The first selection was performed based on independent selection by two reviewers (HA, AP) using RAYYAN software (httpp://rayyan.org, Doha, State of Qatar) [34]. Any disagreement was solved by discussion or by including the third reviewer (BN). Full-text articles were screened and included when they met the following pre-specified criteria: (1) a controlled NSAID interventional design after bone fracture; and (2) a description of outcome measures related to bone healing (biomechanical characteristics, μ-CT scan measures, radiographic bone assessment, and/or histomorphometric and histological based grading). Papers were excluded if they fulfilled one of the following criteria: not an original article (e.g., review or letter), use of a bone graft or other material, included only outcomes that were not biomechanical or histomorphometric, not NSAID used, not experimental animal studies, and duplicate studies. A list of articles excluded as well as the reasons for exclusion are available from the authors upon request.

### Data extraction

The final set of articles was assessed independently by two reviewers who extracted the data using DistillerSR (Evidence Partners, Ottawa, Canada) following a piloted data extraction method. Disagreements were resolved by consensus.

Data retrieved from the articles included characteristics of the animals (e.g., animal model used, weight and sex), test methods (bone fracture [e.g., site, type, number and technique used to perform the fracture, whether there was fixation or not and type of fixation method]), use of medication (e.g., opioid, antibiotics, and NSAID [e.g., type, name, duration of use and route of administration]), and outcome data (time points of outcome measures collection, type of outcome). We grouped the outcomes into main classes: (i) *biomechanical* (e.g., maximum force or ultimate force load, stiffness, work-to-failure), (ii) μ-CT assessment of healing (volume and density), and (iii) *histomorphometric characterization* of fracture callus (bone, cartilage, and mineralized tissue).

Raw data or group averages (mean, median), standard deviation (SD), standard error (SE), or ranges and number of animals per group (*n*) were extracted for all continuous outcome measures. We contacted the authors to obtain original data if results were presented graphically or were incomplete. If data could not be retrieved, the study was excluded from further analysis.

### Risk of bias assessment

Two blinded reviewers (HA, AM) assessed the internal validity of the included studies using SYRCLE’s risk of bias tool (Table S3) [35]. The tool, an adaptation of the Cochrane risk-of-bias tool, considers aspects of bias specific to animal studies. It contains 10 entries related to 6 types of bias (selection, performance, detection, attrition, reporting, and other bias). The score (yes) indicates a low risk of bias, (no) indicates a high risk of bias, and (?) indicates an unclear risk of bias. We were concerned that many items would be rated as having an unclear risk of bias because of the known poor reporting of experimental designs [36]. To overcome this problem, we added four entries to the tool, pertaining to randomization, blinding, sample size calculation, and time of day of the NSAID administration or time of day at which surgery was performed [35]. For these items, ‘yes’ and ‘no’ indicates reported and unreported, respectively.

### Data analysis and synthesis

The meta-analysis was conducted according to Preferred Reporting Items for Systematic Reviews and Meta-Analyses (PRISMA) guidelines, and using Comprehensive Meta-Analysis software (version 2.2.064, Biostat Inc., Englewood) when five or more independent comparisons from at least three different studies per outcome category were included (provided that outcome measure assessments were sufficiently comparable). We calculated the standardized mean differences (SMD) through Hedges g effect sizes [37]. The calculation was (SMD = the mean of the NSAIDs group minus the mean of the control vehicle group divided by the pooled standard deviations of the two groups) to account for the differences in the units of measurements. We used Hedges *g* effect to calculate the SMD, Hedge’s *g* (which is based on Cohen’s *D* but includes a correction factor for small sample size bias) [38, 39]. Again, the calculations need to take into account the direction of effect.

Despite the anticipated heterogeneity, the individual effect sizes were subsequently pooled to obtain an overall SMD and 95% confidence interval (95%CI) [38, 40]. We used a random-effects model [40], which takes into account the precision of individual studies and the variation among them, and weights each study accordingly. If multiple independent experimental groups were compared to the same control group within the meta-analysis, the number of animals in the control group was corrected by dividing it by the number of experimental groups.

Rather than computing a single summary measure, an important objective of meta-analysis is to explore the sources of heterogeneity [41], a measure of the degree of variability in study results, and assess which variables influence the effect of NSAID on bone healing outcomes. We conducted subgroup analyses according to sex and animal species (mice, rats, and rabbits), type of NSAID (non-selective/COX-2 selective), type of fracture, and period of outcome measurement or data collection (early healing less than 21 days, 21 to 48 days, and more than 48 days). We present below the results for subgroups containing at least 10 comparisons. A minimum of three independent comparisons per subgroup was needed to record the subgroup characteristics. The interpretation of differences between subgroups should be used mainly to construct new hypotheses rather than drawing definite conclusions. Heterogeneity for subgroup analyses was assessed using *I*^2^ and the *Q* statistic.

The subgroup analysis was done and generated through three methods for comparing the effect size across subgroups. One method was to use a *Z*-test to compare the effect sizes directly. Another method was to use a *Q*-test to partition the variance and test the between-subgroups portion of the variance. A third one was to use a *Q*-test to assess the dispersion of the summary effect about the combined effect. All the methods used by the software assess the variance across subgroups effects relative to the variance within subgroups. The row tables for each subgroup analysis generated by the comprehensive meta-analysis software were added in the supplementary information file for detailed results based on the previous described methods (Tables S5 to S27). We assessed publication bias for different healing outcomes by evaluating the possible asymmetry using funnel plots Trim and fill and “Egger’ regression test” if the analysis contained at least 20 comparisons” [38]. All statistical analysis was conducted using Comprehensive Meta-Analysis Version 3 Software (NJ, USA) [42]. A *P* value less than 0.05 was considered to be statistically significant.

## Results

### Study selection and characteristics

**Fig. 1:**
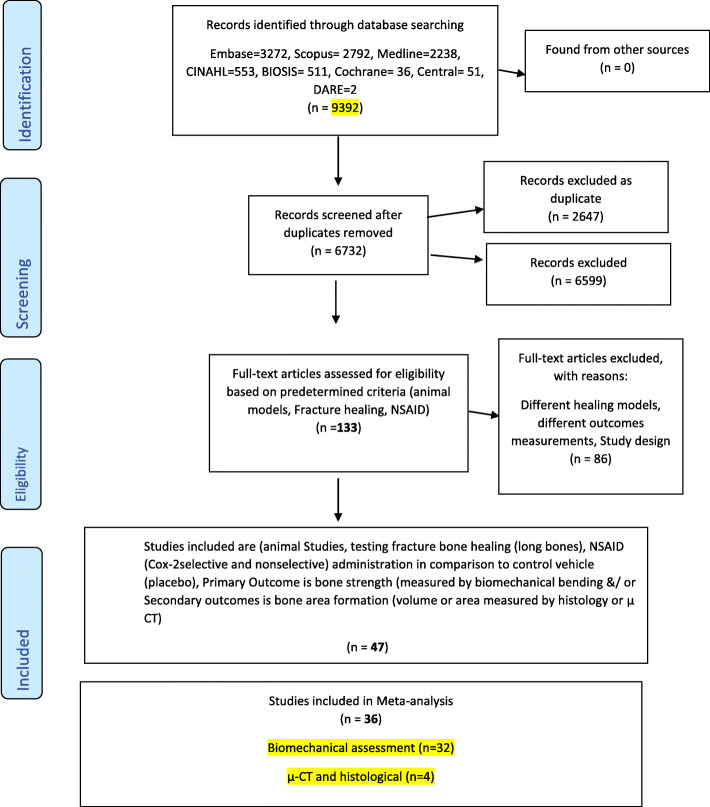
PRISMA flow diagram of the selection of the studies for the systematic review and meta-analysis included

After the full-text assessment, 47 publications were included in the systematic review (see Fig. 1 for the PRISMA flowchart). The authors were contacted when we could not retrieve or understand the data and only two out of ten responded to the request and sent the raw data. Table S4 presents characteristics of the included studies (the complete list is available in Supplementary file 1). Overall, study characteristics varied considerably; most studies were performed in rats (31 studies; 66%), 5 in mice (10.6), 9 in rabbits (19.1%), and 2 in dogs. Eight studies did not report the sex of the animal, while 26 (55.3) and 13 (27.7%) used only male or female animals, respectively, and none used both sexes. Eleven studies (23.4%) used a selective COX-2 NSAID as an experimental intervention drug, 22 (46.8%) and 14 (29.8%) used a non-selective or both NSAID types, respectively. There was a great variability on the primary outcomes for biomechanical characteristics; 37 (78.7%) studies reported 1 or more biomechanical characteristics, and 10 (21.2%) studies did not report any of these characteristics.

### Risk bias and quality of the studies

**Fig. 2:**
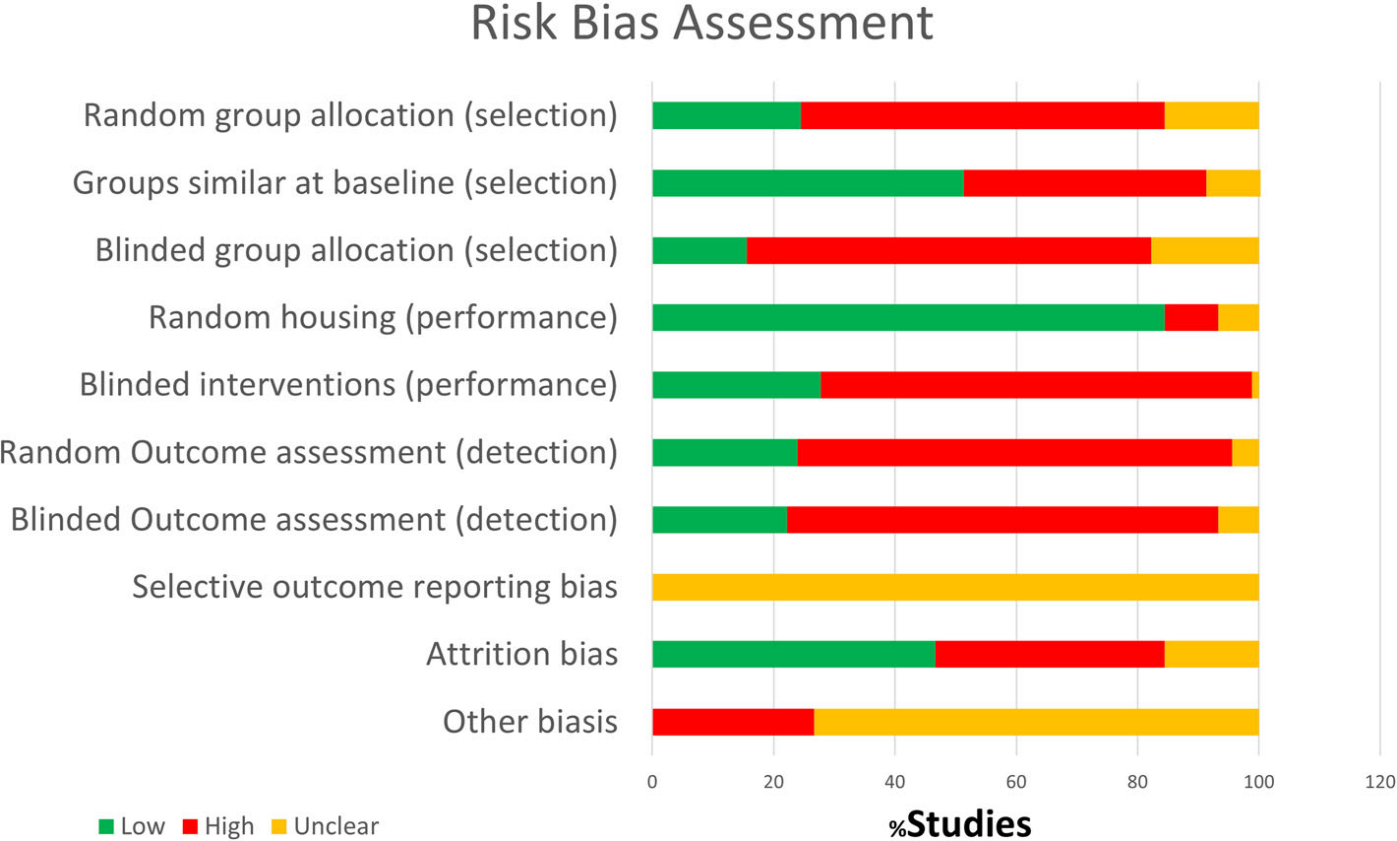
Risk of bias assessment using SYRCLE risk of bias assessment tool

**Fig. 3:**
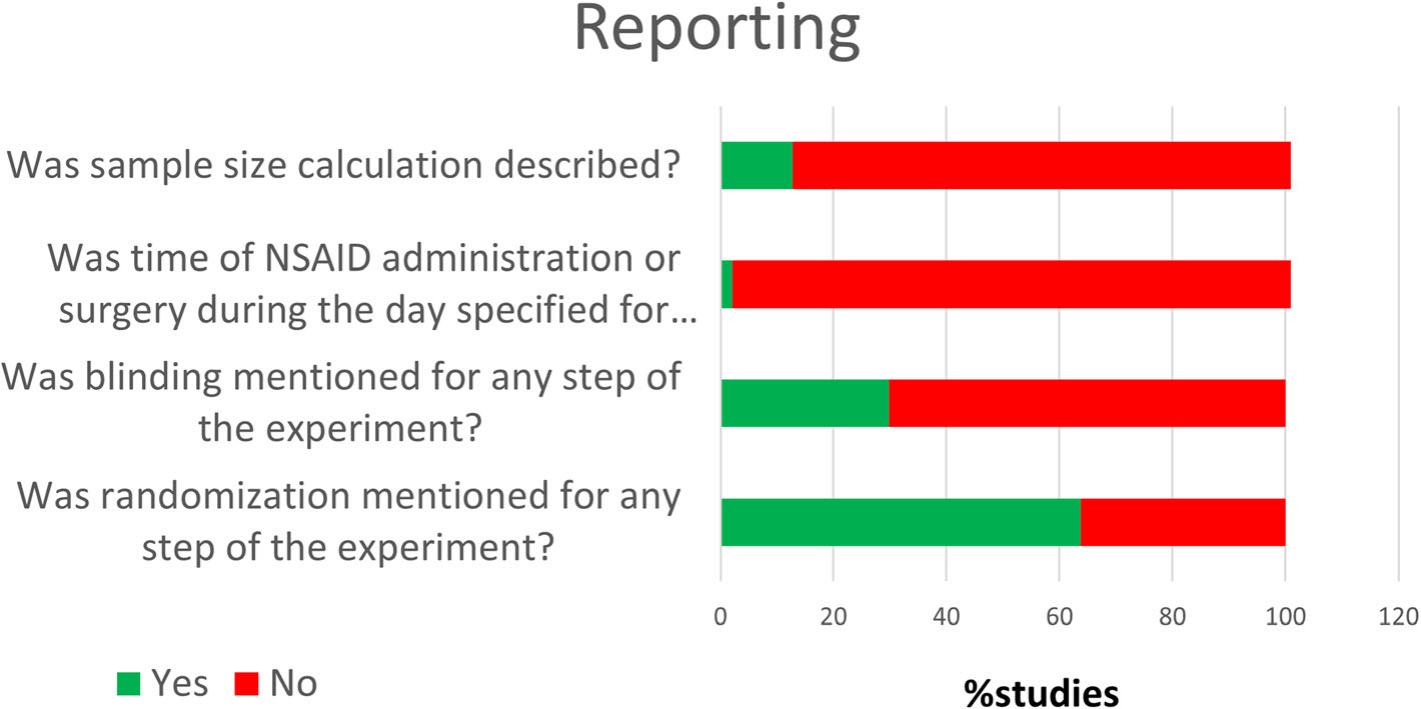
Quality of reporting assessment of the included studies

The assessment results for risk of bias and quality of reporting related to randomization, blinding, sample size calculation, and time of day for NSAID administration or surgery are summarized in Figs. 2 and 3. Scores for each study are presented in Supplementary file 2. Among the 47 included studies, 30 (63.8%) mentioned the term “randomization” at any step in the study, but no article provided details on the method used. Only 14 (29.8%) studies reported blinding which for most of them was on the histological outcome assessment. Among all included studies, only 6 (12.8%) reported a sample size calculation; 1 article specified the time of day at which NSAID was administered or the time that surgery was performed (day or night). Due to poor reporting, many items evaluating the risk of bias on the assessment tool showed an unclear score. For example, “selective outcome reporting bias” was assessed as unclear for all studies because none reported using a research protocol defining primary and secondary outcomes.

For more details regarding each included study (*n* = 47) in the systematic review and the risk of bias assessment for each parameter is presented in the Supplementary file 2 including how many studies with high, low, or unclear for each parameter of the tool.

### Meta-analysis of NSAID administration during fractured bone healing

Due to missing information in 11 studies regarding outcome data, or not suitable outcome measurement, or the intervention is not fracture, or the drug was not NSAID or was given with other analgesic such as opioid or steroidal anti-inflammatory for the same group during the course of the study [6, 43–50], we included 36 studies in the meta-analysis. Thirty-two studies compared the effect of administration of one or more NSAID on biomechanical characteristics (e.g., maximum force (MF) to fracture, stiffness, and work-to-failure) to a control group. For three-point mechanical bending properties, the analysis includes 186 experiments covering different animal models, NSAID types, and measurement time points. Four and seven studies were included in the analysis of the effect of NSAID administration on the μ-CT and histological assessment healing outcomes, respectively. The average timing of data collection after bone fracture to assess the mechanical bending maximum force of healing bones was an average of 29.6 days (minimum, 5 days; maximum, 84 days).

### Biomechanical assessment

**Fig. 4:**
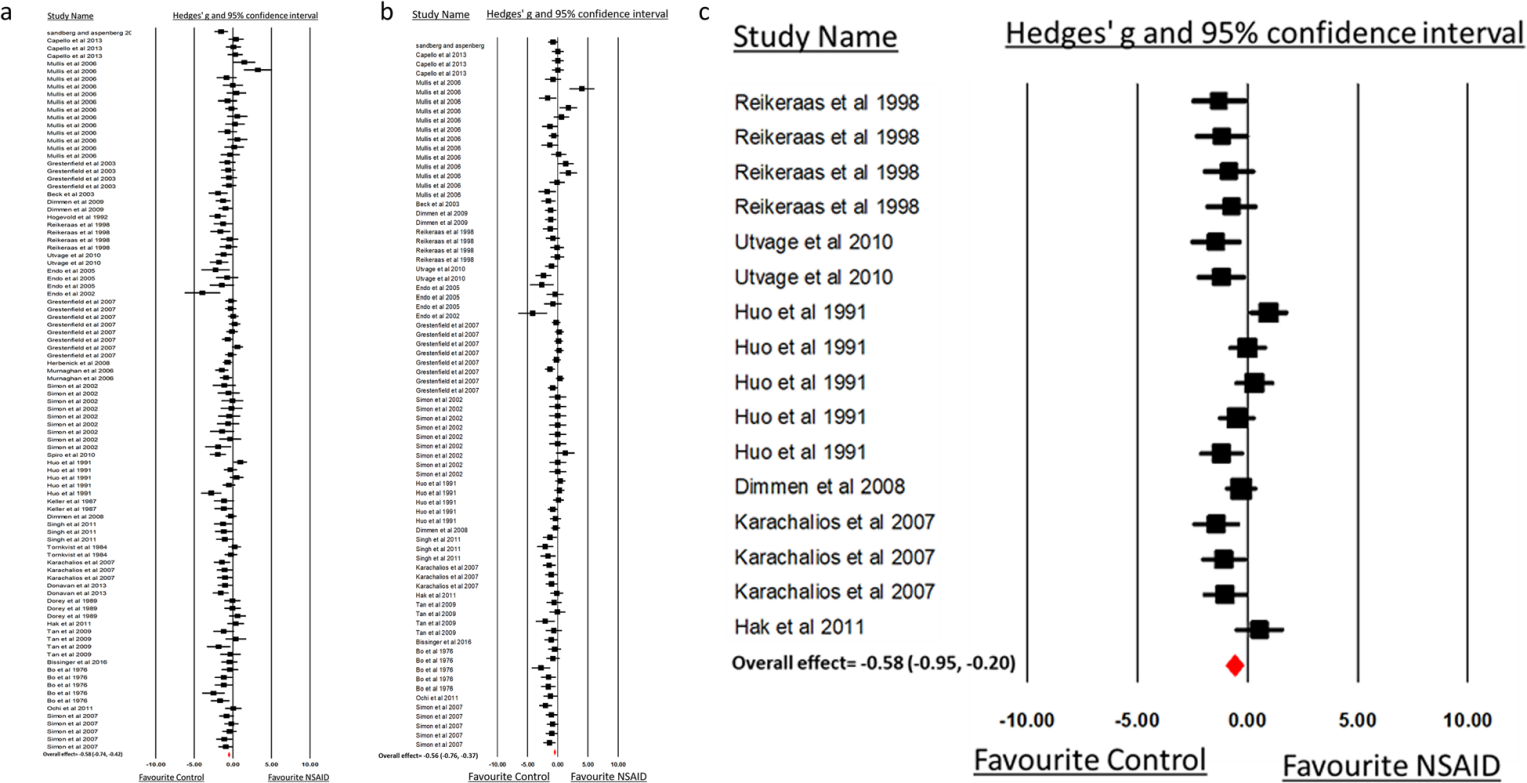
Forest plot of the included studies (experimental groups), which used three-point mechanical bending **a** maximum force (MF), **b** stiffness, **c** work-to-failure. The forest plot displays the standard mean differences (SMDs) Hedges’ *g*, 95% confidence interval. The diamond indicates the overall estimation and its 95% confidence interval

Results from 30 studies including 94 comparisons showed that the maximum force to fracture was significantly decreased, indicating bone healing delay, in animals that received an NSAID after bone fracture compared to the control group (SMD − 0.58, 95%CI [− 0.74, − 0.42]; Table 1). Heterogeneity was moderate (*I*^2^, 55.04%). Similarly, animals that received NSAID had an overall decrease in bone stiffness and work-to-failure properties (SMD − 0.56 [− 0.76, − 0.37] and SMD − 0.58 [− 0.95, − 0.20]) respectively compared to controls (Fig. 4; Table 1). Between-study heterogeneity was moderate for both stiffness (*I*^2^, 60.41%) and work to failure outcomes (*I*^2^, 56.29%).

**Table 1:** Meta-analysis based on several subgroups showing the effect of NSAID administration on three-points mechanical bending measurements (maximum force, stiffness, and work to failure) of healing bones after fracture

Subgroup	*N*	*SZ* *NSAID*	*SZ* *C.*	*SMD*	*95% CI*	*P effect(adj. P value)*	*Q statistic*	*P within heterog.*	*I*^*2*^*%*	*P between heterog.*
Maximum Force NSAID vs Control										
Overall	94	995	594	− 0.58	[− 0.74, − 0.42]	0.000	206.89	< 0.001	55.04	–
*Species*										
Rats	56	655	370	− 0.64	[− 0.85, − 0.43]	0.000(< 0.001)	117.18	< 0.001	53.06	0.336
Mice ^a^	17	181	95	− 0.28	[− 0.68, 0.10]	0.154(0.205)	54.53	< 0.001	70.66	
Rabbits	20	153	124	− 0.66	[− 1.02, − 0.31]	0.000(< 0.001)	32.9	0.025	42.26	
Dogs	1	–	–	NA	NA	NA	–	–	NA	
*Sex*										
Female	22	261	148	− 0.61	[− 0.95, − 0.28]	0.000(< 0.001)	62.93	< 0.001	66.63	0.45
Male	63	667	377	− 0.52	[− 0.72, − 0.32]	0.000(< 0.001)	129.09	< 0.001	51.97	
Not mentioned	9	67	70	− 0.87	[− 1.38, − 0.35]	0.000(< 0.001)	12.34	0.136	35.2	
*Age/weeks*										
< 8	3	24	21	0.26	[− 0.56, 1.09]	0.536(0.536)	0.249	0.883	0	0.023^a^
8–16	39	471	270	− 0.40	[− 0.64, − 0.16]	0.001(0.002)	93.64	< 0.001	59.41	
> 16	14	120	63	− 0.66	[− 1.10, − 0.21]	0.003(0.004)	14.514	0.339	10.43	
Not mentioned	38	380	241	− 0.81	[− 1.05, − 0.56]	0.000(< 0.001)	80.64	< 0.001	54.12	
*Type of NSAID*										
NS-COX	53	528	349	− 0.55	[− 0.76, − 0.34]	0.000(< 0.001)	122.32	< 0.001	57.48	0.695
S-COX2	41	467	245	− 0.61	[− 0.86, − 0.37]	0.000(< 0.001)	83.79	< 0.001	52.26	
*Time point*										
< 21 days	20	214	123	− 0.62	[− 0.97, − 0.27]	0.001(0.002)	51.81	< 0.001	63.33	0.957
21–48 days	58	628	343	− 0.54	[− 0.75, − 0.33]	0.000(< 0.001)	124.99	< 0.001	54.39	
> 48 days	15	129	104	− 0.65	[− 1.07, − 0.24]	0.002(0.003)	29.25	0.01	52.15	
NM	1	–	–	NA	NA	NA	–	–	NA	
*Type of fractured bone*										
Femur	57	662	409	− 0.68	[− 0.88, − 0.48]	0.000(< 0.001)	120.03	< 0.001	53.34	0.01^a^
Tibia^a^	28	264	133	− 0.19	[− 0.49, 0.10]	0.2(0.2)	62.49	< 0.001	56.79	
Fibula	4	27	11	− 0.69	[− 1.55, 0.16]	0.115(0.153)	5.67	0.128	47.15	
Ulna	5	42	42	− 1.20	[− 1.86, − 0.55]	0.000(< 0.001)	1.03	0.906	0	
Stiffness NSAID vs control										
Overall	76	809	441	− 0.56	[− 0.76, − 0.37]	0.000	189.46	< 0.001	60.41	–
*Species*										
Rats	50	582	318	− 0.57	[− 0.80, − 0.33]	0.000(< 0.001)	109.04	< 0.001	55.06	0.046^a^
Mice ^a^	14	145	59	− 0.07	[− 0.55, 0.40]	0.758(0.785)	55.63	< 0.001	76.63	
Rabbits	11	76	58	− 1.06	[− 1.58, − 0.53]	0.000(< 0.001)	11.14	0.34	10.3	
Dogs	1	–	–	NA	NA	NA	–	–	NA	
*Sex*										
Female	18	223	124	− 0.82	[− 1.21, − 0.44]	0.000(< 0.001)	40.88	0.001	58.41	0.022^a^
Male	55	568	299	− 0.42	[− 0.64, − 0.19]	0.000(< 0.001)	133.42	< 0.001	59.52	
Not mentioned	3	18	18	− 1.63	[− 2.64, − 0.62]	0.002(0.003)	0.78	0.675	0	
*Age/ weeks*										
< 8	3	24	21	0.00	[− 0.89, 0.91]	0.98(0.98)	0	1	0	0.047^a^
8–16	30	373	193	− 0.39	[− 0.7, − 0.08]	0.01().02)	100.04	< 0.001	71.01	
> 16^a^	13	96	36	− 0.31	[− 0.82, 0.18]	0.21(0.28)	18.97	0.08	36.75	
Not mentioned	30	316	188	− 0.88	[− 1.18, − 0.58]	0.000(< 0.001)	54.75	0.003	47.03	
*Type of NSAID*										
NS-COX	40	411	255	− 0.41	[− 0.67, − 0.15]	0.002(0.003)	78.83	< 0.001	50.52	0.091
S-COX2	36	398	186	0.75	[− 1.04, − 0.46]	0.000(< 0.001)	102.1	< 0.001	65.77	
*Time point*										
< 21 days	14	160	84	− 0.99	[− 1.43, − 0.54]	0.000(< 0.001)	21.48	0.06	39.49	0.085
21–48 days	51	560	292	− 0.42	[− 0.66, − 0.19]	0.000(< 0.001)	144.11	< 0.001	65.3	
> 48 days	11	89	64	− 0.65	[− 1.16, − 0.13]	0.014(0.01)	12.11	0.27	17.46	
*Type of Fractured bone*										
Femur	46	531	299	− 0.59	[− 0.84, − 0.34]	0.000(<0.001)	100.78	< 0.001	55.34	0.458
Tibia	23	227	107	− 0.39	[− 0.76, − 0.02]	0.038(0.05)	78.93	< 0.001	72.12	
Fibula	4	27	11	− 0.77	[− 1.71, 0.16]	0.107(0.1)	3.95	0.267	24.06	
Ulna	3	24	24	− 1.16	[− 2.11, 0.20]	0.017(0.034)	0.32	0.848	0	
Work to failure NSAID vs control										
Overall	16	148	121	− 0.58	[− 0.95, − 0.20]	0.002	34.32	0.003	56.29	–
*Species*										
Rats	12	117	92	− 0.52	[− 0.96, − 0.09]	0.017(0.034)	25.28	0.008	56.49	0.619
Rabbits	4	31	29	− 0.75	[− 1.51, 0.01]	0.054(0.054)	7.72	0.052	61.18	
*Sex*										
Female^a^	6	65	66	− 0.09	[− 0.55, 0.36]	0.694(0.0694)	12.59	0.028	60.29	0.007^a^
Male	10	83	55	− 0.94	[− 1.37, − 0.52]	0.000(< 0.001)	9.63	0.381	6.59	
*Age/weeks*										
< 8	0	–	–	–	–	–	–	–	–	0.267
8–16	3	24	24	− 1.16	[− 1.99, − 0.32]	0.006(0.007)	0.32	0.848	0	
> 16	3	27	15	− 0.67	[− 1.54, 0.20]	0.131(0.131)	7.42	0.024	73.07	
Not mentioned	10	97	82	− 0.38	[− 0.83, 0.05]	0.089(0.118)	19.88	0.019	54.73	
*Type of NSAID*										
NS-COX	11	411	255	− 0.58	[− 1.05, − 0.11]	0.015(0.03)	26.52	0.003	62.30	0.99
S-COX2^a^	5	398	186	− 0.58	[− 1.26, 0.09]	0.092(0.092)	7.72	0.102	48.18	
*Time point*										
< 21 days	1	–	–	NA	NA	NA	–	–	NA	0.03^a^
21–48 days	12	102	75	− 0.71	[− 1.10, − 0.32]	0.000(< 0.001)	19.54	0.052	43.73	
> 48 days	3	35	35	− 0.59	[− 1.26, 0.07]	0.083().12)	2.174	0.33	8.00	
*Type of fractured bone*										
Femur^a^	10	97	82	− 0.38	[− 0.83, 0.05]	0.089(0.12)	19.88	0.019	54.73	0.267
Tibia	3	27	15	− 0.67	[− 1.54, 0.20]	0.131(0.131)	7.42	0.024	73.07	
Ulna	3	24	24	− 1.16	[− 1.99, − 0.32]	0.006(0.018)	0.32	0.848	0	

*N* number of comparisons in analysis, *SZ NSAID* number of animals in non-steroidal anti-inflammatory drug (NSAID) group, *SZ C*. number of animals in control group, *SMD* standardized means of differences (Hedges’ *g*), *CI* confidence interval, *NS-COX* non-selective cyclooxygenase inhibitor, *S-COX2* selective cyclooxygenase 2 inhibitor, *NA* not analyzed because of insufficient data, *adj.P value* adjusted *P* value through Holm-Bonferroni correction

^a^Need to be explained—more details in Tables S5–S22 (supplementary information file)

We explored the sources of heterogeneity by examining the effect sizes in predefined subgroups: animal sex, age and species, time of bone collection, and type of fractured bone. While animal age and type of bone were source of heterogeneity for the maximum force to break, time of sample collection and animal sex, age, and species were for the stiffness analysis. Moreover, sex and time of sample collection were sources of heterogeneity in the work to failure analysis (Table 1).

**Fig. 5:**
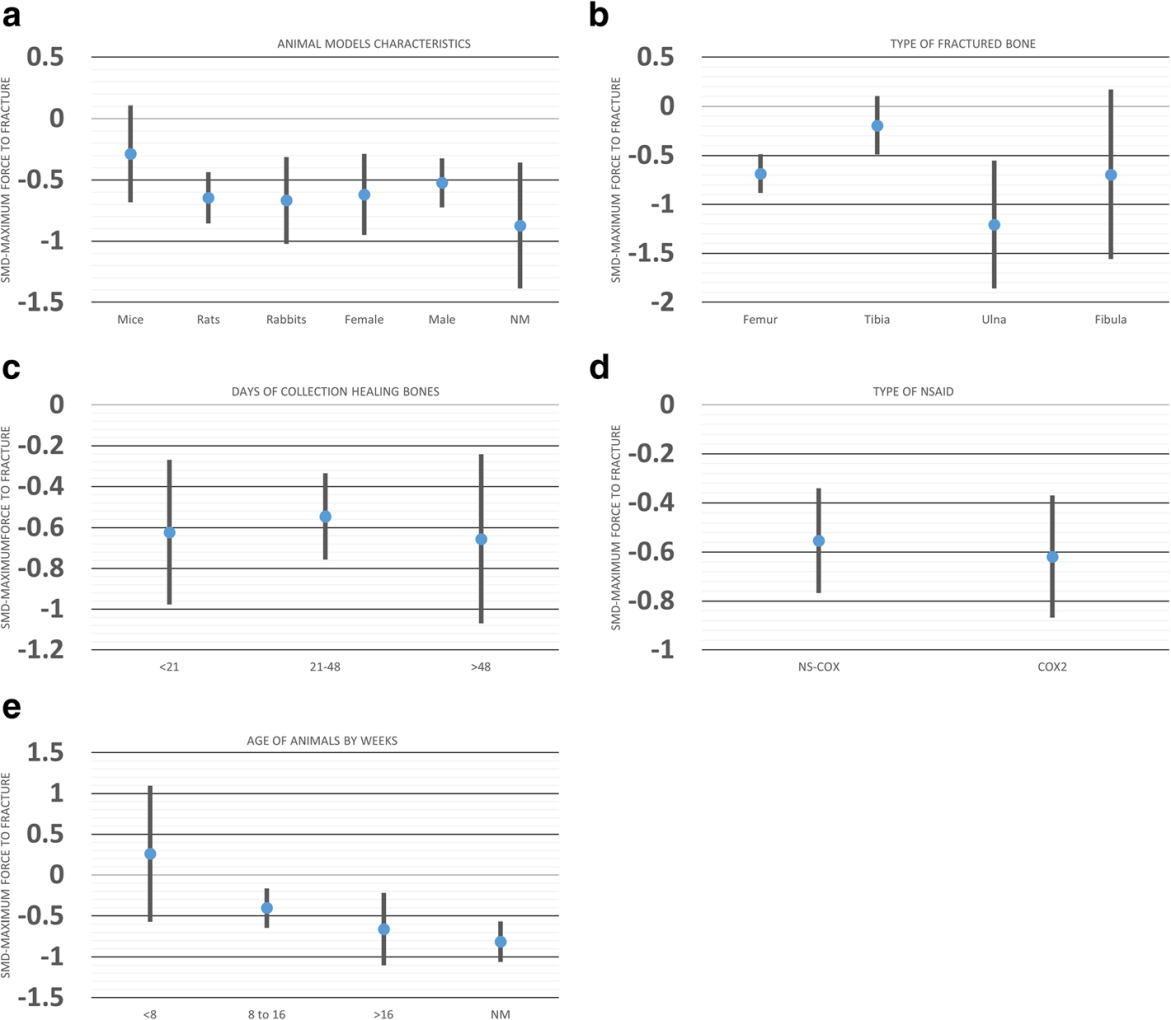
Effect of **a** animal models’ characteristics, **b** type of fractured bone, **c** time of collection healing bones, **d** type of NSAID, and **e** age of the animals on biomechanical bending measurements (maximum force to fracture) (MF) after administration of NSAID compared to the administration of a control vehicle. The columns indicate the effect estimate with the 95% confidence interval of the subgroups. SMD, standard mean difference–Hedges’ *g* mean. NM, not mentioned; NS-COX, non-selective cyclooxygenase inhibitor; COX2, selective-cyclooxygenase2 inhibitor

Table 1 shows the subgroup analysis for three-point mechanical bending measurements. For maximum force measurement, NSAID administration did not delay bone healing among mice (SMD − 0.28 [− 0.68, 0.10]) but did it in other animals. In addition, we observed a difference in this measurement for the subgroup analysis of bone model, while femur (SMD − 0.68 [− 0.88, − 0.48]) showed a significant difference between NSAID and control, tibia did not (SMD − 0.19 [− 0.49, 0.10]). Moreover, when comparing SMD across bone models, the effect of NSAID administration was significantly larger in femur compared to tibia (*P* = 0.007; Fig. 5b).

**Fig. 6:**
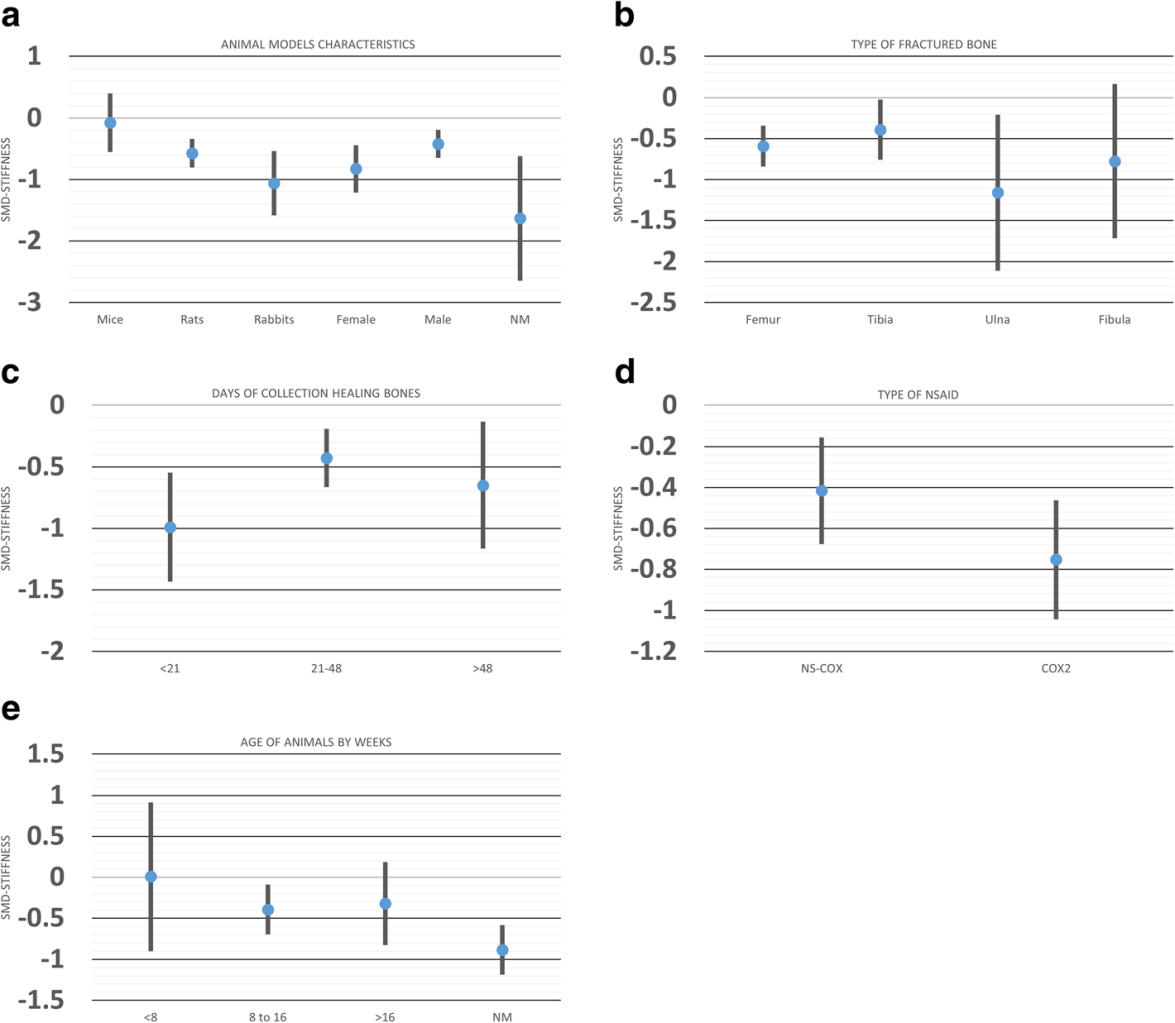
Effect of **a** animal models’ characteristics, **b** type of fractured bone, **c** time of collection healing bones, **d** type of NSAID, and **e** age of the animals on biomechanical bending measurements (stiffness) after administration of NSAID compared to the administration of a control vehicle. The columns indicate the effect estimate with the 95% confidence interval of the subgroups. SMD, standard mean difference–Hedges’ *g* mean. NM, not mentioned; NS-COX, non-selective-cyclooxygenase; COX2, selective-cyclooxygenase 2 inhibitor

Bone stiffness among mice (SMD − 0.07 [− 0.55, 0.40]) and animals older than 16 weeks (SMD − 0.31 [− 0.82, 0.18]) did not differ between NSAID and control groups (Table 1). However, compared to controls, bone healing was better in mice taking NSAIDs than in rabbits (*P* = 0.01; Fig. 6a). The effect of NSAID administration was significantly different when the bone samples were harvested before 21 days compared to other time points between 21 and 48 days after surgery (*P* = 0.03; Fig. 6c).

**Fig. 7:**
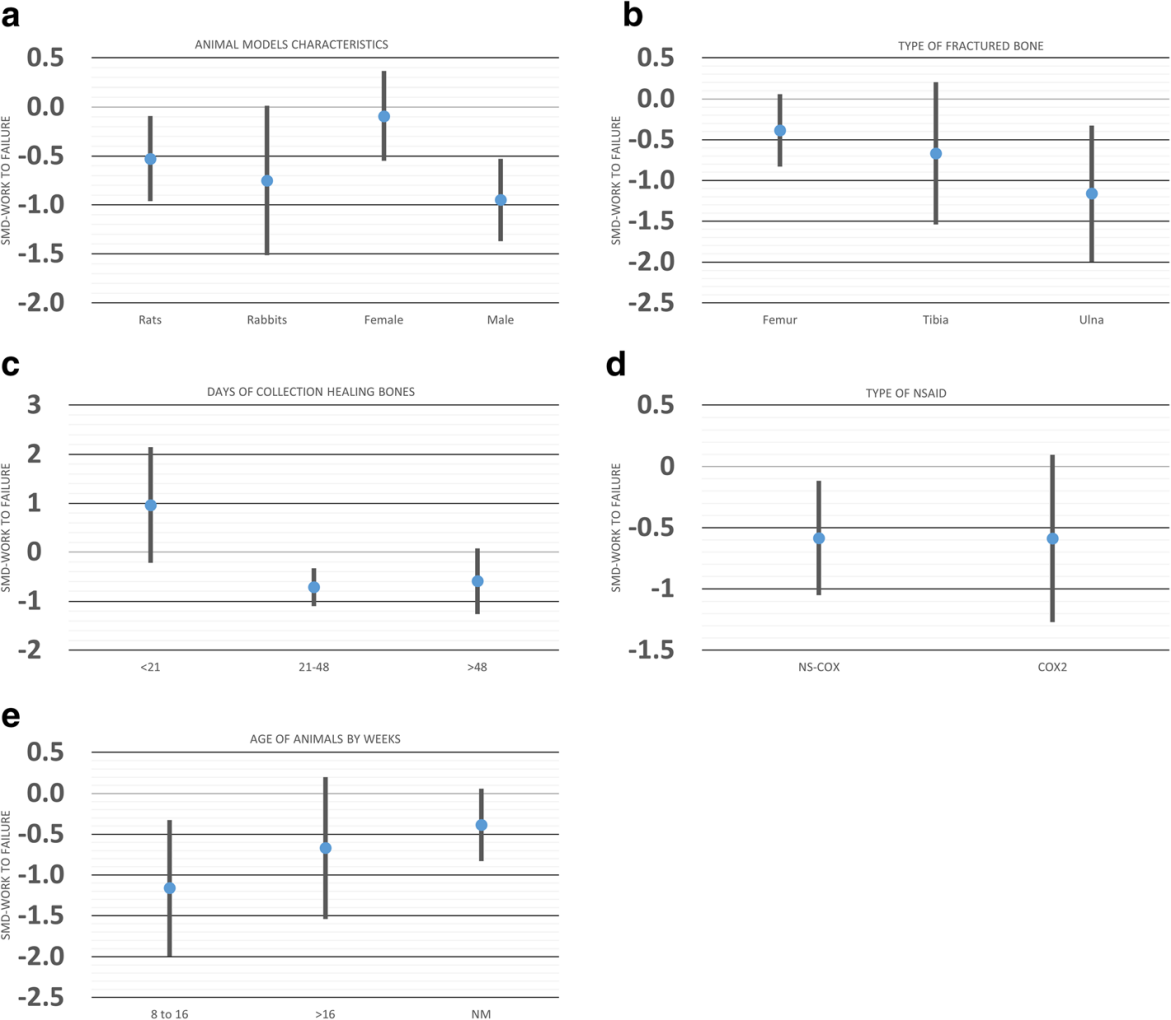
Effect of **a** animal models’ characteristics, **b** type of fractured bone, **c** time of collection healing bones, **d** type of NSAID, and **e** age of the animals on biomechanical bending measurements (work to failure) after administration of NSAID compared to the administration of a control vehicle. The columns indicate the effect estimate with the 95% confidence interval of the subgroups. SMD, standard mean difference–Hedges’ *g* mean. NM, not mentioned; NS-COX, non-selective-cyclooxygenase; COX2, selective-cyclooxygenase 2 inhibitor

Regarding work to failure, there was no significant effect of NSAID administration on bone healing in the groups of female animals (SMD − 0.09 [− 0.55, 0.36]), those that received selective-cyclooxygenase2 NSAID (SMD, − 0.58 [− 1.26, 0.09]), and for the femur bone model fracture (SMD, − 0.38 [− 0.83, 0.05]) (Table 1; Fig. 7).

### Micro-computed tomography assessment (bone assessment)

**Fig. 8:**
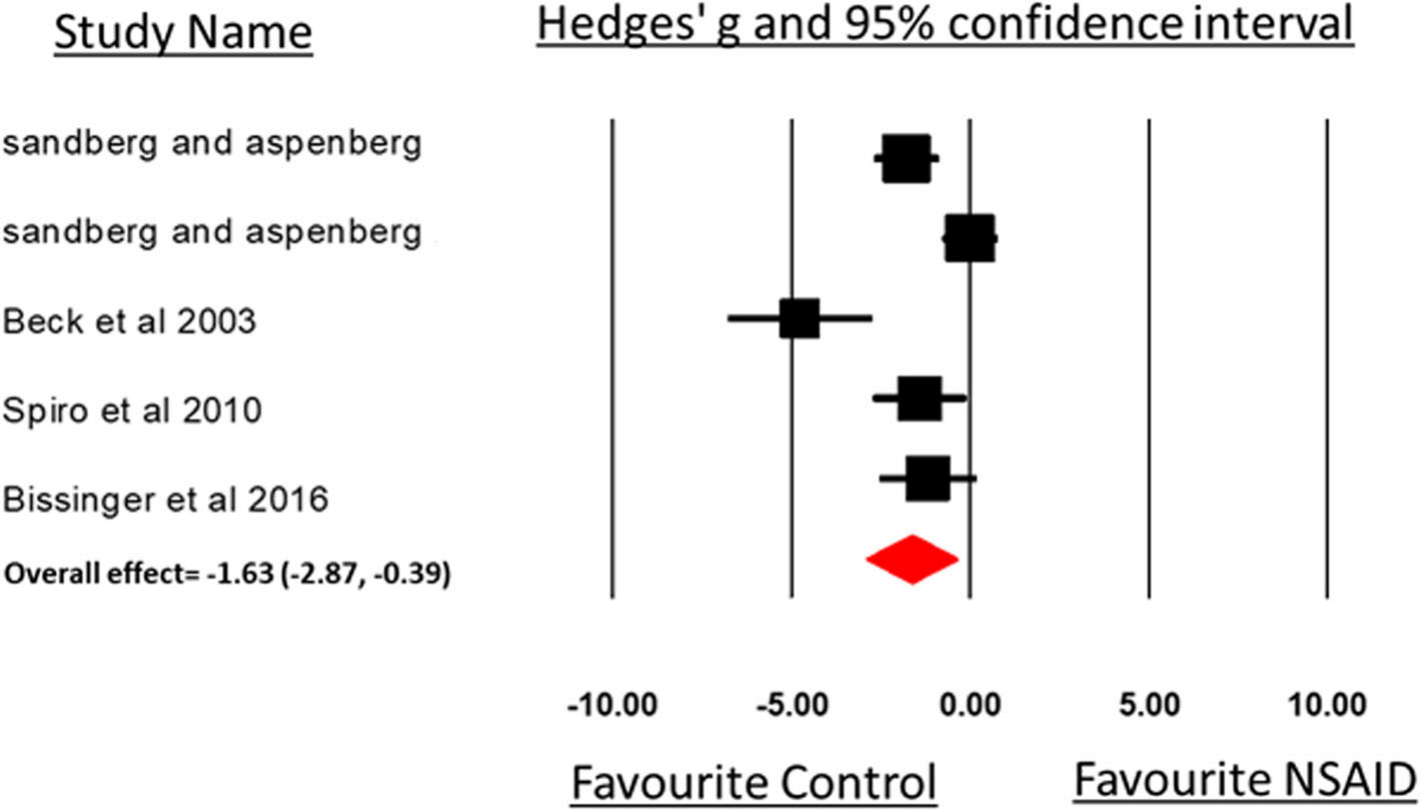
Forest plot of the included studies (experimental groups), which used micro CT analysis bone volume and bone density measurements. The forest plot displays the standard mean differences (SMDs) Hedges’ *g*, 95% confidence interval. The diamond indicates the global (overall) estimation and its 95% confidence interval

We included five comparisons from four studies that measured healing bone using a μ-CT scan in the meta-analysis. The average time of bone collection after animal euthanasia was 19.5 days (range, 17–21 days). Figure 8 and Table 2 show the distribution of the data. Although the subgroup analyses were not performed because the number of comparisons was small, the overall analysis shows a significant difference in bone volume measurements for animals that received NSAID compared to controls (SMD, − 1.63 [− 2.87, − 0.39]), but this was associated with high heterogeneity among the studies (*I*^2^ 83.32, *P* < 0.001).

**Table 2:** Meta-analysis based on several subgroups showing the effect of NSAID administration on bone volume and density measurements (μ CT assessment) of healing bones after fracture

μ CT assessment (bone) NSAID vs control										
Subgroup	*N*	*SZ* *NSAID*	*SZ* *C.*	*SMD*	*95% CI*	*P effect*	*Q statistic*	*P withinheterog.*	*I*^*2*^*%*	*P betweenheterog.*
Overall	5	48	39	− 1.63	[− 2.87,− 0.39]	0.01	23.98	< 0.001	83.32	–
*Species*										
Rats	2	7	3	NA	NA	NA	NA	NA	NA	0.193
Mice	3	31	31	− 1.03	[− 2.55, 0.48]	0.18	9.95	0.007	0	
*Sex*										
Female	1	5	5	NA	NA	NA	NA	NA	NA	0.851
Male	4	43	43	− 1.73	[− 3.06, − 0.27]	0.029	23.755	< 0.001	87.37	
*Age/weeks*										
< 8	0	NA	NA	NA	NA	NA	NA	NA	NA	0.007
8–16	4	38	34	− 1.04	[− 1.98, − 0.10]	0.03	10.157	0.017	70.46	
> 16	0	NA	NA	NA	NA	NA	NA	NA	NA	
Not mentioned	1	10	5	NA	NA	NA	NA	NA	NA	
*Type of NSAID*										
NS-COX	5	48	39	− 1.63	[− 2.87, − 0.39]	0.01	23.985	< 0.001	83.32	1
S-COX2	0	NA	NA	NA	NA	NA	NA	NA	NA	
*Time point*										
< 21 days	4	38	34	− 1.04	[− 1.98, − 0.10]	0.03	10.157	0.017	70.46	NA
21–48 days	1	NA	NA	NA	NA	NA	NA	NA	NA	
> 48 days	0	NA	NA	NA	NA	NA	NA	NA	NA	
*Type of fractured bone*										
Femur	4	38	34	− 1.04	[− 1.98, − 0.10]	0.03	10.157	0.017	70.46	0.007
Tibia	1	NA	NA	NA	NA	NA	NA	NA	NA	

*N* number of comparisons in analysis, *SZ NSAID* number of animals in non-steroidal anti-inflammatory drug (NSAID) group, *SZ C*. number of animals in control group, *SMD* standardized means of differences (Hedges’ *g*), *CI* confidence interval, *NS-COX* non-selective cyclooxygenase inhibitor, *S-COX2* selective cyclooxygenase 2 inhibitor, *NA* not analyzed because of insufficient data

### Histomorphometric assessment

**Fig. 9:**
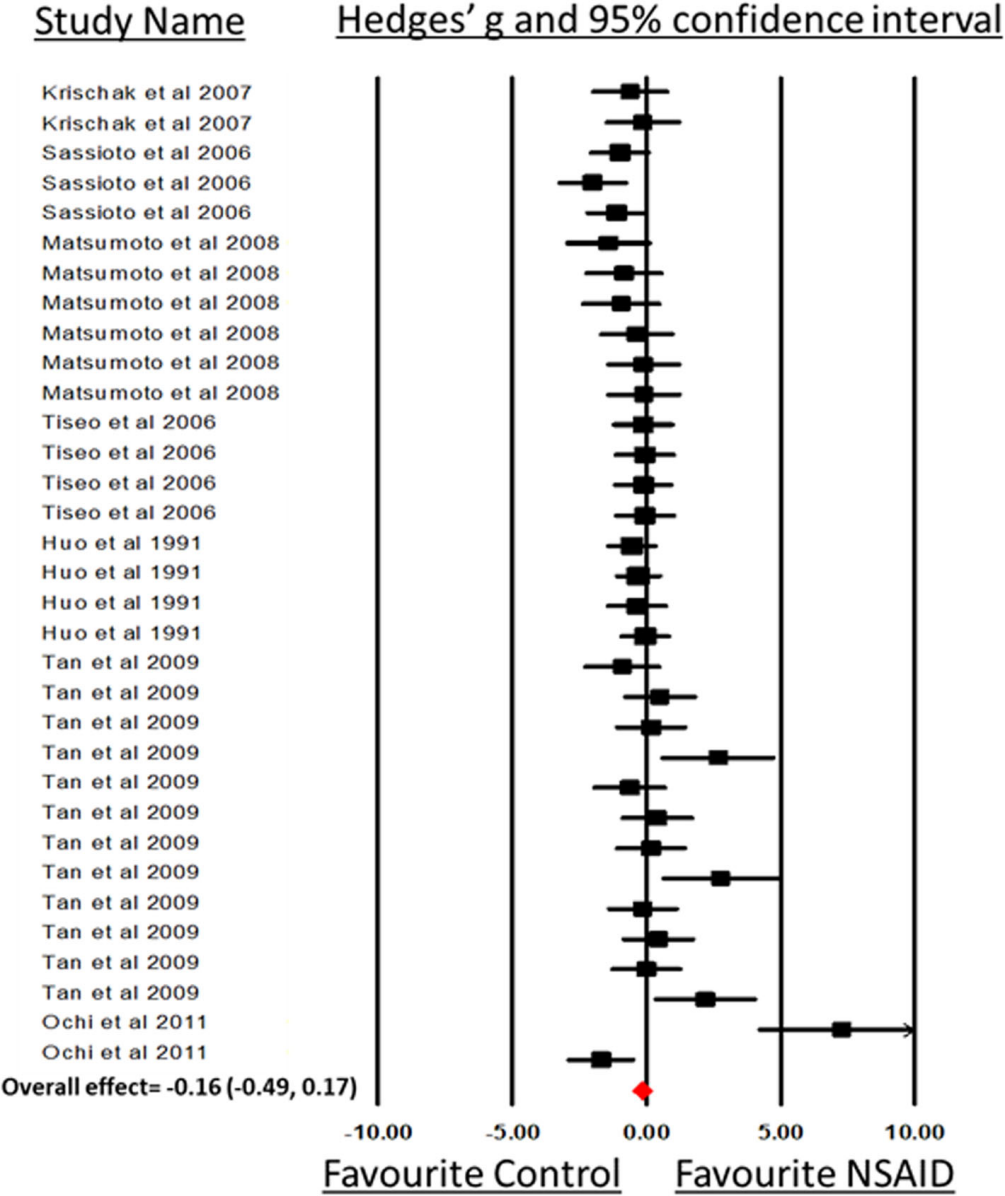
Forest plot of the included studies (experimental groups) that used histomorphometric analysis on microscopic image including callus size, cartilage tissue, bone volume, and bone area measurements. A forest plot displays the standards mean differences (SMDs) Hedges’ *g*, 95% confidence. The diamond indicates the overall estimation and its 95% confidence interval

Seven studies including 33 experimental comparisons between NSAID administration and a control group showed no significant difference in all three (callus size, cartilage, and bone tissue) histomorphometric measurements (SMD, − 0.16 [− 0.49, 0.17], *I*^2^ = 54.64). Animal models and types of fractured bones were sources of heterogeneity for histomorphometric measurements of healing bones among studies (Fig. 9; Table 3). Interestingly, no mouse model was used to study the histomorphometric measurements related to bone, cartilage, or callus size. Rat models and histomorphometric evaluation at less than 21 days showed that bone healing was delayed in the NSAID group compared to controls (Table 3).

**Table 3:** Meta-analysis based on several subgroups showing the effect of NSAID administration on bone volume measurements (histomorphometric analysis) of healing bones after fracture

Histomorphometric NSAID vs control										
Subgroup	*N*	*SZ* *NSAID*	*SZ* *C.*	*SMD*	*95% CI*	*P effect(adj. P value)*	*Q statistic*	*P within heterog.*	*I*^*2*^*%*	*P between heterog.*
Overall	33	171	115	− 0.16	[− 0.49, 0.17]	0.341	70.55	< 0.001	54.64	–
*Species*										
Dogs	2	12	12	NA	NA	NA	NA	NA	NA	0.026^a^
Rats ^a^	19	123	79	− 0.50	[− 0.91, − 0.10]	0.013(0.039)	13.26	0.77	0	
Rabbits	12	36	24	0.42	[− 0.14, 0.98]	0.143(0.143)	19.18	0.058	42.65	
*Sex*										
Female	6	44	43	− 0.14	[− 0.92, 0.72]	0.723(1.08)	28.42	< 0.001	82.4	0.986
Male	23	106	52	− 0.17	[− 0.60, 0.25]	0.427(0.64)	41.66	0.007	47.19	
Not mentioned	4	21	20	− .087	[− 1.01, 0.84]	0.854(0.854)	0.01	1	0	
*Age/weeks*										
< 8	0	NA	NA	NA	NA	NA	NA	NA	NA	0.426
8–16	6	24	12	− 0.60	[− 1.43, 0.22]	0.153(0.612)	2.45	0.783	0	
> 16	2	12	12	NA	NA	NA	NA	NA	NA	
Not mentioned	25	135	91	− 0.09	[− 0.48, 0.29]	0.63(0.63)	38.63	0.03	37.87	
*Type of NSAID*										
NS-COX	22	130	87	− 0.25	[− 0.66, 0.14]	0.214(0.428)	50.72	< 0.001	58.6	0.41
S-COX2	11	41	28	0.04	[− 0.55, 0.65]	0.878(0.878)	18.12	0.053	44.81	
*Time point*										
< 21 days^a^	15	78	46	− 0.50	[− 1.00, − 0.01]	0.042(0.126)	11.97	0.609	0	0.162
21–48 days	15	73	49	0.14	[− 0.36, 0.64]	0.581((0.871)	25.94	0.026	46.04	
> 48 days	3	20	20	0.18	[− 0.99, 1,36]	0.758(0.758)	28.07	< 0.001	92.87	
*Type of fractured bone*										
Femur	11	89	63	− 0.49	[− 0.99, 0.01]	0.055(0.165)	10.40	0.406	3.869	0.041^a^
Tibia	10	46	28	− 0.41	[− 1.03, 0.20]	0.192(0.192)	30.63	< 0.001	70.61	
Fibula	12	36	24	0.42	[− 0.14, 0.98]	0.143(0214)	19.18	0.058	42.65	

*N* number of comparisons in analysis, *SZ NSAID* number of animals in non-steroidal anti-inflammatory drug (NSAID) group, *SZ C*., number of animals in control group, *SMD* standardized means of differences (Hedges’ *g*), *CI* confidence interval, *NS-COX* non-selective cyclooxygenase inhibitor, *S-COX2* selective cyclooxygenase 2 inhibitor, *NA* not analyzed because of insufficient data. *adj.P value*, adjusted *P* value through Holm-Bonferroni correction

^a^Need to be explained—more details in Tables S23–S28 (supplementary information file)

**Fig. 10:**
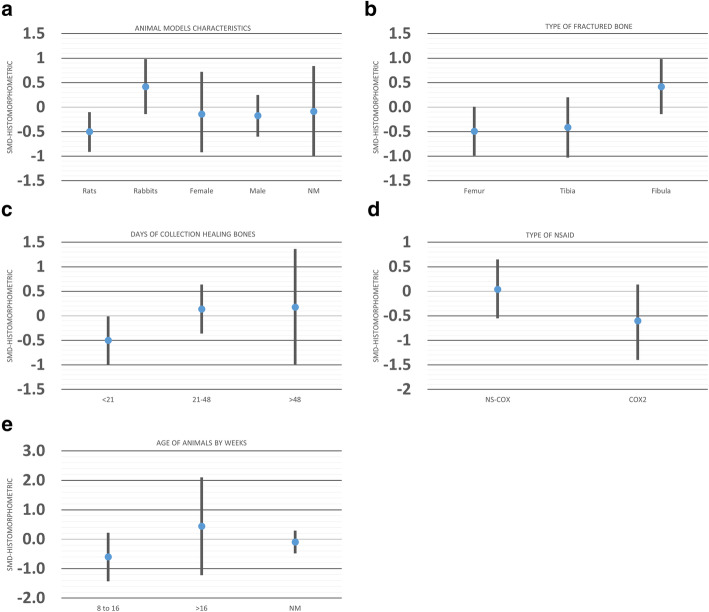
Effect of **a** animal models’ characteristics, **b** type of fractured bone, **c** time of collection healing bones, **d** type of NSAID, and **e** age of the animals on histomorphometric measurements (callus, bone) after administration of NSAID compared to the administration of a control vehicle. The columns indicate the effect estimate with the 95% confidence interval of the subgroups. SMD, standard mean difference–Hedges’ *g* mean. NM, not mentioned; NS-COX, non-selective-cyclooxygenase; COX2, selective-cyclooxygenase 2 inhibitor

Moreover, when comparing SMD across animal species, bone models, and time of collection, the effect of NSAID administration was significantly larger in rats compared to rabbits (*P* = 0.01; Fig. 10a), in femur compared to fibula (*P* = 0.02; Fig. 10b), and in the groups of bone samples that have been harvested less than 21 days (*P* = 0.03; Fig. 10c) after surgery.

### Publication bias

**Fig. 11:**
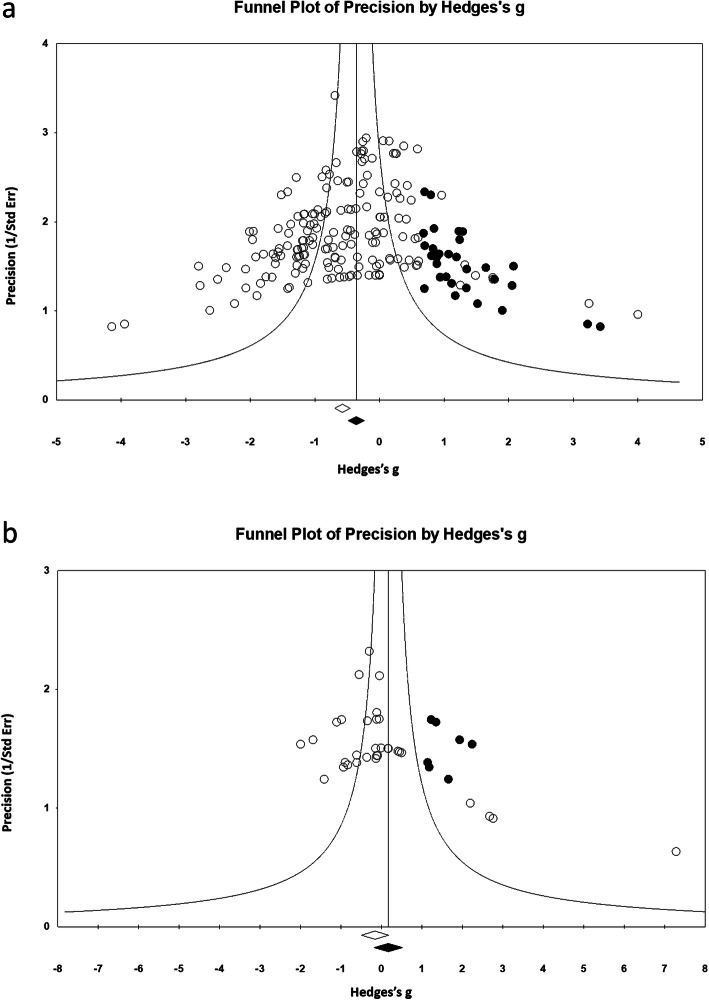
Funnel plot for **a** biomechanical and **b** histomorphometric measurements of bone in the included studies

The possible presence of publication bias was observed when assessing the biomechanical bending and histomorphometric outcome measurements. The inspection of the funnel plot suggested asymmetry resulting from the underrepresentation of studies regarding the effect of NSAID (Fig. 11 a, and b). For biomechanical outcome, trim and fill analysis resulted in data points, indicating the presence of publication bias (Duval and Tweedi’s trim and fill for adjusted values; point estimate = − 0.36; 95%CI − 0.48 to − 0.23; *Q* value 630.69). The Egger’s regression test intercept result (95% CI − 2.33 to − 0.67); *P* < 0.001. For the histomorphometric outcome; trim and fill analysis resulted in data points, indicating the presence of publication bias (Duval and Tweedi’s trim and fill for adjusted values; point estimate = − 0.248; 95%CI − 0.469 to − 0.027; *Q* value 70.55). The Egger’s regression test intercept result (95% CI 1.67 to 5.8); *P* < 0.001.

## Discussion

This unique systematic review and meta-analysis was designed to answer a specific research question regarding the effect of different types of NSAID on bone fracture healing in animal models (in vivo studies). Three important outcomes commonly used to assess bone healing were analyzed: biomechanical properties (maximum force to break, stiffness, and work to failure), micro CT, and histomorphometric measurements.

NSAID administration had a negative effect on the biomechanical properties in different animal models of the included studies [10, 12, 18, 19, 48, 51–59]. However, the results for histomorphometric assessments did not show a difference (Table 3).

Depending on the type of NSAID, they are known to inhibit both COX isoforms. Both non-selective NSAID and COX-2-selective drugs decrease prostaglandin production, which plays an essential regulatory role in all phases of bone healing, especially the inflammatory phase [60–63]. Thus, it is reasonable to expect that NSAID administration after bone fracture may delay or impair healing outcomes [12, 53, 62].

As early as the 1970s, many animal trials strongly emphasized the negative effect of NSAID on bone healing [7, 9, 12, 19, 24, 45, 53, 59, 64]. Most studies that used rodents and rabbits demonstrated that non-selective and selective COX inhibitors impair the bone healing process [7, 9–12, 19, 24, 45, 53, 59, 64]. Conversely, only a few studies indicated that NSAID has little or no effect on fracture healing outcomes [20, 56, 57, 65]. However, these studies have significant limitations, because they tested only one time point, did not measure clear bone healing outcomes that include mechanical or histomorphometric analysis, or used very low NSAID doses, and some did not perform a proper statistical analysis [43, 66, 67]. Recently, a review by Huss et al. in 2019 stated the need for more evidence based and weighting the risk and benefit regarding administration of NSAID for orthopedic treatment and the majority of animal experimental data support short term and perioperative administration of NSAID after bone fracture [68]. Clinically, there are few retrospective studies and even fewer prospective clinical trials [13–16]. The results from the retrospective studies were contradictory. Some of them have confirmed the negative effect of NSAID on bone formation and healing following hip or femoral neck fracture as well as hip arthroplasty [2, 13], while others have shown no effect on bone healing. One of the few prospective clinical trials reported beneficial effects of NSAID on bone healing in humans, which was among Colles’ fracture patients who were treated with casting and reduction [14, 16]. Borgeat et al. 2018, demonstrated in their systematic review that results from the available human trials did not show strong evidence that NSAID administration is related to increase non-union after bone fracture. In addition, they emphasised on the need for further randomized clinical trials to support or refuse this hypothesis [69]. It is widely accepted that trying to understand the effect of NSAID administration on bone healing is extremely challenging in a clinical setting especially from a methodological perspective. For example, controlling the many confounding factors (e.g., smoking, diabetes, obesity) in a prospective manner requires considerable time and planning [17]. Therefore, it is imperative to translate the evidence from the available in vivo experimental studies. In fact, animal studies helped in understanding the physiological process of bone healing and can also provide important insights on the effect of NSAID on bone healing.

Some methodological issues that might hamper the interpretation of the experimental animal data and their subsequent translation to the clinical setting should be discussed. First, there was substantial heterogeneity among the various animal studies. We performed subgroup analyses to investigate factors that may modify the effect of NSAID on bone healing outcomes (e.g., animal species, sex, type of bone, age of the animal, type of NSAID, and time at which the outcome was measured. More studies are required, especially in mice, because contrary to other models such as rats, mice show no negative effect of NSAID on the stiffness of the harvested bone compared to the control group. Because the mouse genetic map is similar to that of humans, the mouse may be the best available model to study the effect of NSAID on bone healing in different human genetic conditions [70]. Overall, the pharmacokinetic variations between species, sexes, and ages should be considered, especially regarding drug absorption. Our meta-analysis showed non-significant negative effect of NSAID administration after bone fracture not only in mice compared to other animals, but also in females compared to males, and in younger compared to older animals.

Our results should be interpreted with the limitation of the included studies regarding higher risk of bias, using healthy animal model and the sample size calculation. Experimental studies that compare different sexes and ages within the same experiment are needed for stratification and comparison.

With the above-mentioned limitations, we observed that the negative effect of NSAID administration on biomechanical properties differs between animal species and this may suggest that rodent models (mice and rats) may be more sensitive than others for bone healing outcomes. Additionally, this finding suggests that the negative effect of NSAID on bone healing is species related for certain outcomes, and this need to be taken into consideration for knowledge translation. Other factors should be taking into consideration in selecting animal model for experimental bone regeneration studies which are highlighted in the 2018 systemic review by Peric et al. such as skeletal features of the selected animals, the model that can mimic the clinical scenarios including relevant doses and statistically supported sample size [71]. The timing of healing outcome measurements also seems to modify the results because they do not show a significant effect of NSAID administration on μ-CT and histomorphometric outcomes early in the healing process compared to the control groups, but results differ when measurements are taken after 28 days. It is important to consider this information during the design of further experimental protocols, and it may be crucial in managing research efforts and reducing the unnecessary use of animals.

Finally, we did not find any studies that used animals of both sexes in their experimental design, which seems to be important for future studies because results differed between males and females for the work-to-failure biomechanical outcome.

### Methodological quality of the studies

Our study quality checklist assessed aspects of both internal and external validity, and we observed many studies were generally of low-quality scores and tended to overstate the effect size. The overall quality score accounted for a significant proportion of between-study heterogeneity; however, the correlation between the aggregate quality score and the effect was not clear. The reporting and risk of bias assessments indicate the need for protocol registration or publication, and for the reporting of the elements of randomization, blinding, and allocation concealment [35]. This concern is shared and addressed by others [33, 35, 38]. It is crucial that future animal studies improve the reporting of study procedures, allowing others to replicate and build on previously published work. With better reporting, systematic reviews of higher quality will also become feasible.

### Limitations

Several limitations of this work should be considered. One important limitation of this study was the risk of bias analysis which showed most of the included animal studies are poorly reported. This lack of reporting important methodological details might lead to increase bias. This limitation needs to be considered during interpretation of our conclusion from the included animal studies. In addition, we observed a level of heterogeneity present among studies reviewed for certain measured outcomes. This is can be expected as animal studies are explorative and heterogenous with respect species, sex, design, and drug administration protocol compared to clinical trials [35]. Exploring heterogeneity among animal studies is one of the objectives of performing meta-analysis of these studies that might help to inform future study design. The use of random-effects models as we did in this study can help to account for expecting heterogeneity [35]; however, appropriate caution still needs to be counted when interpreting the results. A fourth limitation is that the small sample size in animal studies which may exaggerate biases like publication bias which can affect the reliability and the validity of the study outcome. To overcome this limitation, one of the approaches we used was to calculate the standardized mean differences (SMD) through Hedges’ *g* effect sizes which is based on Cohen’s *D* but includes a correction factor for small sample size bias [35]. More specific limitation for this study was regarding the inclusion criteria of the systematic review, and we did not include animal models of disease or pathology, as we want to estimate the effect on healthy animal model, but this can be investigated and tested in future systematic review and meta-analysis with specific research question on the effect of NSAID administration on bone healing in diseased animal models. Other limitation is the meta-analysis of previous experimental studies did not include healing outcomes other than bone fracture (e.g., pain behavior or levels of inflammatory mediators). The measurements of pain behavior are subjective and depend on the interpretation of the examiner, which may lead to high heterogeneity among studies.

### Clinical implications

Drug pharmacokinetics vary between species, and this must be considered when extrapolating data from animals to humans. Therefore, it is important to investigate NSAID doses that are equivalent to those used in humans after bone fracture (through interspecies allometric scaling for dose conversion from animal to human studies) and to evaluate effects using different types of animal and bone fracture models. The increasing amount of evidence from animal studies and the results from this systematic review and meta-analysis indicate that caution should be exercised when using NSAID after bone fractures or with specific orthopedic surgical procedures until prospective human clinical studies indicate otherwise.

## Conclusions

Our findings provide some guidance for future laboratory and clinical research. First, it is important to test different hypotheses of bone fracture healing in small animals, and mice especially because mice models provide opportunities to examine genetics and create knock-out species. Our results also indicate the need for studies that compare the effect of NSAID administration on bone healing outcomes between male and female animal models. Overall, choosing appropriate animal model to test the effect of NSAID on fracture bone healing should take into consideration the animals’ species, type of the bone, and age of the animal.

Moreover, our results demonstrate it is important to choose the suitable time of sample collection based on what healing outcome to be measured. Histomorphometric outcome measurements require more than 21 days to show results that are comparable to the control group. Second, improvements in internal (study quality) and external (publication bias) validity might provide more information for the translation of the data to clinical trials, and more robust exploration of the efficacy limits in such studies could inform inclusion and exclusion criteria for these trials.

It is increasingly clear that the function of COX and their products are critical for bone healing. Most animal and human studies support the conclusion that NSAID administration can delay or impair bone fracture healing. However, the anti-inflammatory and analgesic effect of NSAID seems to be beneficial in treating post-traumatic pain and edema. The need for a new approach to using NSAID that preserves its pain control properties without affecting bone healing is justifiable and important.

## Supplementary Information

**Additional file 1: Table S1.** Systematic review and meta-analysis registered protocol in SYRCLE website: https://www.radboudumc.nl/en/research/technology-centers/animal-research-facility/systematic-review-center-for-laboratory-animal-experimentation/protocols NSAIDs effect on bone healing after bone fracture in animal models. **Table S2.** Search strategy. **Table S3.** SYRCLE’s tool for assessing the risk of bias (Hooijman et al 2014) (1). **Table S4.** Study characteristics. **Table S5.** Subgroup analysis by species regarding the effect of NSAID administration vs control on maximum force to fracture outcome. **Table S6.** Subgroup analysis by sex regarding the effect of NSAID administration vs control on maximum force to fracture outcome. **Table S7.** Subgroup analysis by age regarding the effect of NSAID administration vs control on maximum force to fracture outcome (1= <8 wks, 2=8-16wks, >16wks, 4=not mentioned). **Table S8.** Subgroup analysis by type pf NSAID regarding the effect of NSAID administration vs control on maximum force to fracture outcome. **Table S9.** Subgroup analysis by time point regarding the effect of NSAID administration vs control on maximum force to fracture outcome (1=<21days, 2=21-48days, 3=>48days). **Table S10.** Subgroup analysis by bone fracture site regarding the effect of NSAID administration vs control on maximum force to fracture outcome. **Table S11.** Subgroup analysis by species regarding the effect of NSAID administration vs control on stiffness to fracture outcome. **Table S12.** Subgroup analysis by sex regarding the effect of NSAID administration vs control on stiffness to fracture outcome. **Table S13.** Subgroup analysis by age regarding the effect of NSAID administration vs control on stiffness to fracture outcome (1= <8 wks, 2=8-16wks, >16wks, 4=not mentioned). **Table S14.** Subgroup analysis by type of NSAID regarding the effect of NSAID administration vs control on stiffness to fracture outcome. **Table S15.** Subgroup analysis by type of time point regarding the effect of NSAID administration vs control on stiffness to fracture outcome (1=<21days, 2=21-48days, 3=>48days). **Table S16.** Subgroup analysis by bone fracture site regarding the effect of NSAID administration vs control on stiffness to fracture outcome. **Table S17.** Subgroup analysis by species regarding the effect of NSAID administration vs control on work to failure outcome. **Table S18.** Subgroup analysis by sex regarding the effect of NSAID administration vs control on work to failure outcome. **Table S19.** Subgroup analysis by age regarding the effect of NSAID administration vs control on work to failure outcome (1= <8 wks, 2=8-16wks, >16wks, 4=not mentioned). **Table S20.** Subgroup analysis by type of NSAID regarding the effect of NSAID administration vs control on work to failure outcome. **Table S21.** Subgroup analysis by time point regarding the effect of NSAID administration vs control on work to failure outcome (1=<21days, 2=21-48days, 3=>48days). **Table S22.** Subgroup analysis by bone fracture site the effect of NSAID administration vs control on work to failure outcome. **Table S23.** Subgroup analysis by species the effect of NSAID administration vs control on histomorphometric outcome. **Table S24.** Subgroup analysis by sex the effect of NSAID administration vs control on histomorphometric outcome. **Table S25.** Subgroup analysis by age the effect of NSAID administration vs control on histomorphometric outcome (1= <8 wks, 2=8-16wks, >16wks, 4=not mentioned). **Table S26.** Subgroup analysis by type of NSAID the effect of NSAID administration vs control on histomorphometric outcome. **Table S27.** Subgroup analysis by time point the effect of NSAID administration vs control on histomorphometric outcome (1=<21days, 2=21-48days, 3=>48days). **Table S28.** Subgroup analysis by bone fracture site the effect of NSAID administration vs control on histomorphometric outcome.

**Additional file 2.**

**Additional file 3.**

## Data Availability

All data generated or analyzed during this study are included in this published article and its Supplementary files 1 and 2.

## Abbreviations

NSAID: Non-steroidal anti-inflammatory drugs
μ-CT: Micro-computed tomography
SYRCLE: SYstematic Review Center for Laboratory animal Experimentation
COX: Cyclooxygenase
SD: Standard deviation
SE: Standard error
SMD: Standardized mean differences
MF: Maximum force
