# Supplementary material for: Non-steroidal anti-inflammatory drugs and bone healing in animal models—a systematic review and meta-analysis
Source: Syst Rev. 2021 Jul 8;10:201. doi: 10.1186/s13643-021-01690-w (PMC8268344; doi:10.1186/s13643-021-01690-w)
Supplement: Supplementary file 1 — Additional file 1: Table S1. Systematic review and meta-analysis registered protocol in SYRCLE website: https://www.radboudumc.nl/en/research/technology-centers/animal-research-facility/systematic-review-center-for-laboratory-animal-experimentation/protocols NSAIDs effect on bone healing after bone fracture in animal models. Table S2. Search strategy. Table S3. SYRCLE’s tool for assessing the risk of bias (Hooijman et al 2014) (1). Table S4. Study characteristics. Table S5. Subgroup analysis by species regarding the effect of NSAID administration vs control on maximum force to fracture outcome. Table S6. Subgroup analysis by sex regarding the effect of NSAID administration vs control on maximum force to fracture outcome. Table S7. Subgroup analysis by age regarding the effect of NSAID administration vs control on maximum force to fracture outcome (1= <8 wks, 2=8-16wks, >16wks, 4=not mentioned). Table S8. Subgroup analysis by type pf NSAID regarding the effect of NSAID administration vs control on maximum force to fracture outcome. Table S9. Subgroup analysis by time point regarding the effect of NSAID administration vs control on maximum force to fracture outcome (1=<21days, 2=21-48days, 3=>48days). Table S10. Subgroup analysis by bone fracture site regarding the effect of NSAID administration vs control on maximum force to fracture outcome. Table S11. Subgroup analysis by species regarding the effect of NSAID administration vs control on stiffness to fracture outcome. Table S12. Subgroup analysis by sex regarding the effect of NSAID administration vs control on stiffness to fracture outcome. Table S13. Subgroup analysis by age regarding the effect of NSAID administration vs control on stiffness to fracture outcome (1= <8 wks, 2=8-16wks, >16wks, 4=not mentioned). Table S14. Subgroup analysis by type of NSAID regarding the effect of NSAID administration vs control on stiffness to fracture outcome. Table S15. Subgroup analysis by type of time point regarding the effec [file 13643_2021_1690_MOESM1_ESM.docx]

Supplementary information appendix

Table S1. Systematic review and meta-analysis registered protocol in SYRCLE website: https://www.radboudumc.nl/en/research/technology-centers/animal-research-facility/systematic-review-center-for-laboratory-animal-experimentation/protocols NSAIDs effect on bone healing after bone fracture in animal models.

| 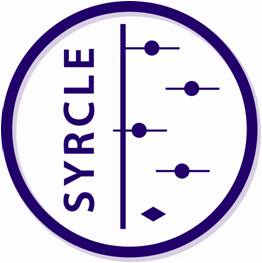 **Systematic Review Protocol for Animal Intervention Studies**  **Format by SYRCLE (**[**www.syrcle.nl**](http://www.syrcle.nl)**)**  **Version 2.0 (December 2014)** | | | | |
| --- | --- | --- | --- | --- |
| **Item #** | **Section/Subsection/Item** | **Description** | | **Check for approval** |
|  | A. General | | | |
| 1. | Title of the review | Non-Steroidal Anti-inflammatory Drugs and bone healing in animal Models - Systematic Review and Meta-Analysis | |  |
| 2. | Authors (names, affiliations, contributions) | Haider Al-Waeli: PhD Candidate, Faculty of Dentistry, McGill University, Canada. (writing manuscripts, reviewer of the first and full text screening, data extraction and meta-analysis)  Ana Paula Reboucas – MS Department of Pediatric Dentistry and Orthodontics, Faculty of Dentistry, Federal University of Minas Gerais, Belo Horizonte, Brazil. (reviewer of the first and full text screening, data extraction).  Martin Morris- Liaison Librarian, Schulich Library of Physical Sciences, McGill University, Canada. (search strategy, writing the methods and Prisma generation and optimizing search strategy)  Belinda Nicolau – Associate Professor at Faculty of Dentistry, McGill University, Canada. (Supervisor professor for the project, editing manuscripts) | |  |
| 3. | Other contributors (names, affiliations, contributions) | Alaa Mansour- PhD candidate, McGill University (second reviewer for the study quality extraction) | |  |
| 4. | Contact person + e-mail address | Haider Al-Waeli+haider.al-waeli@mail.mcgill.ca | |  |
| 5. | Funding sources/sponsors | None | |  |
| 6. | Conflicts of interest | None | |  |
| 7. | Date and location of protocol registration | N | |  |
| 8. | Registration number (if applicable) | N.A. | |  |
| 9. | Stage of review at time of registration | Full text screening | |  |
|  | B. Objectives | | | |
|  | Background | | | |
| 10. | What is already known about this disease/model/intervention? Why is it important to do this review? | Nonsteroidal anti-inflammatory is a widely prescribed drug for pain relief and inflammation in bone healing cases. Although some studies had associate its use to inhibition of fracture healing and to delay union bone. The effect of anti-inflammatory drugs administration in animal studies for bone healing is controversial, as some researches showed no effects using the drug. The aim of  this systematic review and meta -analysis is to assess the  outcomes correlated to nonsteroidal anti-inflammatory  therapy and bone healing in animal studies. | |  |
|  | Research question | | | |
| 11. | Specify the disease/health problem of interest | Bone fracture surgery | |  |
| 12. | Specify the population/species studied | Animal models | |  |
| 13. | Specify the intervention/exposure | Nonsteroidal anti-inflammatory agents | |  |
| 14. | Specify the control population | Use of placebo solution | |  |
| 15. | Specify the outcome measures | Bone biomechanical (primary Outcome), Bone volume or area (Histology grade or Micro CT) (second outcome) | |  |
| 16. | State your research question (based on items 11-15) | Does administration of NSAIDs after fracture bone surgery  resulted in lower bone morphometric and/or the  biomechanical outcome measurements in comparison to  control (placebo) administration in rodents animal model? | |  |
|  | C. Methods | | | |
|  | Search and study identification | | | |
| 17. | Identify literature databases to search (*e.g.* Pubmed, Embase, Web of science) | - MEDLINE via PubMed - SCOPUS - EMBASE - Ovid | |  |
| 18. | Define electronic search strategies (*e.g.* use the [step by step search guide^15^](http://www.ncbi.nlm.nih.gov/pmc/articles/PMC3265183/pdf/LA-11-087.pdf) and animal search filters[^20,^](http://www.ncbi.nlm.nih.gov/pmc/articles/PMC3104815/pdf/LA-09-117.pdf) [^21^](http://lan.sagepub.com/content/48/1/88.full.pdf+html)) | When available, please add a supplementary file containing your search strategy: [Search Strategy Appendix] | |  |
| 19. | Identify other sources for study identification | □Reference lists of included studies □Books  □Reference lists of relevant reviews  □Conference proceedings, namely:  □Contacting authors/ organisations, namely:  □Other, namely: | |  |
| 20. | Define search strategy for these other sources |  | |  |
|  | Study selection | | | |
| 21. | Define screening phases (*e.g.* pre-screening based on title/abstract, full text screening, both) | - Initial pre-screening with selection of the relevant studies based on the key components of the review question on title/abstract  - Full text screening of the relevant citations | |  |
| 22. | Specify (a) the number of reviewers per screening phase and (b) how discrepancies will be resolved | - Pre-screening and full text screening will be performed by two reviewers independently  - Discrepancies will be solved either by discussion or by  a third reviewer (when no agreement is met by the two  reviewers) | |  |
|  | *Define all inclusion and exclusion criteria based on:* | | | |
| 23. | Type of study (design) | Inclusion criteria:   - Original articles - Experimental animal models - In vivo (designed for bone healing process)   Exclusion criteria:   - Not an original study - In vitro studies - Clinical trials - Case reports - Review studies | |  |
| 24. | Type of animals/population (*e.g.* age, gender, disease model) | Inclusion criteria:   - Animal models - Bone fracture models   Exclusion criteria:   - Clinical trials | |  |
| 25. | Type of intervention (*e.g.* dosage, timing, frequency) | Inclusion criteria:   - Nonsteroidal anti-inflammatory agents in any dose, duration, frequency and type.   Exclusion criteria:   - Studies taking another drug intervention rather than   nonsteroidal anti-inflammatory agents:   - Steroidal anti-inflammatory agents - Antibiotics - Combination of NSAIDs and other interventions | |  |
| 26. | Outcome measures | Inclusion criteria:   - Bone biomechanical test/maximum force - Histological analysis/ Hu etal healing grade - Micro CT /bone volume or area   Exclusion criteria:  Studies assessing healing in other tissues rather than bone  tissue | |  |
| 27. | Language restrictions |  | |  |
| 28. | Publication date restrictions | Inclusion criteria:   - No date restriction   Exclusion criteria:   - N/A | |  |
| 29. | Other | Inclusion criteria:   - N/A   Exclusion criteria:   - N/A | |  |
| 30. | Sort and prioritize your exclusion criteria per selection phase | Selection phase: Screening title/abstract and Full text  1. No bone fracture model  2. No use of nonsteroidal anti-inflammatory agents  3. No animal study  4. Assessment of healing in others tissues not bone  5. No primary study or review | |  |
|  | Study characteristics to be extracted (for assessment of external validity, reporting quality) | | | |
| 31. | Study ID (*e.g.* authors, year) | ‐ Authors,  ‐ Year,  ‐ Title,  ‐ Journal,  ‐ Language | |  |
| 32. | Study design characteristics (*e.g.* experimental groups, number of animals) | number of groups  number of animals per group (total and per test)  Non selective COX NSAIDs group/s, Selective COX2 group/s, Control group/s  number of excluded, reason for exclusion. | |  |
| 33. | Animal model characteristics (*e.g.* species, gender, disease induction) | Animal type, weight, age and sex, type of the bone  fracture model. | |  |
| 34. | Intervention characteristics (*e.g.* intervention, timing, duration) | Time of administration, duration, dose mg/kg/day,route of administration. | |  |
| 35. | Outcome measures | Maximum force of torque of the mechanical bending (N.mm)  Bone area  Healing grade of histology examination | |  |
| 36. | Other (*e.g.* drop-outs) | Reason for drop out of the study | |  |
|  | Assessment risk of bias (internal validity) or study quality | | | |
| 37. | Specify (a) the number of reviewers assessing the risk of bias/study quality in each study and (b) how discrepancies will be resolved | - Two independent reviewers  - Discrepancies or disagreements will be resolved after discussion with a third reviewer | |  |
| 38. | Define criteria to assess (a) the internal validity of included studies (*e.g.* selection, performance, detection and attrition bias) and/or (b) other study quality measures (*e.g.* reporting quality, power) | □By use of [SYRCLE's Risk of Bias tool^4^](http://www.biomedcentral.com/1471-2288/14/43/abstract)  □By use of SYRCLE’s Risk of Bias tool, adapted as follows:  □By use of [CAMARADES' study quality checklist, e.g ^22^](http://www.ncbi.nlm.nih.gov/pubmed/15060322)  □By use of CAMARADES' study quality checklist, adapted as follows: Select items that are related to the study design  □Other criteria, namely: | |  |
|  | Collection of outcome data | | | |
| 39. | For each outcome measure, define the type of data to be extracted (*e.g.* continuous/dichotomous, unit of measurement) | In general, the data will be continuous (generally measured in force for the maximum force of mechanical bending), Units of measurement tend to be N.mm  For the Histological healing data will be score of grade,bone volume or area will be measured by mm^2^, or mm^3.^ | |  |
| 40. | Methods for data extraction/retrieval (*e.g.* first extraction from graphs using a digital screen ruler, then contacting authors) | 1. Extract data from text/tables Two reviewrs(HA,AM)  3. Contact authors for missing data | |  |
| 41. | Specify (a) the number of reviewers extracting data and (b) how discrepancies will be resolved | One reviewer (HA) will extract data. A second reviewer (AM) will check the extraction process. | |  |
|  | Data analysis/synthesis | | | |
| 42. | Specify (per outcome measure) how you are planning to combine/compare the data (*e.g.* descriptive summary, meta-analysis) | Maximum force measurements will be recorded from each study, sub group analysis will be done regarding type of NSAIDs, rodent model, and time. For grade of healing or bone volume and area will be recorded for meta analysis if not available then descriptive summary will be mentioned for every outcome. | |  |
| 43. | Specify (per outcome measure) how it will be decided whether a meta-analysis will be performed | If at least 5 studies are found per outcome, data will be pooled for the meta-analysis, high heterogeneity will be  investigated to check the refrain form the meta-analysis. | |  |
|  | *If a meta-analysis seems feasible/sensible, specify (for each outcome measure):* | | | |
| 44. | The effect measure to be used (*e.g.* mean difference, standardized mean difference, risk ratio, odds ratio) | We will use mean differences if studies use the same experimental test with the same scoring scale, but standardized mean difference if combining different scale score for the same measurement. | |  |
| 45. | The statistical model of analysis (*e.g.* random or fixed effects model) | Random effect model | |  |
| 46. | The statistical methods to assess heterogeneity (*e.g.* I^2^, Q) | I^2^ | |  |
| 47. | Which study characteristics will be examined as potential source of heterogeneity (subgroup analysis) | Type of NSAIDs, Timing, animal species, time of observation, | |  |
| 48. | Any sensitivity analyses you propose to perform | We will perform sensitivity analyses to assess if our underlying assumptions are appropriate and our results are robust. | |  |
| 49. | Other details meta-analysis (*e.g.* correction for multiple testing, correction for multiple use of control group) | We will perform a Holm-Bonferroni correction to correct for multiple testing. We will adjust the p value according to the number of the subgroup analysis. If within one study several doses of NSAIDs are compared to one control group, we will divide the number of control animals by the total number of comparisons made with this group in order to correct for repeated use of one control group. | |  |
| 50. | The method for assessment of publication bias | Funnel plot (if at least 10 studies included in meta-analysis) | |  |
|  | | | | |
| Final approval by (names, affiliations): | |  | Date: | |

Table S 2. Search strategy

| # | | Search Statement |
| --- | --- | --- |
| Medline data base (start from January 1^st^ 1946) | | |
| 1 | | exp Anti-Inflammatory Agents, Non-Steroidal/ |
| 2 | | (non-steroid* adj3 (inflammat* or anti-inflammat* or antiinflammat*)).ti,ab,kf. |
| 3 | | ((cyclooxygenase or cyclo-oxygenase or COX-2) adj1 (inhibit* or attenuat*)).ti,ab,kf. |
| 4 | | (adapalene or ampyrone or antipyrine or apazone or aspirin or bufexamac or carprofen or celecoxib or clonixin or curcumin or diclofenac or diflunisal or dipyrone or epirizole or etanercept or etodolac or fenoprofen or feprazone or flurbiprofen or ibuprofen or indomethacin or ketoprofen or ketorolac or masoprocol or meclofenamic or mefenamic or mesalamine or naproxen or niflumic or olopatadine or oxyphenbutazone or phenylbutazone or piroxicam or salicylate* or sulfasalazine or sulindac or suprofen or tolmetin).ti,ab,kf. |
| 5 | | (NSAID? or tactupump or galderma or differin or auralgan or paladin or salicylic or floctafenine or meloxicam or nabumetone or napafenac or oxaprozin or tenoxicam or tiaprofenic).ti,ab,kf. |
| 6 | | or/1-5 |
| 7 | | exp Fractures, Bone/ |
| 8 | | exp "Bone and Bones"/de, in, me, pa, pd, pp [Drug Effects, Injuries, Metabolism, Pathology, Pharmacology, Physiopathology] |
| 9 | | exp Fracture Healing/ |
| 10 | | exp Bone Remodeling/ |
| 11 | | exp Bone Density/ |
| 12 | | (bone adj3 (broken or fracture* or remodel* or regenerat* or density)).ti,ab,kf. |
| 13 | | ((fracture? or bone?) adj3 heal*).ti,ab,kf. |
| 14 | | ((displace* or open or closed or comminuted or greenstick or transverse or oblique or buckled or pathologic or stress or hairline or compound or traumatic or periprosthetic or linear or spiral or compression or avulsion or impacted or burst or chance or flexion or supracondylar) adj3 fracture*).ti,ab,kf. |
| 15 | | ((holstein-lewis or jefferson or clay-shoveler or holdsworth or hume or essex-lopresti or galeazzi or colles? or smith* or barton* or monteggia or rolando or bennett* or boxer* or duverney or pilon or bumper or segond or gosselin or toddler* or bosworth or maisonneuve or "le fort" or pott* or lisfranc or jones or march) adj3 fracture*).ti,ab,kf. |
| 16 | | ((skull or mandibular or nasal or cervical or rib? or sternal or shoulder? or arm? or humerus or forearm? or ulnar or radius or distal or scaphoid or pelvic or femoral or patella or crus or tibia? or trimalleolar or calcaneal or bimalleolar) adj3 fracture*).ti,ab,kf. |
| 17 | | or/7-16 |
| 18 | | 6 and 17 |
| 19 | | 18 not (humans/ not (humans/ and animals/)) |
| COCHRANE LIBRARY/DARE/CENTRAL ( started from  (All keyword searches are "All Text") | | |
| 1 | | MeSH descriptor: [Anti-Inflammatory Agents, Non-Steroidal] explode all trees |
| 2 | | (non-steroid* NEAR/3 (inflammat* or anti-inflammat* or antiinflammat*)) |
| 3 | | ((cyclooxygenase or cyclo-oxygenase or COX-2) NEAR/1 (inhibit* or attenuat*)) |
| 4 | | (adapalene or ampyrone or antipyrine or apazone or aspirin or bufexamac or carprofen or celecoxib or clonixin or curcumin or diclofenac or diflunisal or dipyrone or epirizole or etanercept or etodolac or fenoprofen or feprazone or flurbiprofen or ibuprofen or indomethacin or ketoprofen or ketorolac or masoprocol or meclofenamic or mefenamic or mesalamine or naproxen or niflumic or olopatadine or oxyphenbutazone or phenylbutazone or piroxicam or salicylate* or sulfasalazine or sulindac or suprofen or tolmetin) |
| 5 | | (NSAID? or tactupump or galderma or differin or auralgan or paladin or salicylic or floctafenine or meloxicam or nabumetone or napafenac or oxaprozin or tenoxicam or tiaprofenic) |
| 6 | | #1 OR #2 OR #3 OR #4 OR #5 |
| 7 | | MeSH descriptor: [Fractures, Bone] explode all trees |
| 8 | | MeSH descriptor: [Bone and Bones] explode all trees |
| 9 | | MeSH descriptor: [Fracture Healing] explode all trees |
| 10 | | MeSH descriptor: [Bone Remodeling] explode all trees |
| 11 | | MeSH descriptor: [Bone Density] explode all trees |
| 12 | | (bone NEAR/3 (broken or fracture* or remodel* or regenerat* or density)) |
| 13 | | ((fracture? or bone?) NEAR/3 heal*) |
| 14 | | ((displace* or open or closed or comminuted or greenstick or transverse or oblique or buckled or pathologic or stress or hairline or compound or traumatic or periprosthetic or linear or spiral or compression or avulsion or impacted or burst or chance or flexion or supracondylar) NEAR/3 fracture*) |
| 15 | | ((holstein-lewis or jefferson or clay-shoveler or holdsworth or hume or essex-lopresti or galeazzi or colles? or smith* or barton* or monteggia or rolando or bennett* or boxer* or duverney or pilon or bumper or segond or gosselin or toddler* or bosworth or maisonneuve or "le fort" or pott* or lisfranc or jones or march) NEAR/3 fracture*) |
| 16 | | ((skull or mandibular or nasal or cervical or rib? or sternal or shoulder? or arm? or humerus or forearm? or ulnar or radius or distal or scaphoid or pelvic or femoral or patella or crus or tibia? or trimalleolar or calcaneal or bimalleolar) NEAR/3 fracture*) |
| 17 | | #7 OR #8 OR #9 OR #10 OR #11 OR #12 OR #13 OR #14 OR #15 OR #16 |
| 18 | | #6 AND #17 |
| EMBASE (OVID) | | |
| 1 | | exp nonsteroid antiinflammatory agent/ |
| 2 | | exp prostaglandin synthase inhibitor/ |
| 3 | | (non-steroid* adj3 (inflammat* or anti-inflammat* or antiinflammat*)).ti,ab,kw. |
| 4 | | (adapalene or ampyrone or antipyrine or apazone or aspirin or bufexamac or carprofen or celecoxib or clonixin or curcumin or diclofenac or diflunisal or dipyrone or epirizole or etanercept or etodolac or fenoprofen or feprazone or flurbiprofen or ibuprofen or indomethacin or ketoprofen or ketorolac or masoprocol or meclofenamic or mefenamic or mesalamine or naproxen or niflumic or olopatadine or oxyphenbutazone or phenylbutazone or piroxicam or salicylate* or sulfasalazine or sulindac or suprofen or tolmetin).ti,ab,kw. |
| 5 | | (NSAID? or tactupump or galderma or differin or auralgan or paladin or salicylic or floctafenine or meloxicam or nabumetone or napafenac or oxaprozin or tenoxicam or tiaprofenic).ti,ab,kw. |
| 6 | | ((cyclooxygenase or cyclo-oxygenase or COX-2) adj1 (inhibit* or attenuat*)).ti,ab,kw. |
| 7 | | or/1-6 |
| 8 | | exp *fracture/ |
| 9 | | exp *bone/ |
| 10 | | exp bone remodeling/ |
| 11 | | exp bone density/ |
| 12 | | (bone adj3 (broken or fracture* or remodel* or regenerat* or density)).ti,ab,kw. |
| 13 | | ((fracture? or bone?) adj3 heal*).ti,ab,kw. |
| 14 | | ((displace* or open or closed or comminuted or greenstick or transverse or oblique or buckled or pathologic or stress or hairline or compound or traumatic or periprosthetic or linear or spiral or compression or avulsion or impacted or burst or chance or flexion or supracondylar) adj3 fracture*).ti,ab,kw. |
| 15 | | ((holstein-lewis or jefferson or clay-shoveler or holdsworth or hume or essex-lopresti or galeazzi or colles? or smith* or barton* or monteggia or rolando or bennett* or boxer* or duverney or pilon or bumper or segond or gosselin or toddler* or bosworth or maisonneuve or "le fort" or pott* or lisfranc or jones or march) adj3 fracture*).ti,ab,kw. |
| 16 | | ((skull or mandibular or nasal or cervical or rib? or sternal or shoulder? or arm? or humerus or forearm? or ulnar or radius or distal or scaphoid or pelvic or femoral or patella or crus or tibia? or trimalleolar or calcaneal or bimalleolar) adj3 fracture*).ti,ab,kw. |
| 17 | | or/8-16 |
| 18 | | 7 and 17 |
| 19 | | 18 not (humans/ not (humans/ and animals/)) |
| CINAHL | | |
| 1 | | (MH "Antiinflammatory Agents, Non-Steroidal+") |
| 2 | | TI (non-steroid* N3 (inflammat* or anti-inflammat* or antiinflammat*)) OR AB (non-steroid* N3 (inflammat* or anti-inflammat* or antiinflammat*)) |
| 3 | | TI ((cyclooxygenase or cyclo-oxygenase or COX-2) N1 (inhibit* or attenuat*)) OR AB ((cyclooxygenase or cyclo-oxygenase or COX-2) N1 (inhibit* or attenuat*)) |
| 4 | | TI (adapalene or ampyrone or antipyrine or apazone or aspirin or bufexamac or carprofen or celecoxib or clonixin or curcumin or diclofenac or diflunisal or dipyrone or epirizole or etanercept or etodolac or fenoprofen or feprazone or flurbiprofen or ibuprofen or indomethacin or ketoprofen or ketorolac or masoprocol or meclofenamic or mefenamic or mesalamine or naproxen or niflumic or olopatadine or oxyphenbutazone or phenylbutazone or piroxicam or salicylate* or sulfasalazine or sulindac or suprofen or tolmetin) OR AB (adapalene or ampyrone or antipyrine or apazone or aspirin or bufexamac or carprofen or celecoxib or clonixin or curcumin or diclofenac or diflunisal or dipyrone or epirizole or etanercept or etodolac or fenoprofen or feprazone or flurbiprofen or ibuprofen or indomethacin or ketoprofen or ketorolac or masoprocol or meclofenamic or mefenamic or mesalamine or naproxen or niflumic or olopatadine or oxyphenbutazone or phenylbutazone or piroxicam or salicylate* or sulfasalazine or sulindac or suprofen or tolmetin) |
| 5 | | TI (NSAID? or tactupump or galderma or differin or auralgan or paladin or salicylic or floctafenine or meloxicam or nabumetone or napafenac or oxaprozin or tenoxicam or tiaprofenic) OR AB (NSAID? or tactupump or galderma or differin or auralgan or paladin or salicylic or floctafenine or meloxicam or nabumetone or napafenac or oxaprozin or tenoxicam or tiaprofenic) |
| 6 | | S1 OR S2 OR S3 OR S4 OR S5 |
| 7 | | (MH "Fractures+") |
| 8 | | (MH "Bone and Bones+/DE/IN/ME/PA/PP") |
| 9 | | (MH "Fracture Healing") |
| 10 | | (MH "Bone Remodeling+") |
| 11 | | (MH "Bone Density") |
| 12 | | TI (bone N3 (broken or fracture* or remodel* or regenerat* or density)) OR AB (bone N3 (broken or fracture* or remodel* or regenerat* or density)) |
| 13 | | TI ((fracture? or bone?) N3 heal*) OR AB ((fracture? or bone?) N3 heal*) |
| 14 | | TI ((displace* or open or closed or comminuted or greenstick or transverse or oblique or buckled or pathologic or stress or hairline or compound or traumatic or periprosthetic or linear or spiral or compression or avulsion or impacted or burst or chance or flexion or supracondylar) N3 fracture*) OR AB ((displace* or open or closed or comminuted or greenstick or transverse or oblique or buckled or pathologic or stress or hairline or compound or traumatic or periprosthetic or linear or spiral or compression or avulsion or impacted or burst or chance or flexion or supracondylar) N3 fracture*) |
| 15 | | TI ((holstein-lewis or jefferson or clay-shoveler or holdsworth or hume or essex-lopresti or galeazzi or colles? or smith* or barton* or monteggia or rolando or bennett* or boxer* or duverney or pilon or bumper or segond or gosselin or toddler* or bosworth or maisonneuve or "le fort" or pott* or lisfranc or jones or march) N3 fracture*) OR AB ((holstein-lewis or jefferson or clay-shoveler or holdsworth or hume or essex-lopresti or galeazzi or colles? or smith* or barton* or monteggia or rolando or bennett* or boxer* or duverney or pilon or bumper or segond or gosselin or toddler* or bosworth or maisonneuve or "le fort" or pott* or lisfranc or jones or march) N3 fracture*) |
| 16 | | TI ((skull or mandibular or nasal or cervical or rib? or sternal or shoulder? or arm? or humerus or forearm? or ulnar or radius or distal or scaphoid or pelvic or femoral or patella or crus or tibia? or trimalleolar or calcaneal or bimalleolar) N3 fracture*) OR AB ((skull or mandibular or nasal or cervical or rib? or sternal or shoulder? or arm? or humerus or forearm? or ulnar or radius or distal or scaphoid or pelvic or femoral or patella or crus or tibia? or trimalleolar or calcaneal or bimalleolar) N3 fracture*) |
| 17 | | S7 OR S8 OR S9 OR S10 OR S11 OR S12 OR S13 OR S14 OR S15 OR S16 |
| 18 | | S6 AND S17 |
| Scopus | | |
| ( INDEXTERMS ( "Antiinflammatory Agents, Non-Steroidal" )  OR  TITLE-ABS-KEY ( non-steroid*  W/3  ( inflammat*  OR  anti-inflammat*  OR  antiinflammat* ) )  OR  TITLE-ABS-KEY ( ( cyclooxygenase  OR  cyclo-oxygenase  OR  cox-2 )  W/1  ( inhibit*  OR  attenuat* ) )  OR  TITLE-ABS-KEY ( adapalene  OR  ampyrone  OR  antipyrine  OR  apazone  OR  aspirin  OR  bufexamac  OR  carprofen  OR  celecoxib  OR  clonixin  OR  curcumin  OR  diclofenac  OR  diflunisal  OR  dipyrone  OR  epirizole  OR  etanercept  OR  etodolac  OR  fenoprofen  OR  feprazone  OR  flurbiprofen  OR  ibuprofen  OR  indomethacin  OR  ketoprofen  OR  ketorolac  OR  masoprocol  OR  meclofenamic  OR  mefenamic  OR  mesalamine  OR  naproxen  OR  niflumic  OR  olopatadine  OR  oxyphenbutazone  OR  phenylbutazone  OR  piroxicam  OR  salicylate*  OR  sulfasalazine  OR  sulindac  OR  suprofen  OR  tolmetin )  OR  TITLE-ABS-KEY ( nsaid?  OR  tactupump  OR  galderma  OR  differin  OR  auralgan  OR  paladin  OR  salicylic  OR  floctafenine  OR  meloxicam  OR  nabumetone  OR  napafenac  OR  oxaprozin  OR  tenoxicam  OR  tiaprofenic ) )  AND  ( INDEXTERMS ( "Fractures, Bone" )  OR  INDEXTERMS ( "Bone and Bones" )  OR  INDEXTERMS ( "Fracture Healing" )  OR  INDEXTERMS ( "Bone Remodeling" )  OR  INDEXTERMS ( "Bone Density" )  OR  TITLE-ABS-KEY ( bone  W/3  ( broken  OR  fracture*  OR  remodel*  OR  regenerat*  OR  density ) )  OR  TITLE-ABS-KEY ( ( fracture?  OR  bone? )  W/3  heal* )  OR  TITLE-ABS-KEY ( ( displace*  OR  open  OR  closed  OR  comminuted  OR  greenstick  OR  transverse  OR  oblique  OR  buckled  OR  pathologic  OR  stress  OR  hairline  OR  compound  OR  traumatic  OR  periprosthetic  OR  linear  OR  spiral  OR  compression  OR  avulsion  OR  impacted  OR  burst  OR  chance  OR  flexion  OR  supracondylar )  W/3  fracture* )  OR  TITLE-ABS-KEY ( ( holstein-lewis  OR  jefferson  OR  clay-shoveler  OR  holdsworth  OR  hume  OR  essex-lopresti  OR  galeazzi  OR  colles?  OR  smith*  OR  barton*  OR  monteggia  OR  rolando  OR  bennett*  OR  boxer*  OR  duverney  OR  pilon  OR  bumper  OR  segond  OR  gosselin  OR  toddler*  OR  bosworth  OR  maisonneuve  OR  "le fort"  OR  pott*  OR  lisfranc  OR  jones  OR  march )  W/3  fracture* )  OR  TITLE-ABS-KEY ( ( skull  OR  mandibular  OR  nasal  OR  cervical  OR  rib?  OR  sternal  OR  shoulder?  OR  arm?  OR  humerus  OR  forearm?  OR  ulnar  OR  radius  OR  distal  OR  scaphoid  OR  pelvic  OR  femoral  OR  patella  OR  crus  OR  tibia?  OR  trimalleolar  OR  calcaneal  OR  bimalleolar )  W/3  fracture* ) )  AND NOT  DBCOLL ( medl ) | | |
| Biosis (Ovid) | | |
| 1 | (non-steroid* adj3 (inflammat* or anti-inflammat* or antiinflammat*)).ti,ab,kf. | |
| 2 | ((cyclooxygenase or cyclo-oxygenase or COX-2) adj1 (inhibit* or attenuat*)).ti,ab,kf. | |
| 3 | (adapalene or ampyrone or antipyrine or apazone or aspirin or bufexamac or carprofen or celecoxib or clonixin or curcumin or diclofenac or diflunisal or dipyrone or epirizole or etanercept or etodolac or fenoprofen or feprazone or flurbiprofen or ibuprofen or indomethacin or ketoprofen or ketorolac or masoprocol or meclofenamic or mefenamic or mesalamine or naproxen or niflumic or olopatadine or oxyphenbutazone or phenylbutazone or piroxicam or salicylate* or sulfasalazine or sulindac or suprofen or tolmetin).ti,ab,kf. | |
| 4 | (NSAID? or tactupump or galderma or differin or auralgan or paladin or salicylic or floctafenine or meloxicam or nabumetone or napafenac or oxaprozin or tenoxicam or tiaprofenic).ti,ab,kf. | |
| 5 | or/1-5 | |
| 6 | (bone adj3 (broken or fracture* or remodel* or regenerat* or density)).ti,ab,kf. | |
| 7 | ((fracture? or bone?) adj3 heal*).ti,ab,kf. | |
| 8 | ((displace* or open or closed or comminuted or greenstick or transverse or oblique or buckled or pathologic or stress or hairline or compound or traumatic or periprosthetic or linear or spiral or compression or avulsion or impacted or burst or chance or flexion or supracondylar) adj3 fracture*).ti,ab,kf. | |
| 9 | ((holstein-lewis or jefferson or clay-shoveler or holdsworth or hume or essex-lopresti or galeazzi or colles? or smith* or barton* or monteggia or rolando or bennett* or boxer* or duverney or pilon or bumper or segond or gosselin or toddler* or bosworth or maisonneuve or "le fort" or pott* or lisfranc or jones or march) adj3 fracture*).ti,ab,kf. | |
| 10 | ((skull or mandibular or nasal or cervical or rib? or sternal or shoulder? or arm? or humerus or forearm? or ulnar or radius or distal or scaphoid or pelvic or femoral or patella or crus or tibia? or trimalleolar or calcaneal or bimalleolar) adj3 fracture*).ti,ab,kf. | |
| 11 | or/7-16 | |
| 12 | 5 and 11 | |

Table S 3. SYRCLE’s tool for assessing the risk of bias (Hooijman et al 2014) (1)

| Item | Type of bias | Domain | Description of domain | Review authors judgment |
| --- | --- | --- | --- | --- |
| 1 | Selection bias | Sequence generation | Describe the methods used, if any, to generate the allocation sequence in sufficient detail to allow an assessment whether it should produce comparable groups. | Was the allocation sequence adequately generated and applied? (*) |
| 2 | Selection bias | Baseline characteristics | Describe all the possible prognostic factors or animal characteristics, if any, that are compared in order to judge whether or not intervention and control groups were similar at the start of the experiment. | Were the groups similar at baseline or were they adjusted for confounders in the analysis? |
| 3 | Selection bias | Allocation concealment | Describe the method used to conceal the allocation sequence in sufficient detail to determine whether intervention allocations could have been foreseen before or during enrolment. | Was the allocation adequately concealed? (*) |
| 4 | Performance bias | Random housing | Describe all measures used, if any, to house the animals randomly within the animal room. | Were the animals randomly housed during the experiment? |
| 5 | Performance bias | Blinding | Describe all measures used, if any, to blind trial caregivers and researchers from knowing which intervention each animal received. Provide any information relating to whether the intended blinding was effective. | Were the caregivers and/or investigators blinded from knowledge which intervention each animal received during the experiment? |
| 6 | Detection bias | Random outcome assessment | Describe whether or not animals were selected at random for outcome assessment, and which methods to select the animals, if any, were used. | Were animals selected at random for outcome assessment? |
| 7 | Detection bias | Blinding | Describe all measures used, if any, to blind outcome assessors from knowing which intervention each animal received. Provide any information relating to whether the intended blinding was effective. | Was the outcome assessor blinded? |
| 8 | Attrition bias | Incomplete outcome data | Describe the completeness of outcome data for each main outcome, including attrition and exclusions from the analysis. State whether attrition and exclusions were reported, the numbers in each intervention group (compared with total randomized animals), reasons for attrition or exclusions, and any re-inclusions in analyses for the review. | Were incomplete outcome data adequately addressed? (*) |
| 9 | Reporting bias | Selective outcome reporting | State how selective outcome reporting was examined and what was found. | Are reports of the study free of selective outcome reporting? (*) |
| 10 | Other | Other sources of bias | State any important concerns about bias not covered by other domains in the tool. | Was the study apparently free of other problems that could result in high risk of bias? (*) |

*Items in agreement with the items in the Cochrane Risk of Bias tool.

Table S 4. Study characteristics

| Study | Year | Species | Age | Weight | Sex | Type of bone | Type of NSAID | Dose | Duration | Primary outcome measures | Time point | Other measurement | Time  point |
| --- | --- | --- | --- | --- | --- | --- | --- | --- | --- | --- | --- | --- | --- |
| Akman et al(2) | 2002 | Rats | 20 | 270 | M | Tibia | NS-COX(Dicl.) | 1,2 | 10 | NO | NA | Clin, Radiog, Hist-scores(Huo) | 14,28.42 |
| Allen et al(3) | 1980 | Rats | 6 | NA | M | Other | NS-COX(Ind.,Asp.) | 2,4,100,200,300 | 21 | NO | NA | Hist-Scores | 21 |
| Altman et al(4) | 1995 | Rats | retired | 375 | F | Femur | NS-COX(ind.,ibu.) | 1,30 | 4,12 | 3PMB-FR | 14,28,48,57,84 | Hist-Scores(Huo) | 14,28,48,57,84 |
| Beck et al(5) | 2003 | Rats | NA | 325 | M | Tibia | NS-COX(Dicl.) | 5 | 7,21 | 3PMB-F,3PMB-Stiff | 21 | BD-µ-CT | 21 |
| Bergenstock et al (6) | 2005 | Rats | NA | 301 | F | Femur | COX-2(Celec.) | 3,6,60,300 | 10 | 3PMB-F,3PMB-Stiff | 56 | Radiog.(scores),  Non-union ratio, Hist. | 56 |
| Brown et al(7) | 2004 | Rats | NA | 300 | M | Femur | NS-COX(Ind.),  COX-2(Celec.) | 1,3 | 28,56,84 | 3PMB-F,3PMB-Stiff | 28,56,84 | Radiog, Hist-grades | 28,56,84 |
| Cappello et al(8) | 2013 | Rats | 3 | NA | M | Tibia | NS-COX(Ket.) | 5 | 7,14,21 | 4PMB-F | 21 | Hist-description | 7,14,21 |
| Dimmen et al(9) | 2008 | Rats | adult | 213 | F | Tibia | COX-2(Par.) | 10 | 7 | 3PMB-F,3PMB-Stiff | 6 | DEXA-BD | 14,21,42 |
| Dimmen et al(10) | 2009 | Rats | NA | 226.5 | F | Tibia | NS-COX(Ind.),COX-2(Par.) | 1.625,1 | 7 | 3PMB-F,3PMB-Stiff | 21 | DEXA-BD | 14,21 |
| Lack et al(11) | 2013 | Rabbit | NA | NA | NA | Ulna | NS-COX(Ind.,Aspirin) | 10, 10 | 56 | 3PMB-F | 56 | Radiog.,Hist. | 14,28,42,56 |
| More et al(12) | 1989 | Rabbit | Adolescent | 260 | F | Tibia | NS-COX(Flunixin,Piroxicam) | 1.1,0.2 | 21 | 3PMB-F | 21 | Ankle stiffness | Not clear |
| Endo et al(13) | 2005 | Rats | 12 | 250 | NA | Femur | COX-2(Etodolac) | 20 | 7 | 3PMB-F,3PMB-Stiff | 21 | Radiog.(scores) | 7,14,21 |
| Endo et al(14) | 2002 | Rats | 12 | 250 | F | Femur | COX-2(Etodolac) | 20 | 21 | 3PMB-F,3PMB-Stiff | 21 | Radiog.(scores) | 7,14,21 |
| Gerstenfeld et al(15) | 2007 | Rats | NA | 449 | M | Femur | NS-COX(Ket.),  COX-2(Valdecoxib) | 4,5 | 7,21 | 3PMB-F,3PMB-Stiff | 21,35 | Hist-mineralization, PGE2 level | 21,35 |
| Gerstenfeld et al(16) | 2003 | Rats | 10 | 430 | M | Femur | NS-COX(Ket.),  COX-2(Valdecoxib) | 4,5 | 21,35 | 3PMB-F,3PMB-Stiff | 21,35 | Hist. | 21,35 |
| Giordano et al(17) | 2003 | Rats |  | 100 | M | Tibia | COX-2(Tenoxicam) | 20 | 7,14,28 | NO | NA | Hist.scores(Allen | 3,7,14,28 |
| Hak et al(18) | 2011 | Rabbit | 48 | 300 | F | Tibia | COX-2(Rof.) | 12.5 | 28 | 3PMB-F,3PMB-Stiff,3PMB-WF | 28 | Hist., COX-1 & COX-2 mRNA level | 28 |
| Herbenick et al (19) | 2008 | Rats | NA | 350 | M | Femur | COX-2(Celec.) | 3.2 | 14,28,56,84 | 3PMB-F | 14,28,56,84 | NO |  |
| Hogevold et al (20) | 1992 | Rats | NA | 338 | M | Femur | NS-COX(Ind.) | 0.2,0.2 | 3 | 3PMB-F,3PMB-Stiff,3PMB-WF | 6 | NO |  |
| Huo et al(21) | 1991 | Rats | Mature | 240 | F | Femur | NS-COX(Ibu.) | 30 | 5days started after 3 days | 3PMB-F,3PMB-Stiff | 14,28,42,56,84 | Hist., BV, (Huo grades) | 14,28,42,84 |
| Inal et al(22) | 2014 | Rats | NA | 250 | M | Fibula | NS-COX(Dicl.,Dexketoprofen)  COX-2(Melo.) | 1,0.98,0.2 | 10 | NO | NA | Hist.Scores (Huo) | 28 |
| Keller et al(23) | 1987 | Rabbit | adult | 430 | NA | Tibia | NS-COX(Ind.) | 10 | 14,42 | 3PMB-F | 14,42 | Hist.Bone tissue | 14,42 |
| Krischak et al(24) | 2007 | Rats | NA | 300 | M | Femur | MS-COX(Dicl.) | 5 | 10 | NO | NA | Hist.Bone density (No.osteoblasts), Radiog. | 10 |
| Krischak et al(25) | 2007 | Rats | NA | 300 | M | Tibia | NS-COX(Dicl.) | 5 | 7,21 | NO | NA | Hist.(tissue type, bone, cartilage, and fibrous) | 21 |
| Li et al(26) | 2013 | Rats | 11 | 276 | F | Femur | COX-2(Celec.) | 21 | 1,4 | 3PMB-F,3PMB-Stiff,3PMB-WF | 42 | Histo-Bone density | 28 |
| Matsumoto et al (27) | 2008 | Rats | 8 | 250 | M | Tibia | NS-COX(Ketoprofen),  COX-2(Celec.) | 1,4 | 3 or till scarifice | NO | NA | Hist.Bone area, Immunohistochemistry | 7,14,21 |
| Mullis et al(28) | 2006 | Mice | 10 | 25 | M | Tibia | NS-COX(Ket.,Ind.),  COX-2(Celec.,Rof.) | 2,2,10/50,1/5) | 28,56,84 | 3PMB-F,3PMB-Stiff | 28,56,84 | Hist.(cartilage area, Trap), Biochemical | 28,56,84 |
| Murnaghan et al (29) | 2006 | Mice | 16 | 45.28 | M | Femur | COX-2(Rof.) | 5 | 24,32 | 3PMB-F,3PMB-Stiff | 24,32 | Histo.Scores(callus size, Fibrous, cartilage,bone),  Radiog.(BD) | 24,32 |
| Reikeraas & Engebretsen(30) | 1998 | Rats | NA | 300 | M | Femur | NS-COX(Ket.,Ind.) | 1,2 | 42 | 3PMB-F,3PMB-Stiff,3PMB-WF | 42 | Radiog. | 42 |
| Sandberg& Aspenberg(31) | 2015 | Mice | 10 | NA | M | Femur | NS-COX(Ind.) | 2 | 7 | 3PMB-F,3PMB-Stiff | 17 | µ-CT.(BV,TV,BV/Tv,T.Th.,T.SP) | 17 |
| Sassioto et al(32) | 2006 | Rats | NA | 355 | M | Femur | NS-COX(Dicl.) | 5 | NA | NO | NA | Hist.(Examination for fibrous, cartilage & bone tissues) | 7,14,21 |
| Sevimli et al(33) | 2013 | Rats | 11.6 | 190 | M | Tibia | NS-COX(Dexketoprofen) | 5 | 14,21,24 | 3PMB-F | 24 | Hist.Scores(Huo),Radiog.Scores | 14,28,56 |
| Simon et al(34) | 2002 | Rats | NA | 584 | M | Femur | NS-COX(Ind.),  COX-2(Celec.,Rof.) | 1,3,4 | 28,42,56 | 3PMB-F,3PMB-Stiff,3PMB-WF | 28,42,56 | Hist.(MT,Callus Size) | 28,42,56 |
| Simon et al(35) | 2007 | Rats | NA | 272 | F | Femur | COX-2(Celec.) | 2,4,8 | Different effect after 15 days and effect of 4mg/kg in different time points | 3PMB-F,3PMB-Stiff | 15,5,10,15,21,28 | Radiog.scores | Different effect after 15 days and effect of 4mg/kg in different time points |
| Singh et al (36) | 2011 | Rabbit | adult | 240 | NA | Femur | COX-2(Etoricoxib) | 3 | 28, 56, 84 | 3PMB-F,3PMB-Stiff | 28,56,84 | Hist. scores,  Radiog. scores,  Morphological | 28,56,84 |
| Spiro et al(37) | 2010 | Mice | 12 | NA | F | Femur | NS-COX(Dicl.) | 5 | 20 | 3PMB-F | 20 | Hist.(MT,OSB & OSC number & Surface),  CT(BV,TV,BV/TV,T.Th,T.Sp) | 20 |
| Tan et al(38) | 2009 | Rabbit | NA | 350 | M | Other | NS-COX(Ibu.),  COX-2(Rof.) | 50,12.5 | 28 | 3PMB-F,3PMB-Stiff | 42, 84 | Hist.(MT, callus Size, Cartilage) | 21,42 |
| Tiseo et al(39) | 2006 | Rats | NA | 341 | NA | Femur | NS-COX(Dicl.),  COX-2(Rof.) | 3,1 | 14,28 | NO | NA | Hist.(callus area, Bone area,bone new formation area), Radiog. | 14,28 |
| Tornkvist et al(40) | 1984 | Rabbit | 16 | 210 | NA | Femur | NS-COX(Ibu.,Ind.) | 15,10 | 56 | 3PMB-F | 56 | NO | NA |
| Utvag et al(41) | 2010 | Rats | 113 | 350 | M | Tibia | NS-COX(Dicl.),  COX-2(Par.) | 1,2 | 7 | 3PMB-F,3PMB-Stiff,3PMB-WF | 30 | DEXA-BD | 30 |
| Bissinger et al 2016(42) | 2016 | Rats | 16 | 500 | M | Femur | NS-COX(Dicl.) | 5,0.5 | 21 | 3PMB-F,3PMB-Stiff | 21 | µ-CT(BV,TV,BV/TV,T.Th,T.S) | 21 |
| Bo et al 1976(43) | 1976 | Rats | Adolescent | 187 | M | Femur | NS-COX(Ind.) | 2 | 6,9,12,18,24 | 3PMB-F,3PMB-Stiff | 6,9,12,18,24 | Hist.(area of mineralization),  Plasma level of Indomethacin | 21 |
| Ochi et al 2011(44) | 2011 | Dogs | 11m | NA | F | Tibia | NS-COX(Carprofen) | 2.2 | 120 | 3PMB-F,3PMB-Stiff | 120 | Hist.(callus area,Cartilage),  Radiog. | 120 |
| Karachalios et al (45) | 2007 | Rabbit | 12 | 340 | M | Ulna | NS-COX(Ind.),  COX-2(Melo.,Rof.) | 2,0.3,0.5,2.5 | 5 | 3PMB-F,3PMB-Stiff. | 42 | Hist.(histomorphometrics- BC,OCC) | 42 |
| Sudman & Bang(46) | 1979 | Rabbit | 18 | 200 | F | Radius | NS-COX(Ind.) | 5,10 | 14,28 | NO | NA | Hist.(Flourochrom label for harvesian system), Indomethcin Plasma level | Not Clear |
| Al-Waeli et al (47) | 2020 | Mice | 4m | 25-28g | M | Tibia | NS-COX(Carprofen) | 20 | 14 | 3PMB | 14 | CBCT,Hist | 14 |
| Gelleher et al (48) | 2019 | Dogs | 1.5y | NA | M | Tibia | NS-COX(Carprofen) | 2.2 | 14d,8w | 3PMB | 14d,8w | Hist.BMD | 14d,8w |

NA-not available; NO-not measured; M-male; F-female; NS-COX-nonselective cyclooxygenase; COX-2-selective cyclooxygenase-2; 3PMB-F-3 points mechanical bending (force to fracture); 3PMB-stiff.- 3 points mechanical bending (stiffness); 3PMB-WF-3 points mechanical bending (work to failure); µ-CT- micro-computertomographic; MT-mineralized tissue; BV-bone volume; BV/TV-bone volume/ tissue volume; T.Th.-trabeculae thickness; T.Sp-trabeculae space; OSB-osteoblast number; OSC-osteclast number; BMD-Bone mineral Density.

|  |  |  |  |  |  |  |  |  |  |  |  |  |  |
| --- | --- | --- | --- | --- | --- | --- | --- | --- | --- | --- | --- | --- | --- |

Table S 5. Subgroup analysis by species regarding the effect of NSAID administration vs control on maximum force to fracture outcome


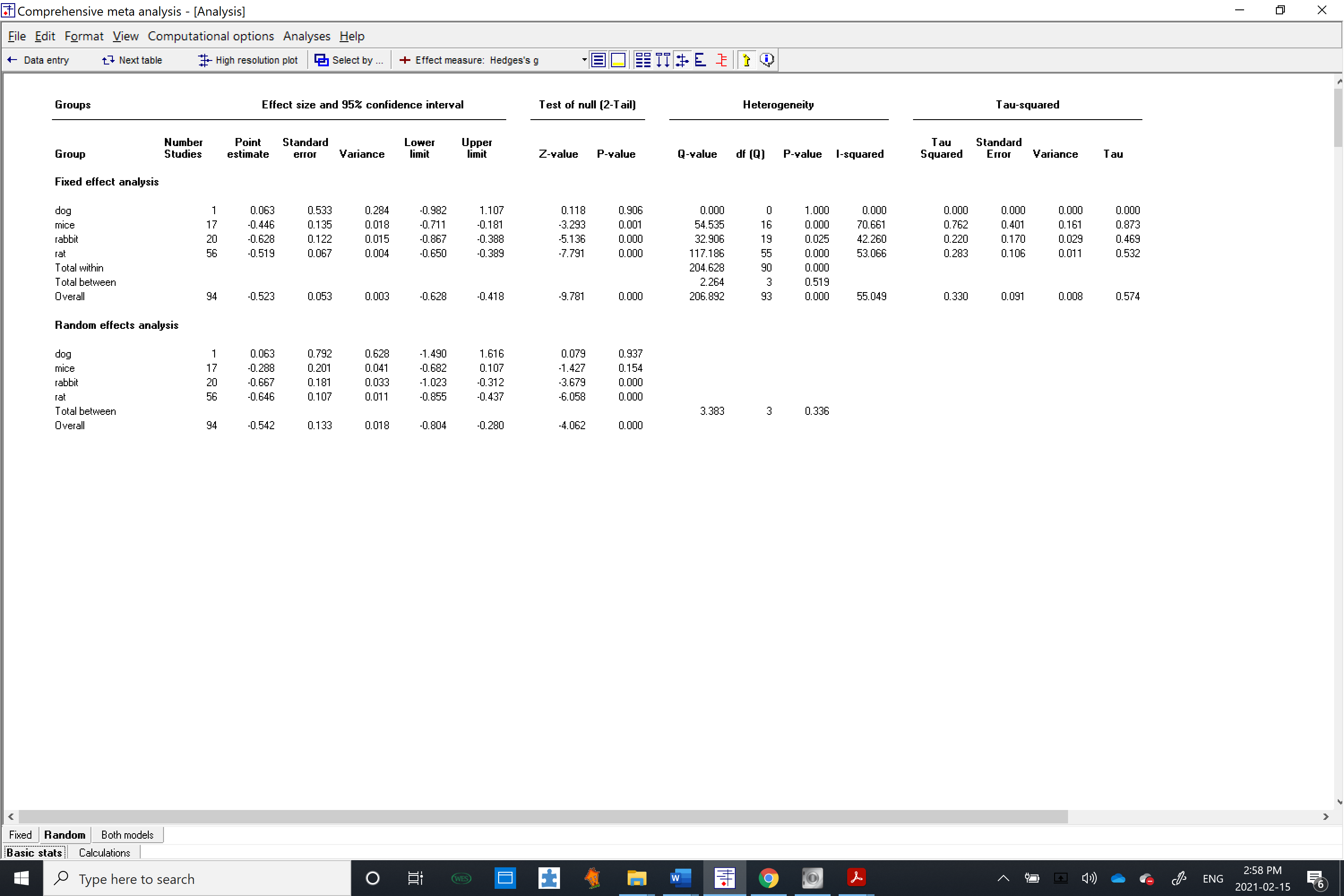


Table S 6. Subgroup analysis by sex regarding the effect of NSAID administration vs control on maximum force to fracture outcome


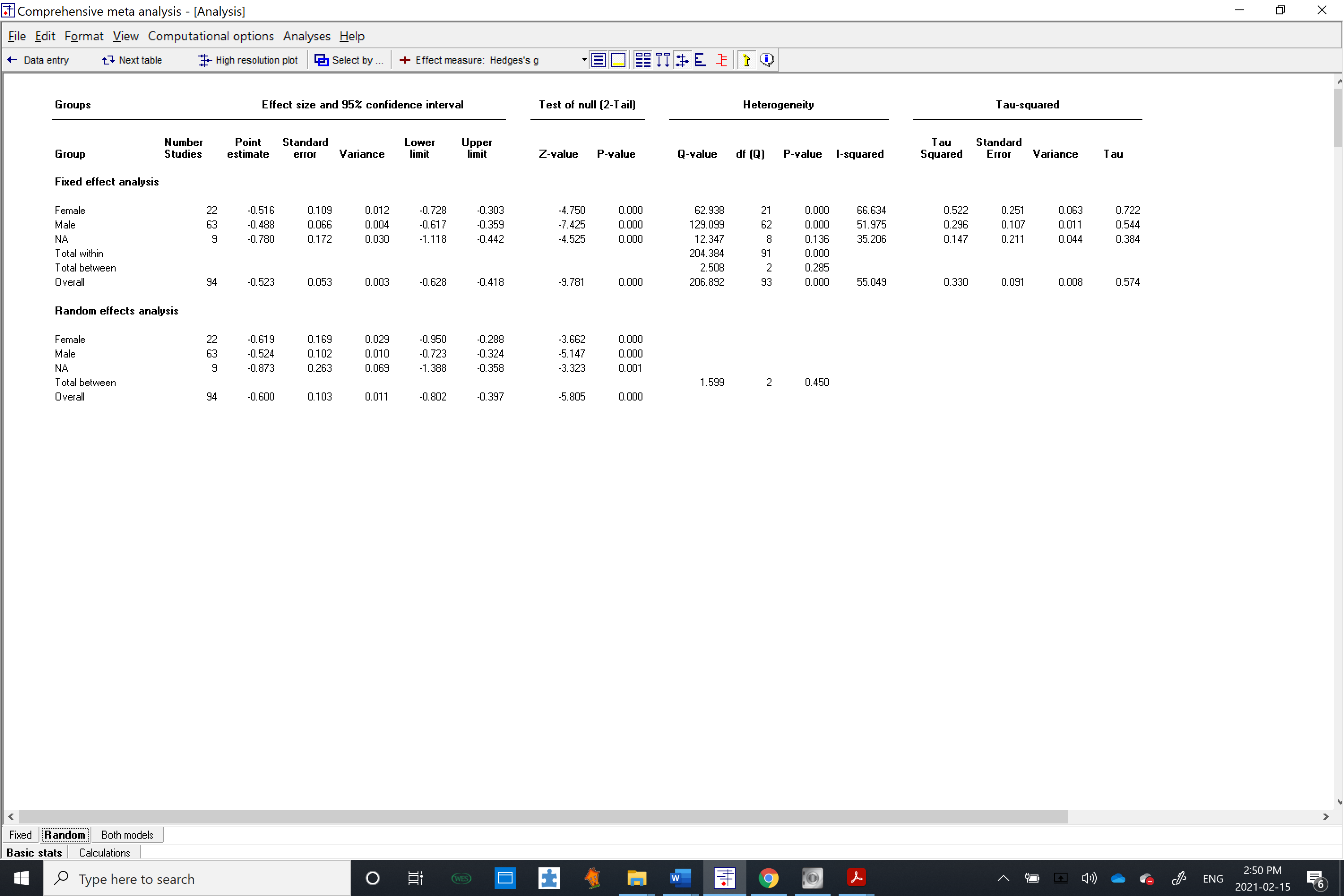


Table S 7. Subgroup analysis by age regarding the effect of NSAID administration vs control on maximum force to fracture outcome (1= <8 wks, 2=8-16wks, >16wks, 4=not mentioned)


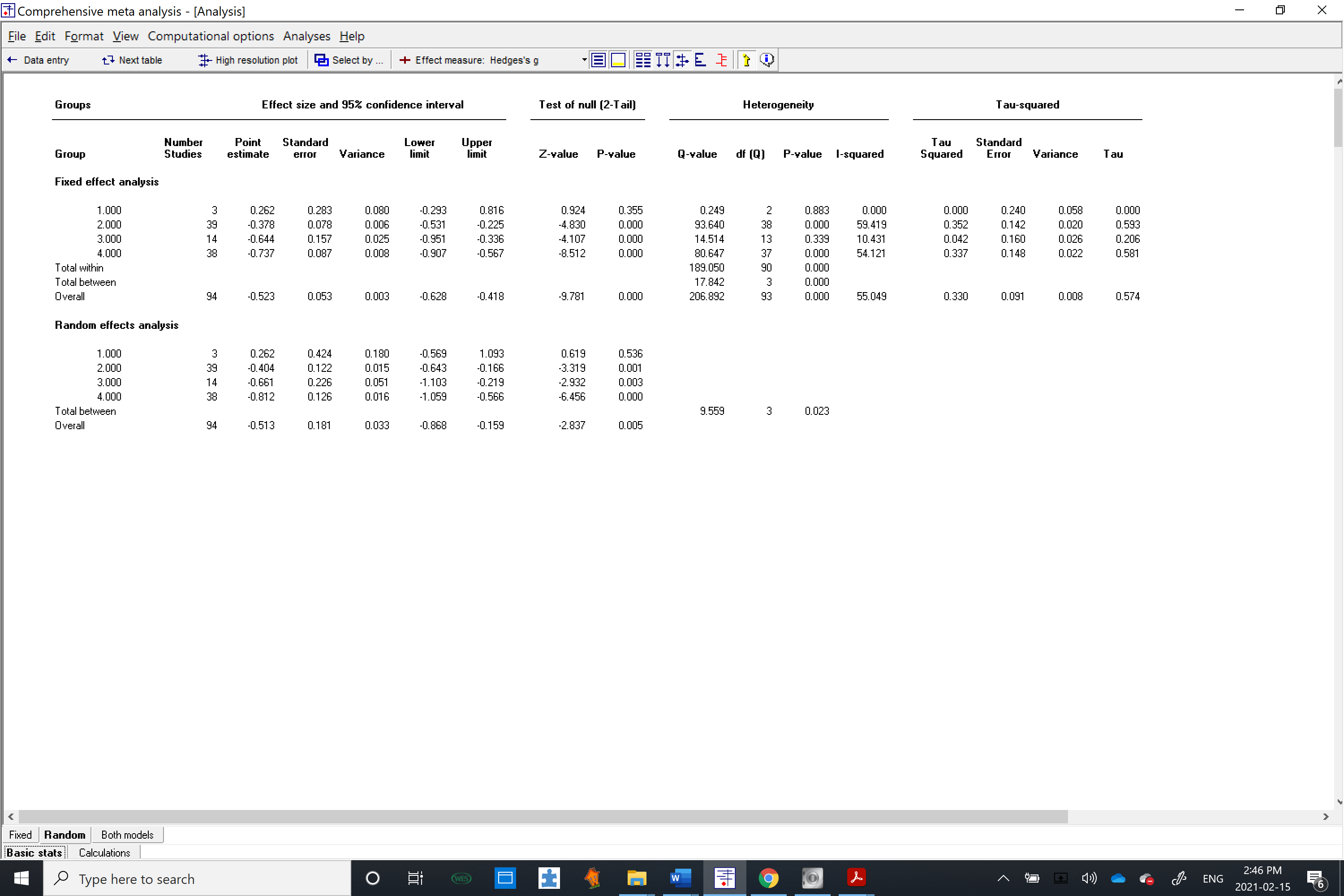


Table S 8. Subgroup analysis by type pf NSAID regarding the effect of NSAID administration vs control on maximum force to fracture outcome


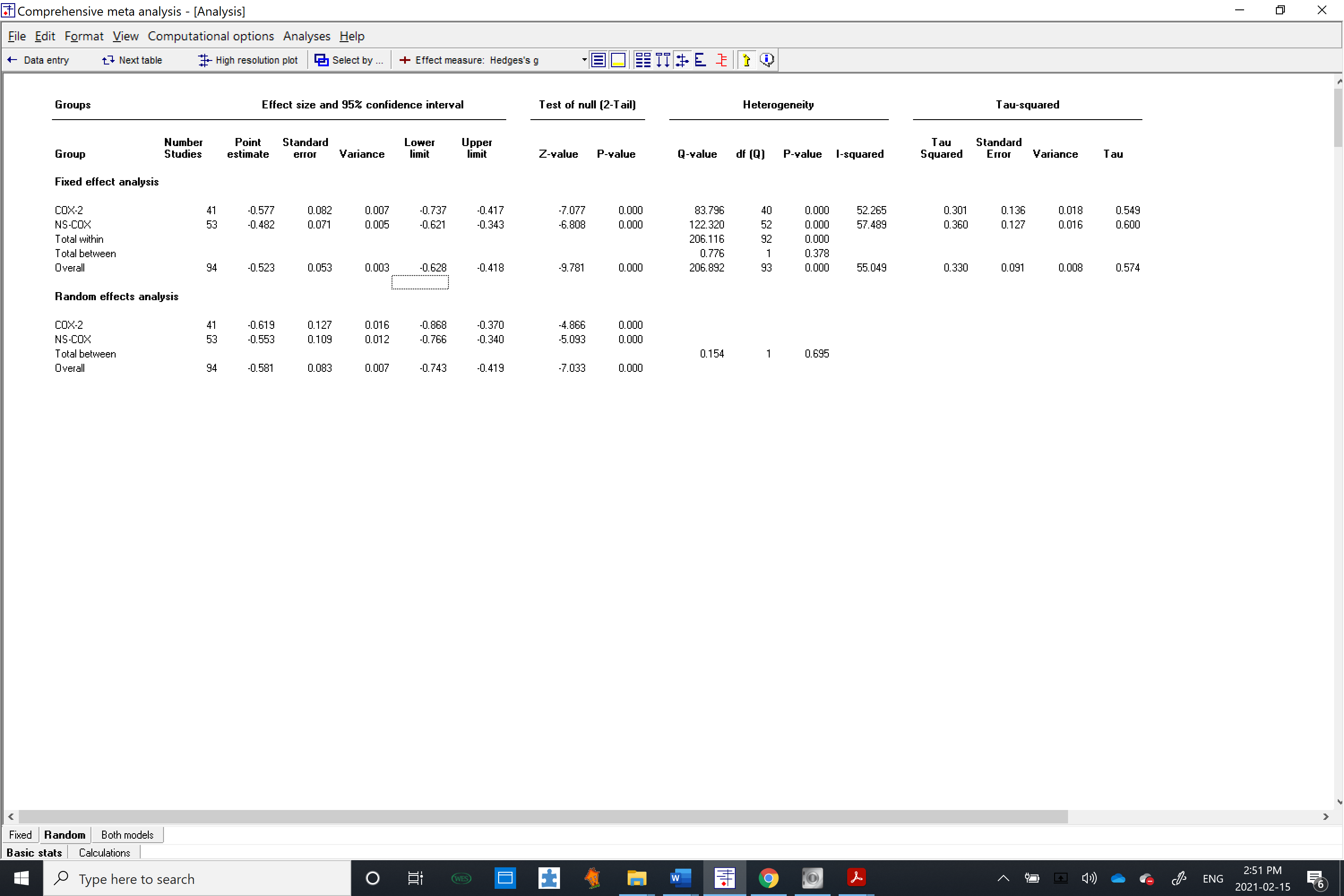


Table S 9. Subgroup analysis by time point regarding the effect of NSAID administration vs control on maximum force to fracture outcome (1=<21days, 2=21-48days, 3=>48days)


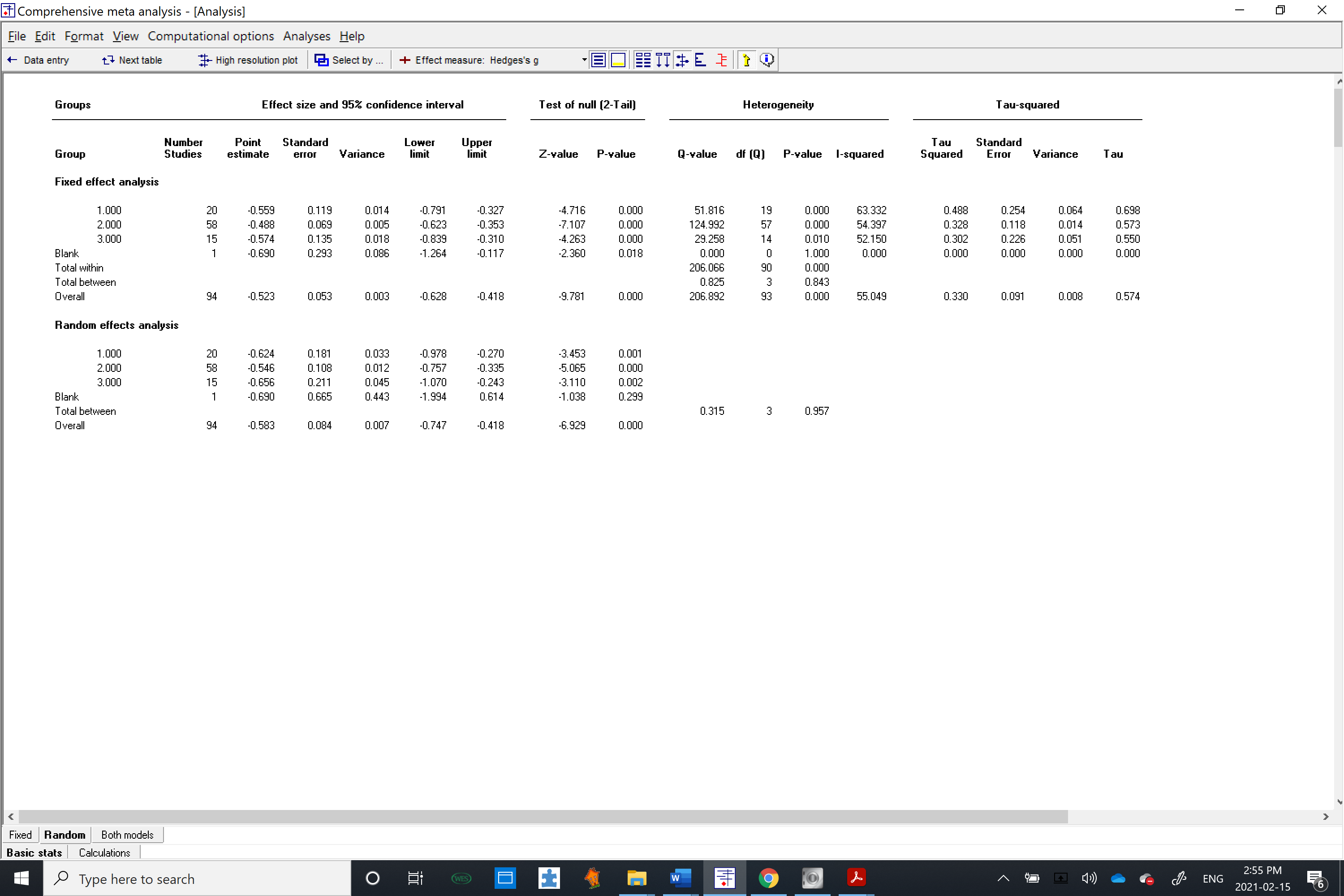


Table S 10. Subgroup analysis by bone fracture site regarding the effect of NSAID administration vs control on maximum force to fracture outcome


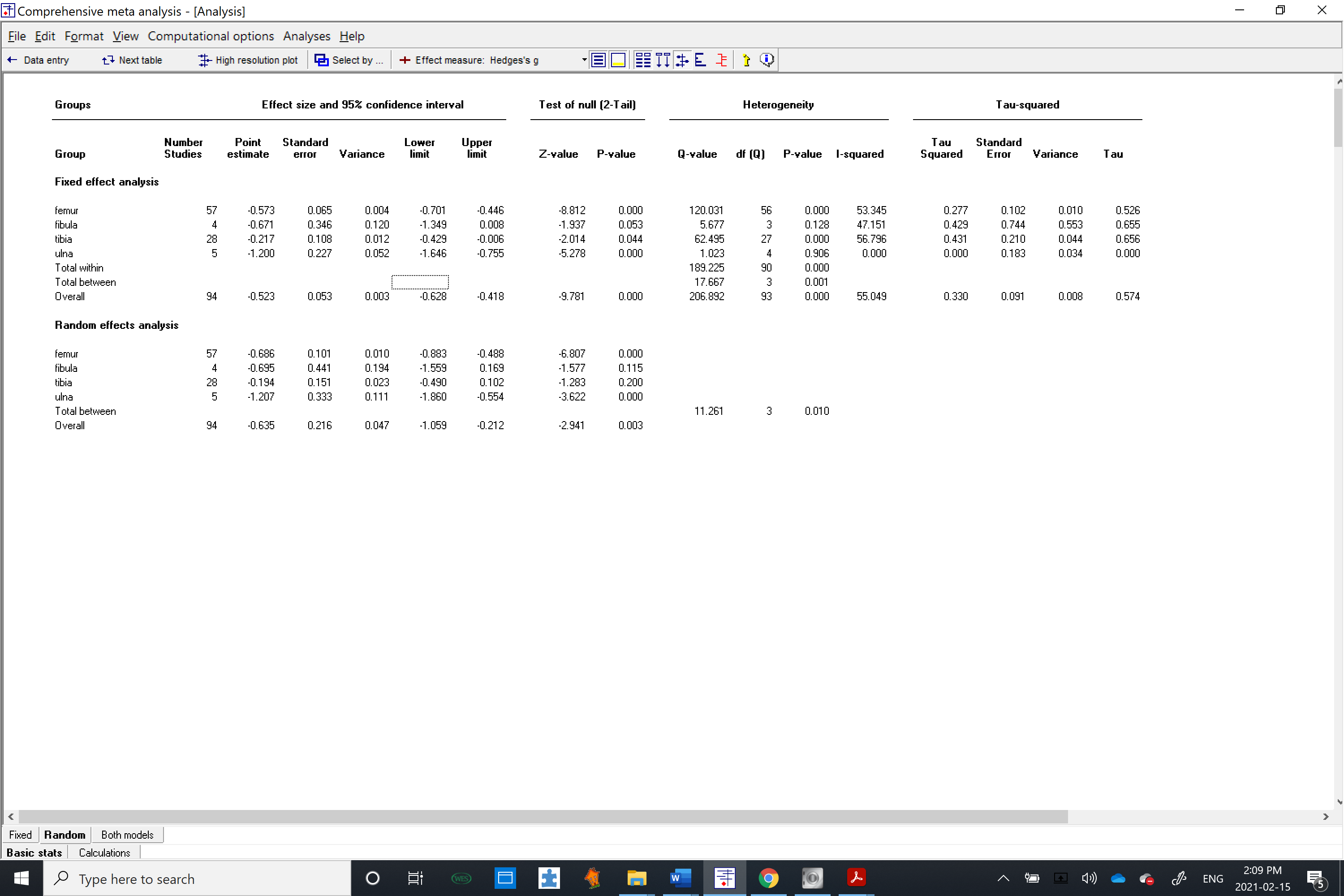


Table S 11. Subgroup analysis by species regarding the effect of NSAID administration vs control on stiffness to fracture outcome


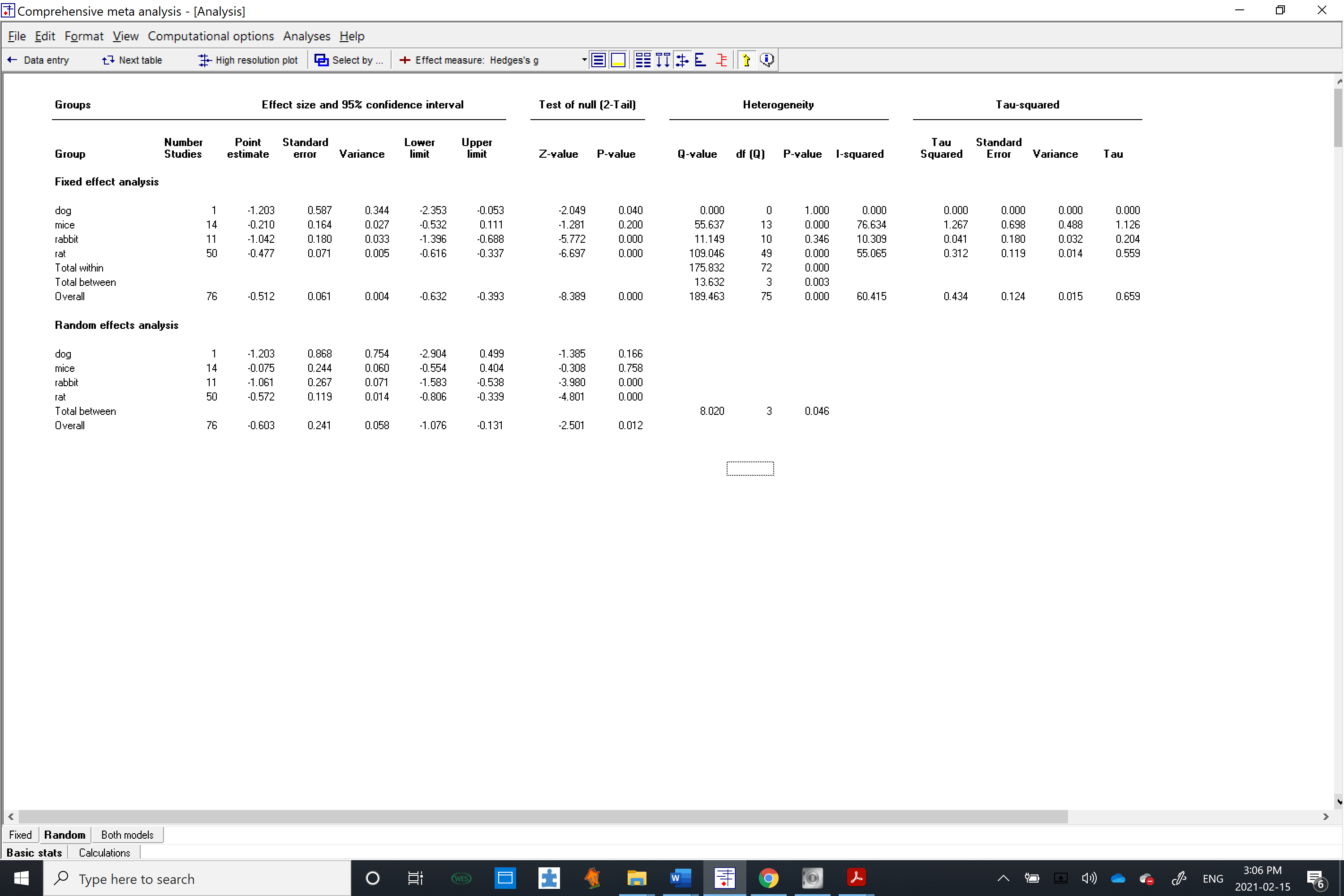


Table S 12. Subgroup analysis by sex regarding the effect of NSAID administration vs control on stiffness to fracture outcome


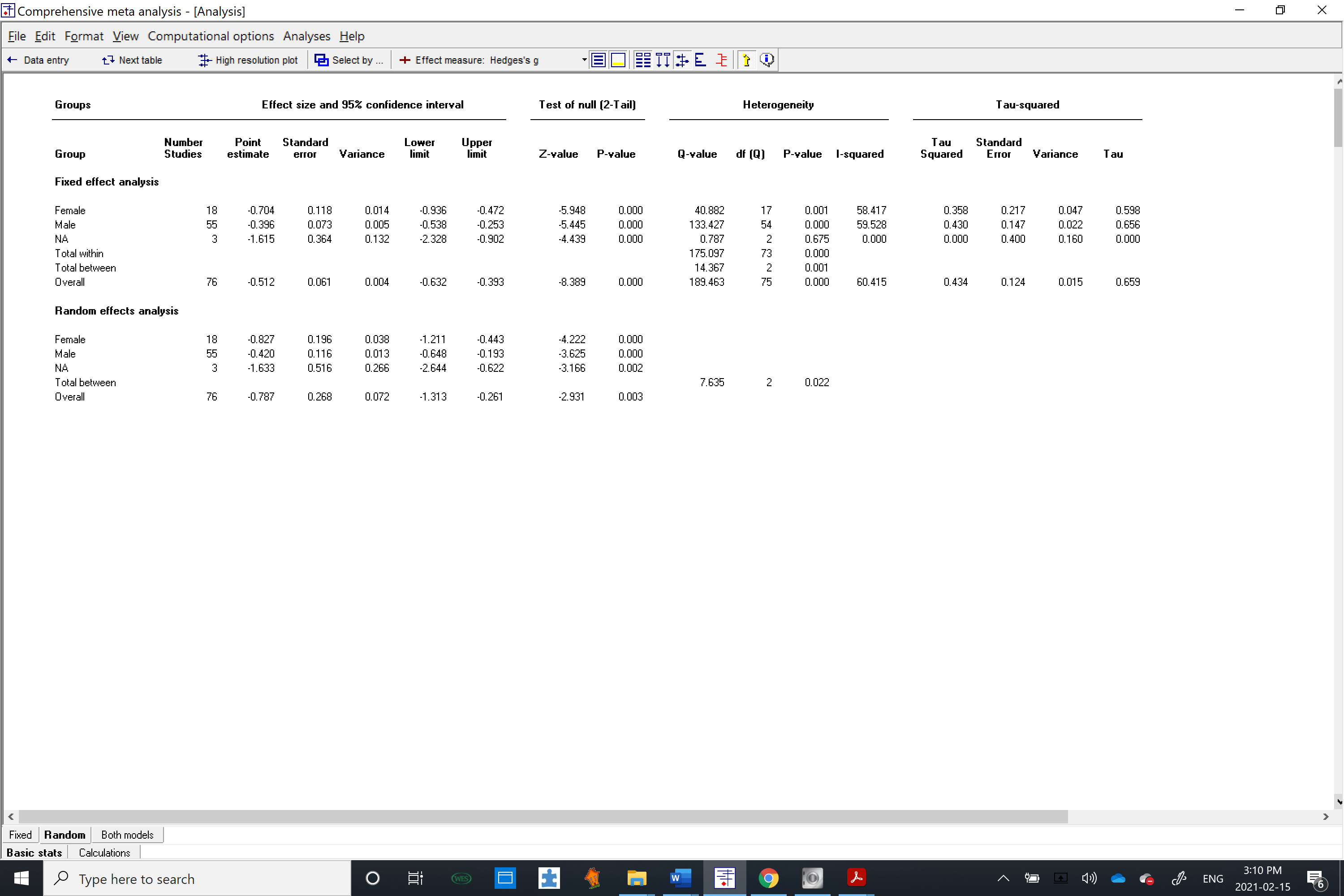


Table S 13. Subgroup analysis by age regarding the effect of NSAID administration vs control on stiffness to fracture outcome (1= <8 wks, 2=8-16wks, >16wks, 4=not mentioned)


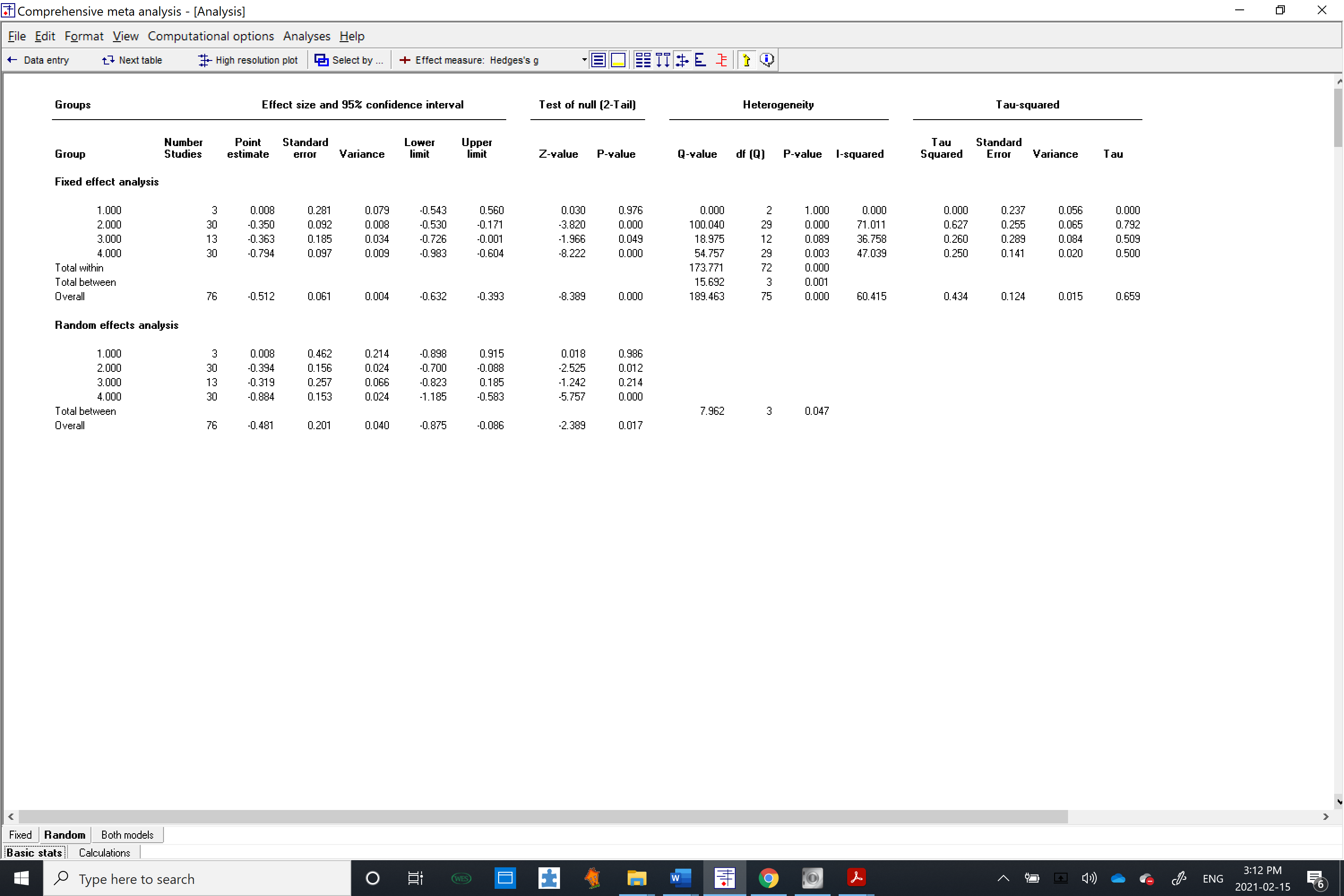


Table S 14. Subgroup analysis by type of NSAID regarding the effect of NSAID administration vs control on stiffness to fracture outcome


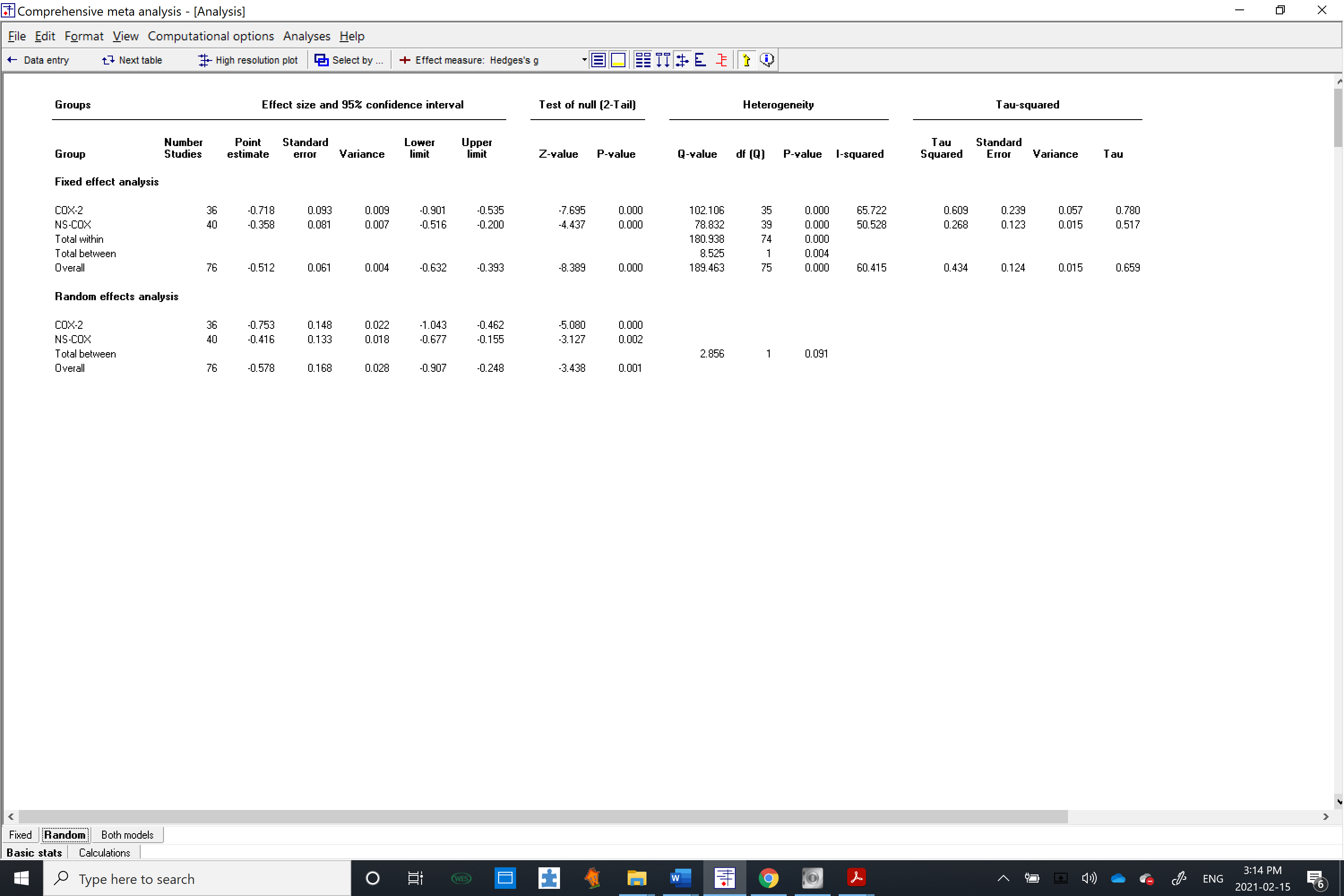


Table S 15. Subgroup analysis by type of time point regarding the effect of NSAID administration vs control on stiffness to fracture outcome (1=<21days, 2=21-48days, 3=>48days)


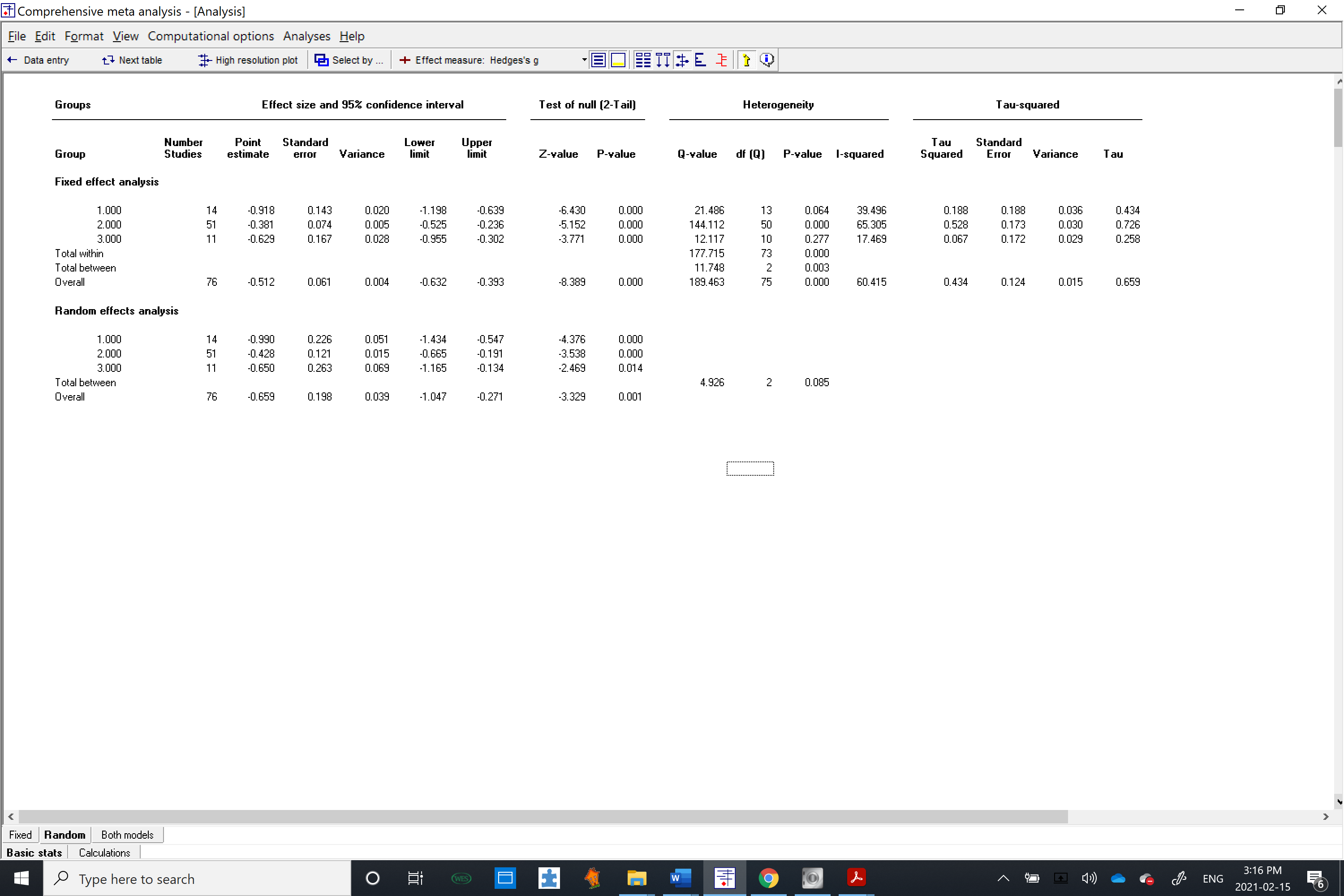


Table S 16. Subgroup analysis by bone fracture site regarding the effect of NSAID administration vs control on stiffness to fracture outcome


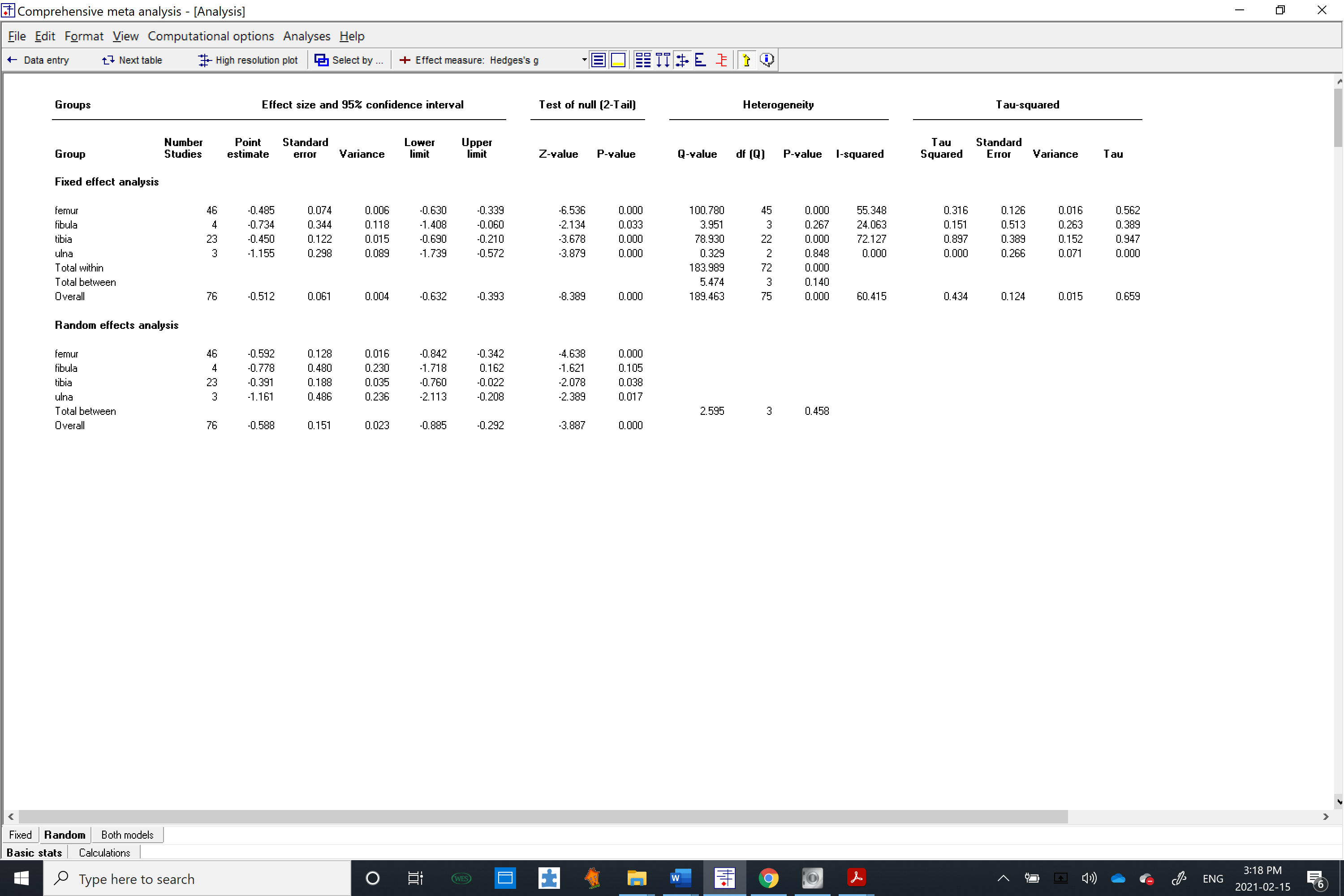


Table S 17. Subgroup analysis by species regarding the effect of NSAID administration vs control on work to failure outcome


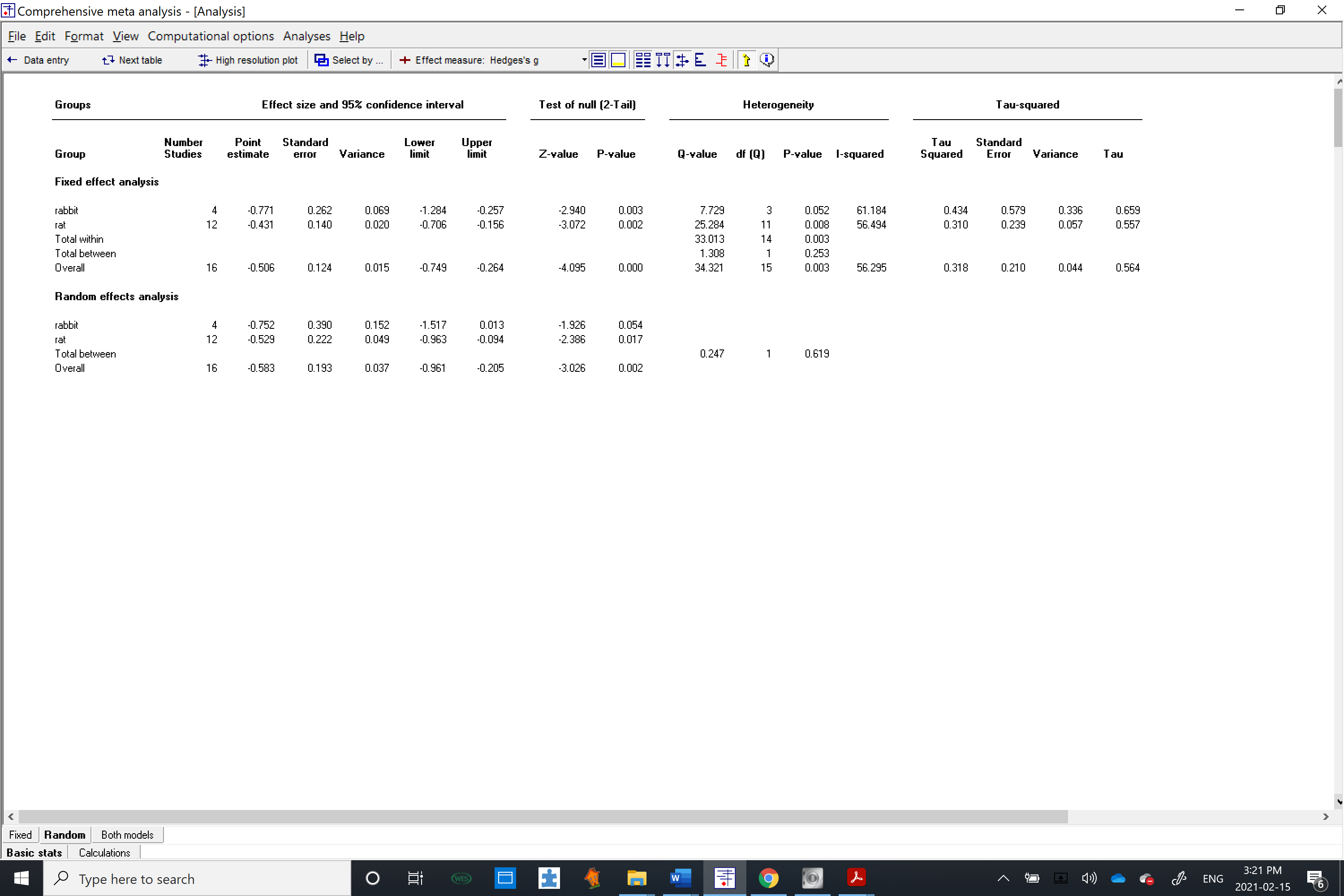


Table S 18. Subgroup analysis by sex regarding the effect of NSAID administration vs control on work to failure outcome


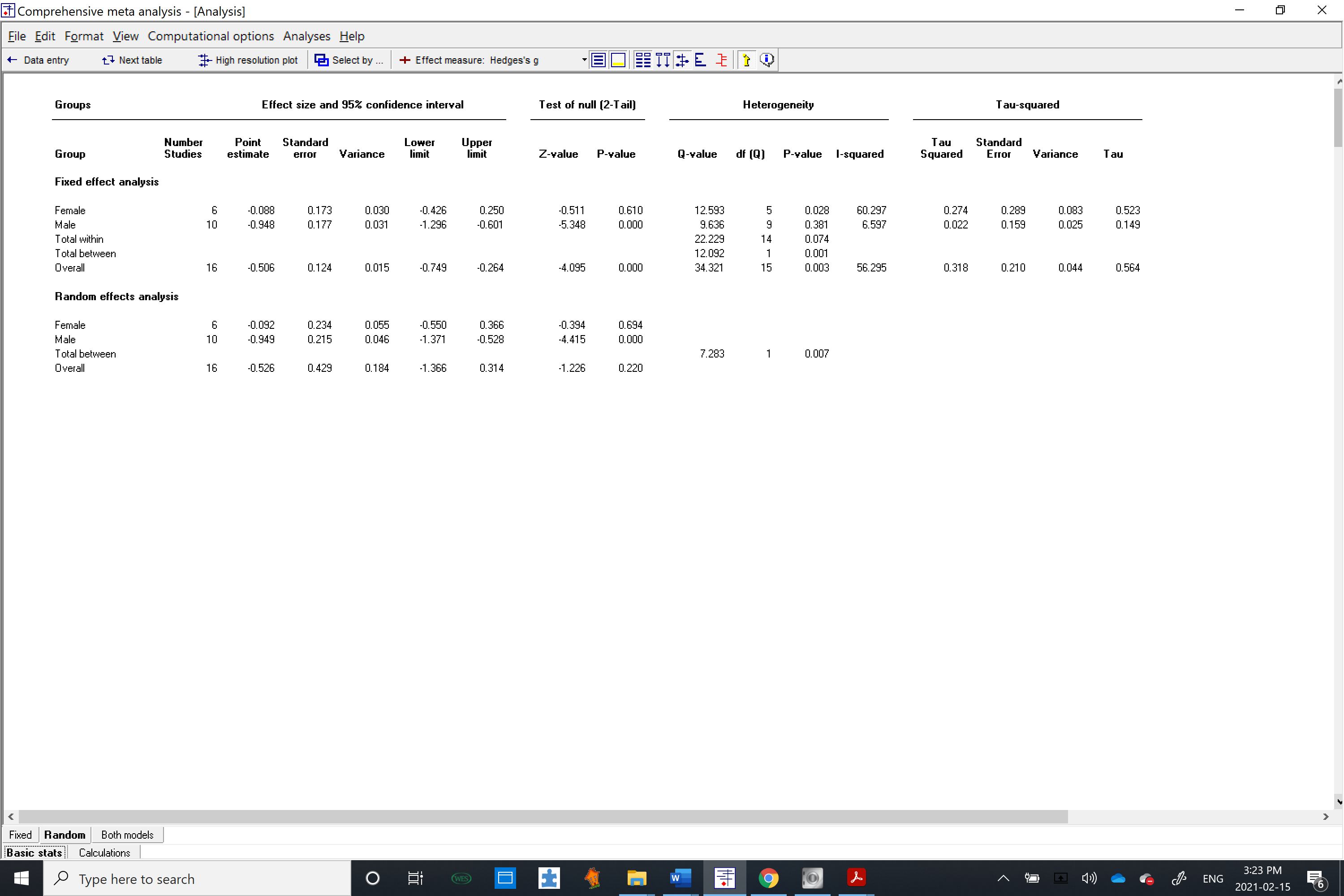


Table S 19. Subgroup analysis by age regarding the effect of NSAID administration vs control on work to failure outcome (1= <8 wks, 2=8-16wks, >16wks, 4=not mentioned)


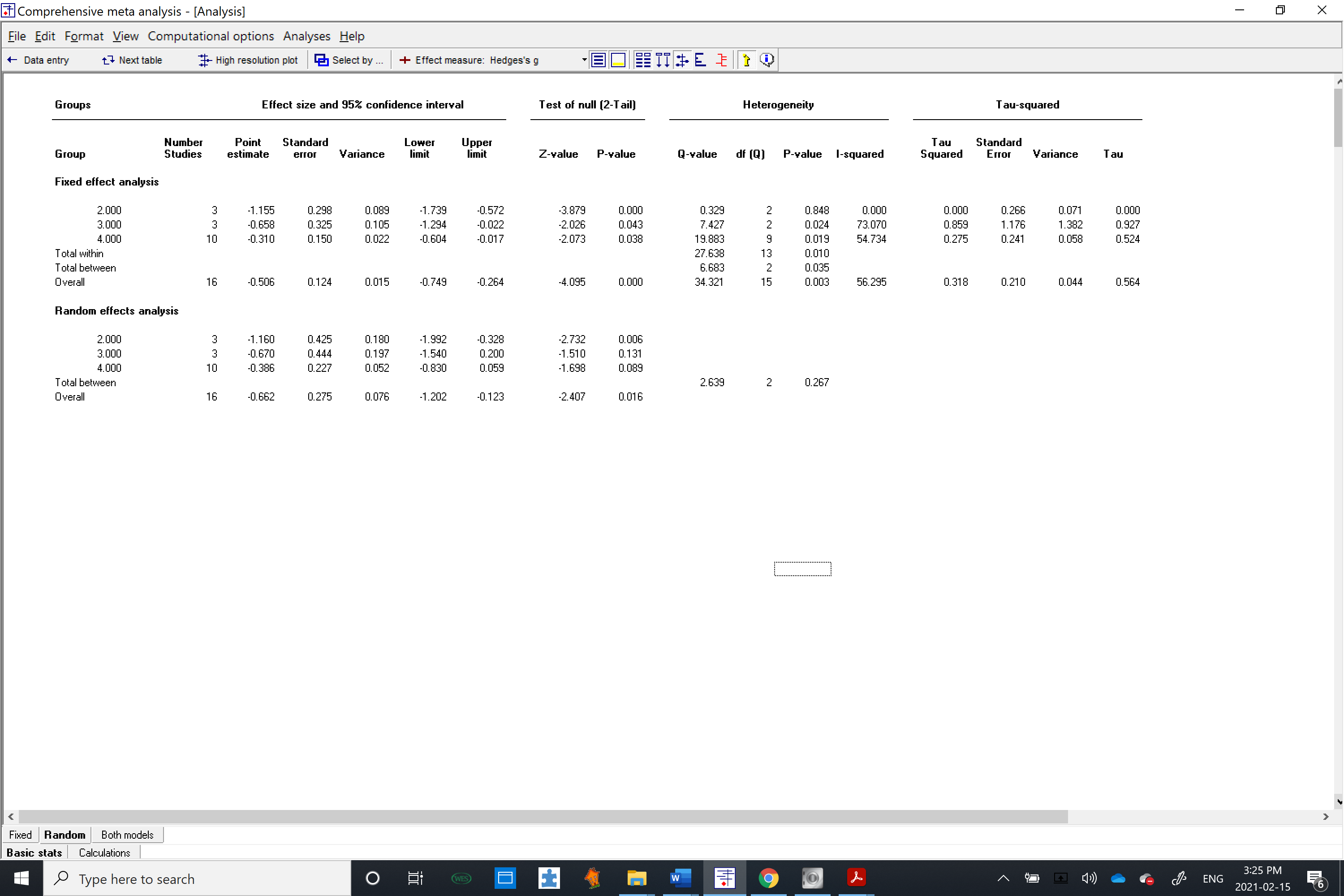


Table S 20. Subgroup analysis by type of NSAID regarding the effect of NSAID administration vs control on work to failure outcome


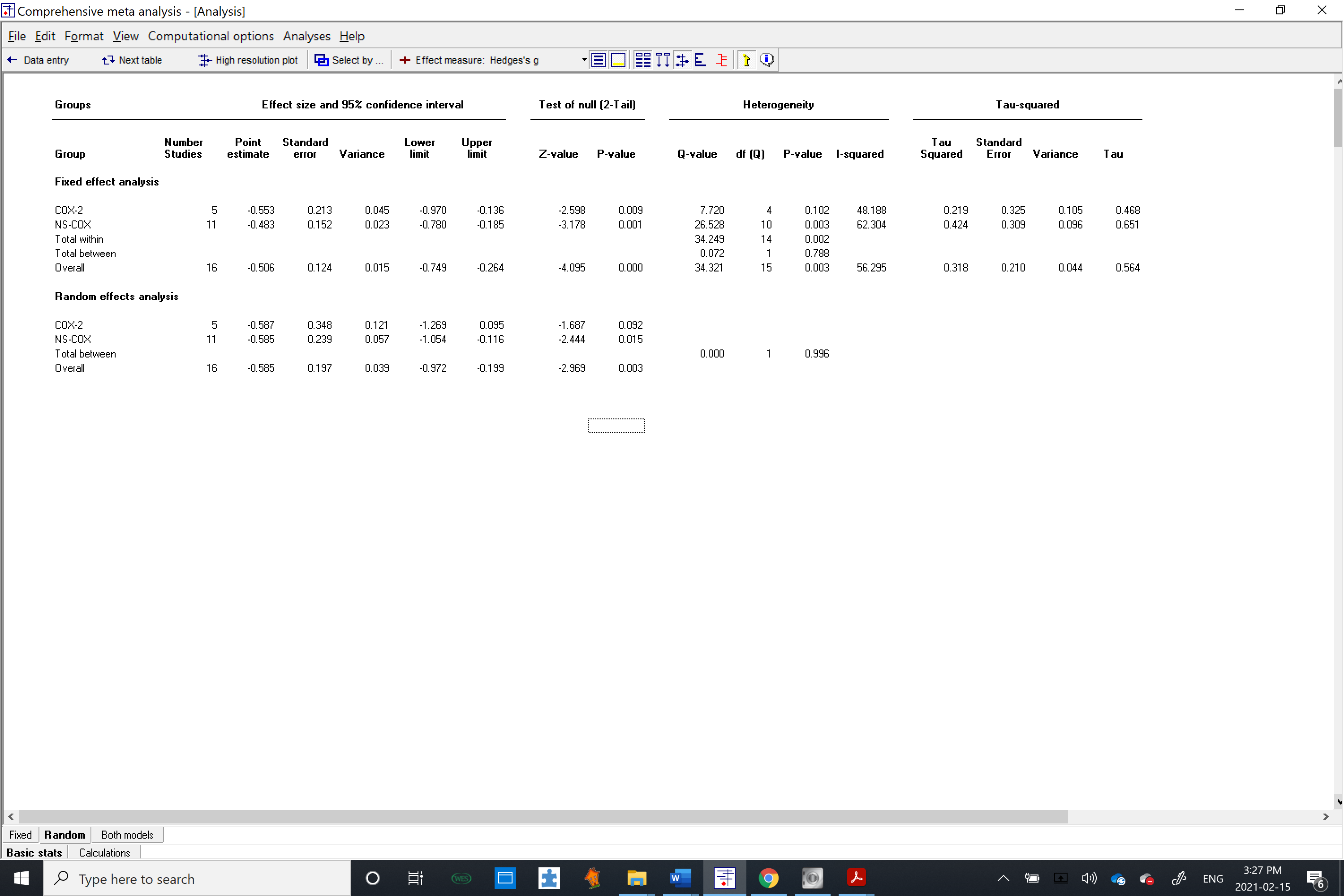


Table S 21. Subgroup analysis by time point regarding the effect of NSAID administration vs control on work to failure outcome (1=<21days, 2=21-48days, 3=>48days)


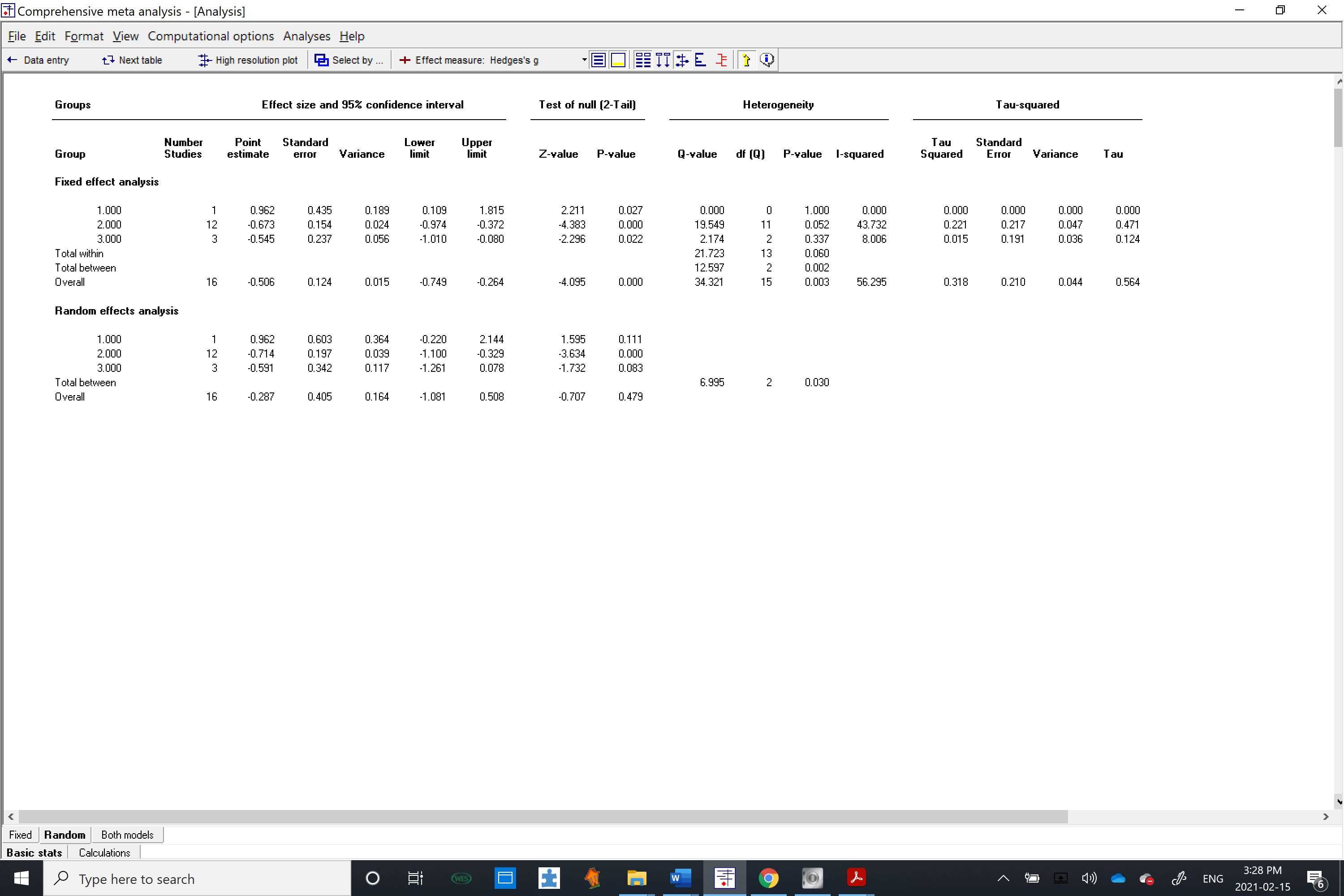


Table S 22. Subgroup analysis by bone fracture site the effect of NSAID administration vs control on work to failure outcome


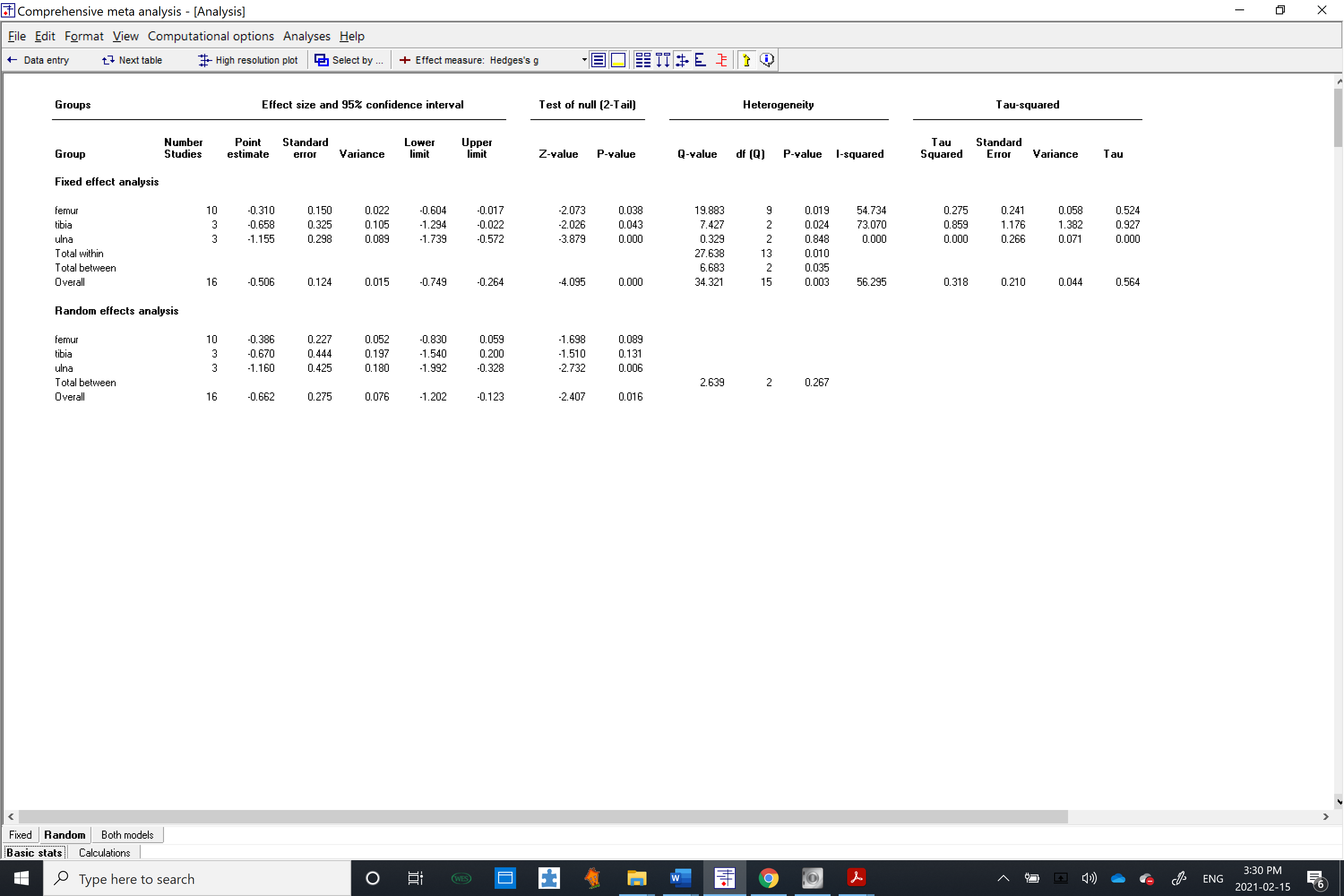


Table S 23. Subgroup analysis by species the effect of NSAID administration vs control on histomorphometric outcome


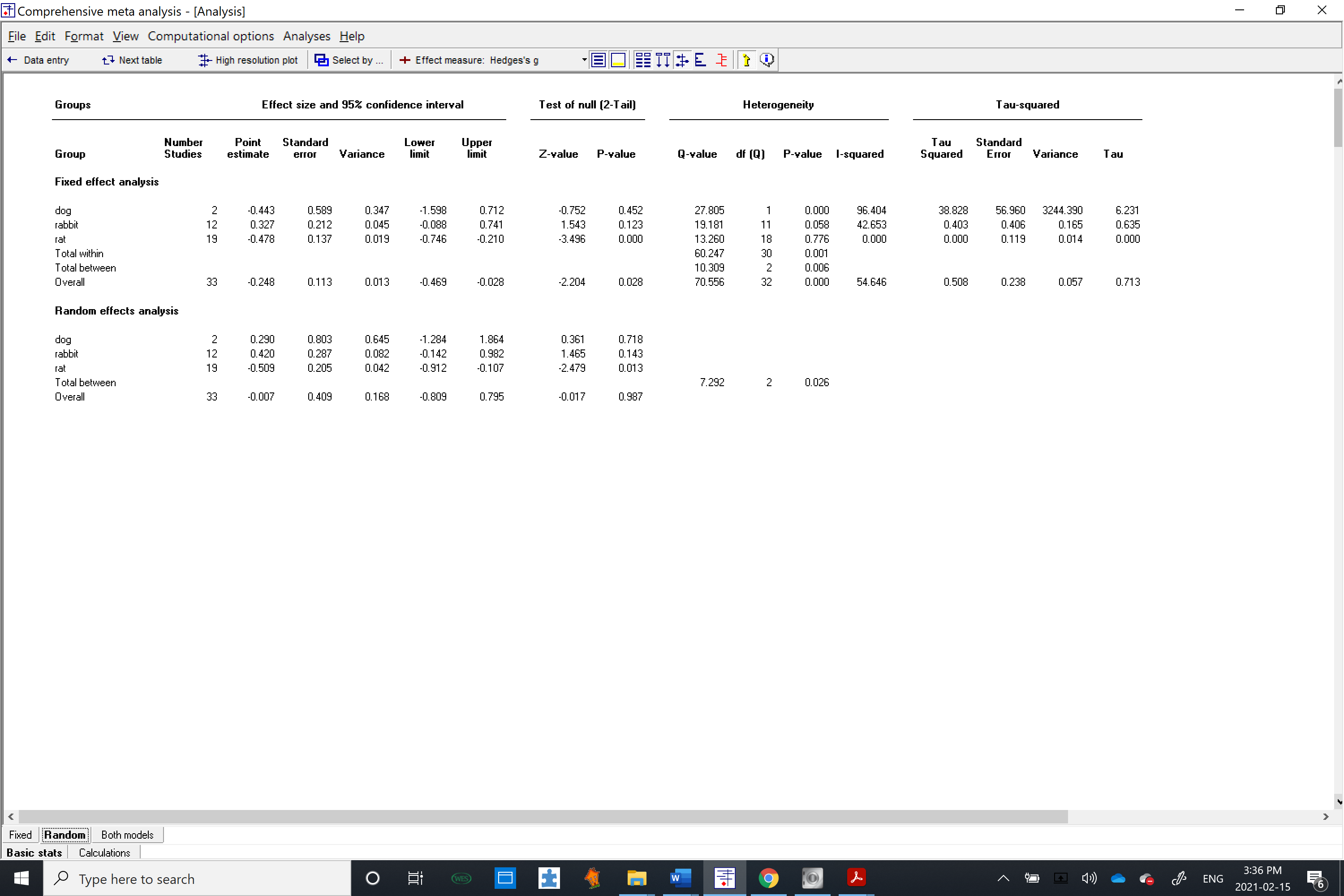


Table S 24. Subgroup analysis by sex the effect of NSAID administration vs control on histomorphometric outcome


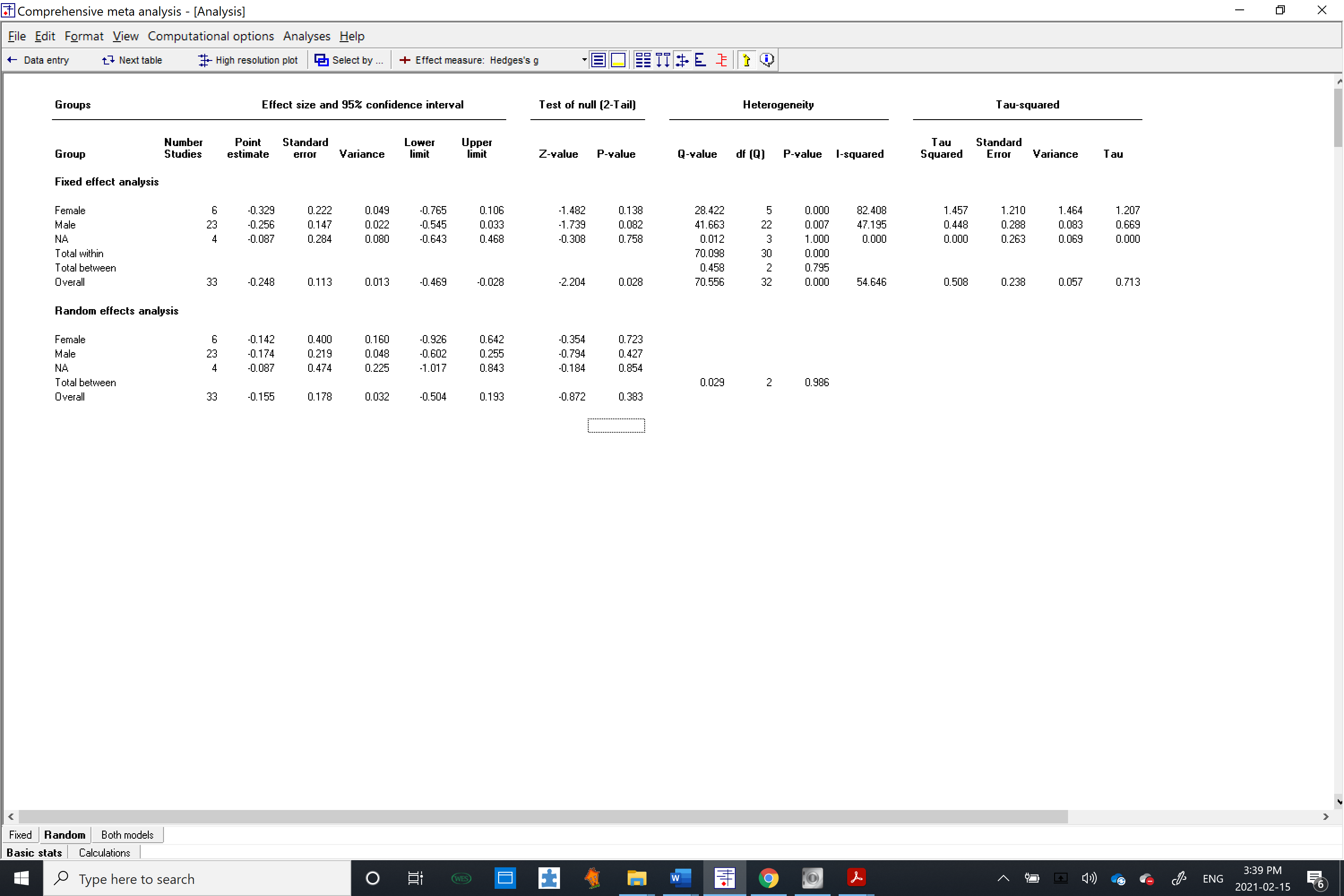


Table S 25. Subgroup analysis by age the effect of NSAID administration vs control on histomorphometric outcome (1= <8 wks, 2=8-16wks, >16wks, 4=not mentioned)


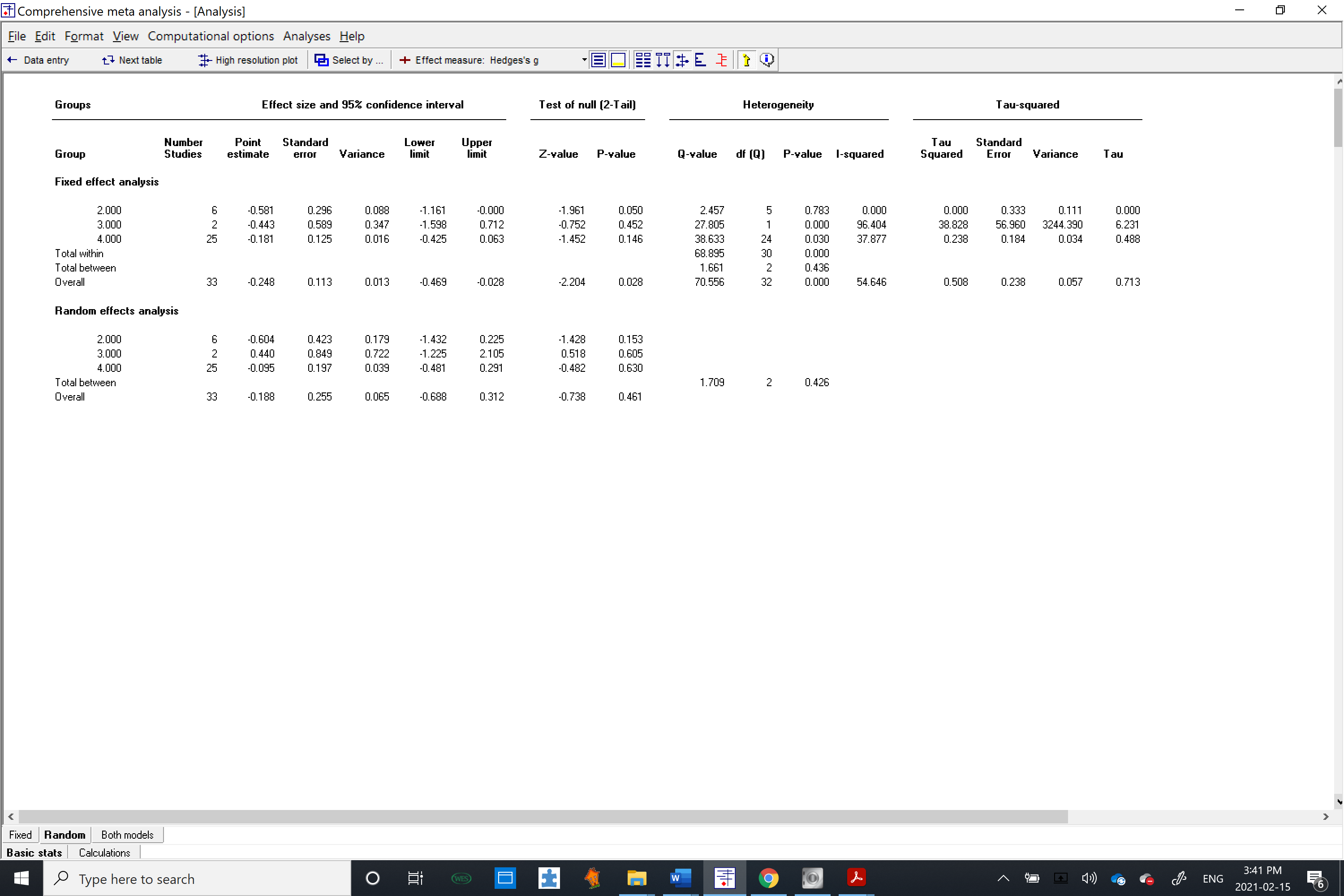


Table S 26. Subgroup analysis by type of NSAID the effect of NSAID administration vs control on histomorphometric outcome


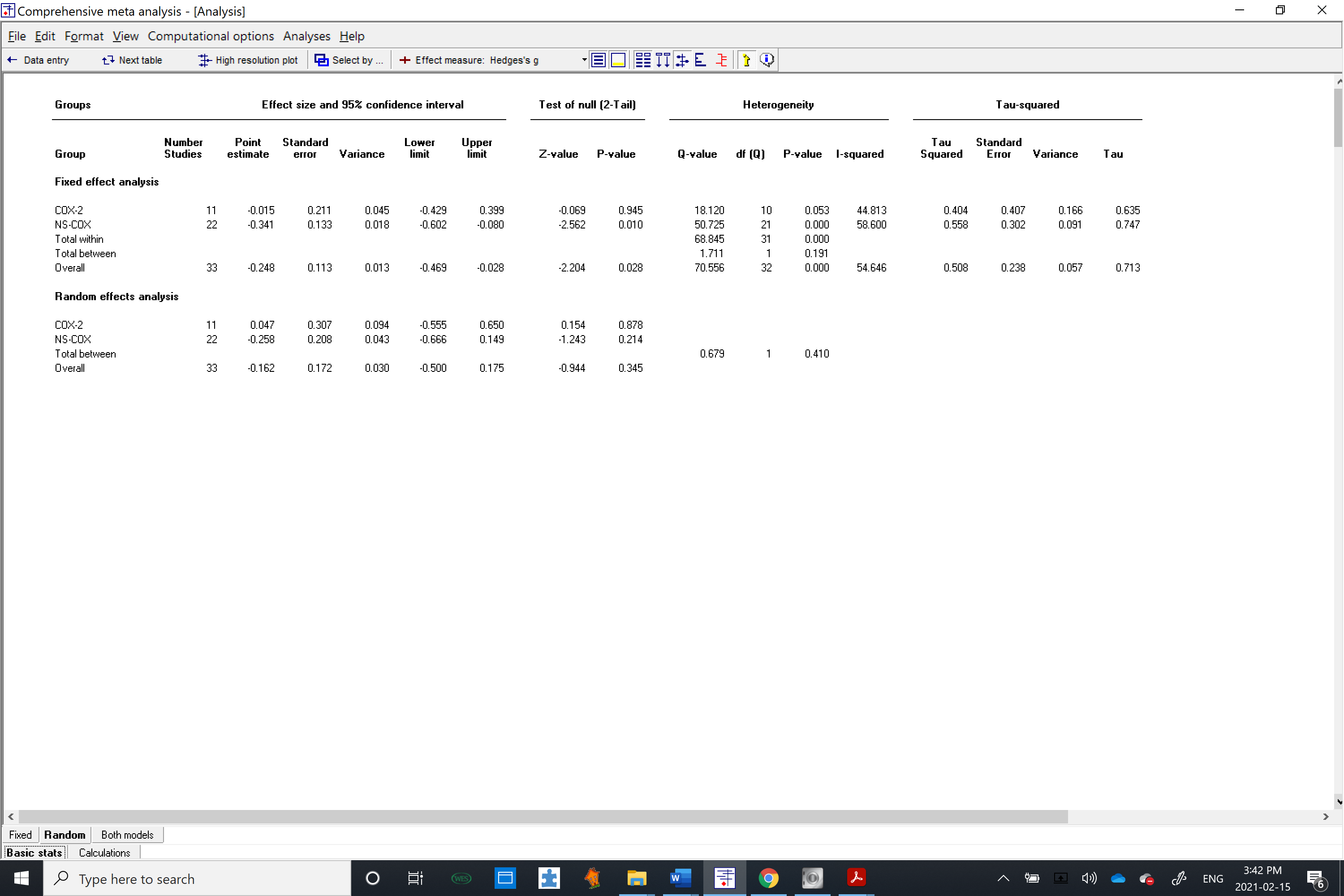


Table S 27. Subgroup analysis by time point the effect of NSAID administration vs control on histomorphometric outcome (1=<21days, 2=21-48days, 3=>48days)


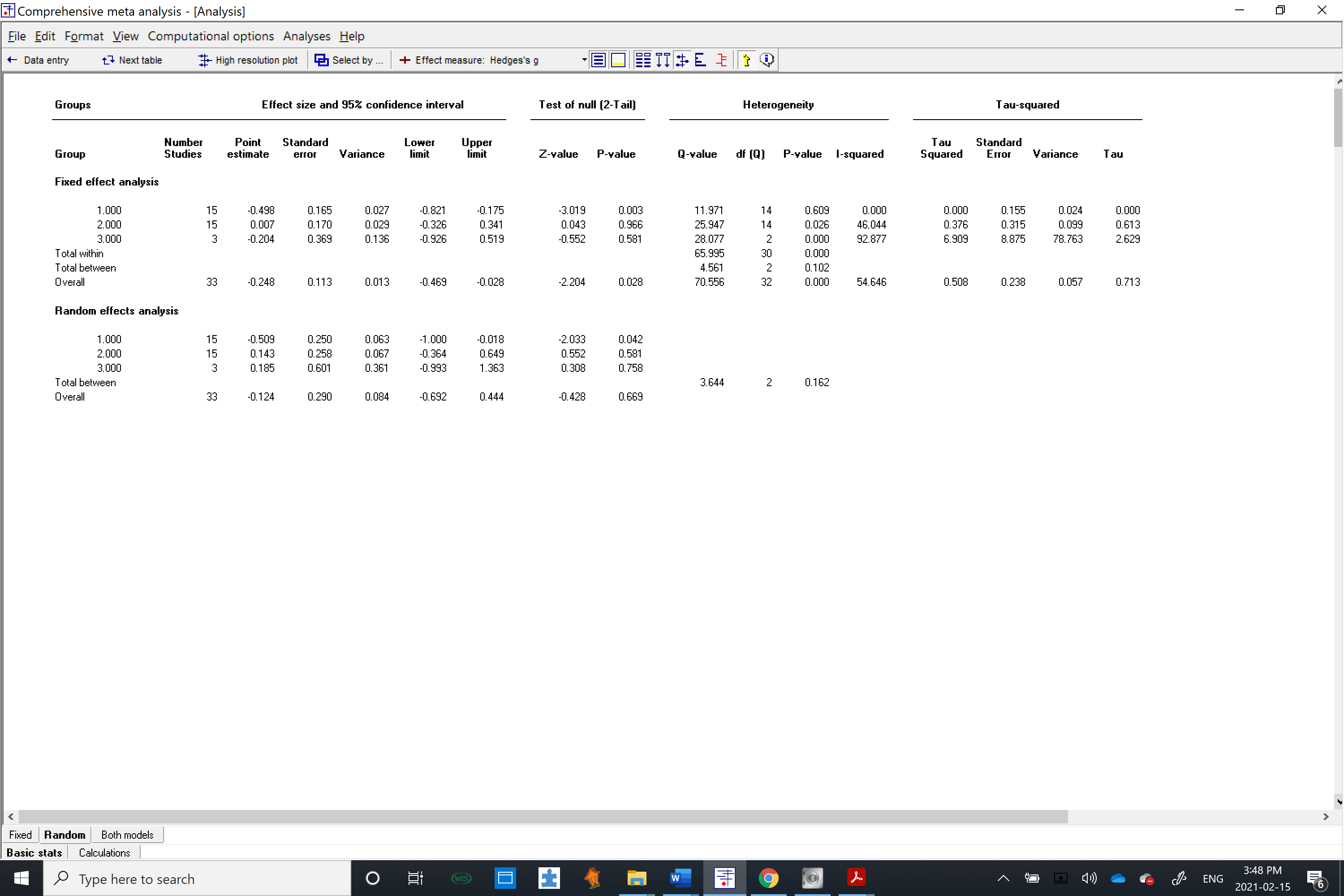


Table S 28. Subgroup analysis by bone fracture site the effect of NSAID administration vs control on histomorphometric outcome


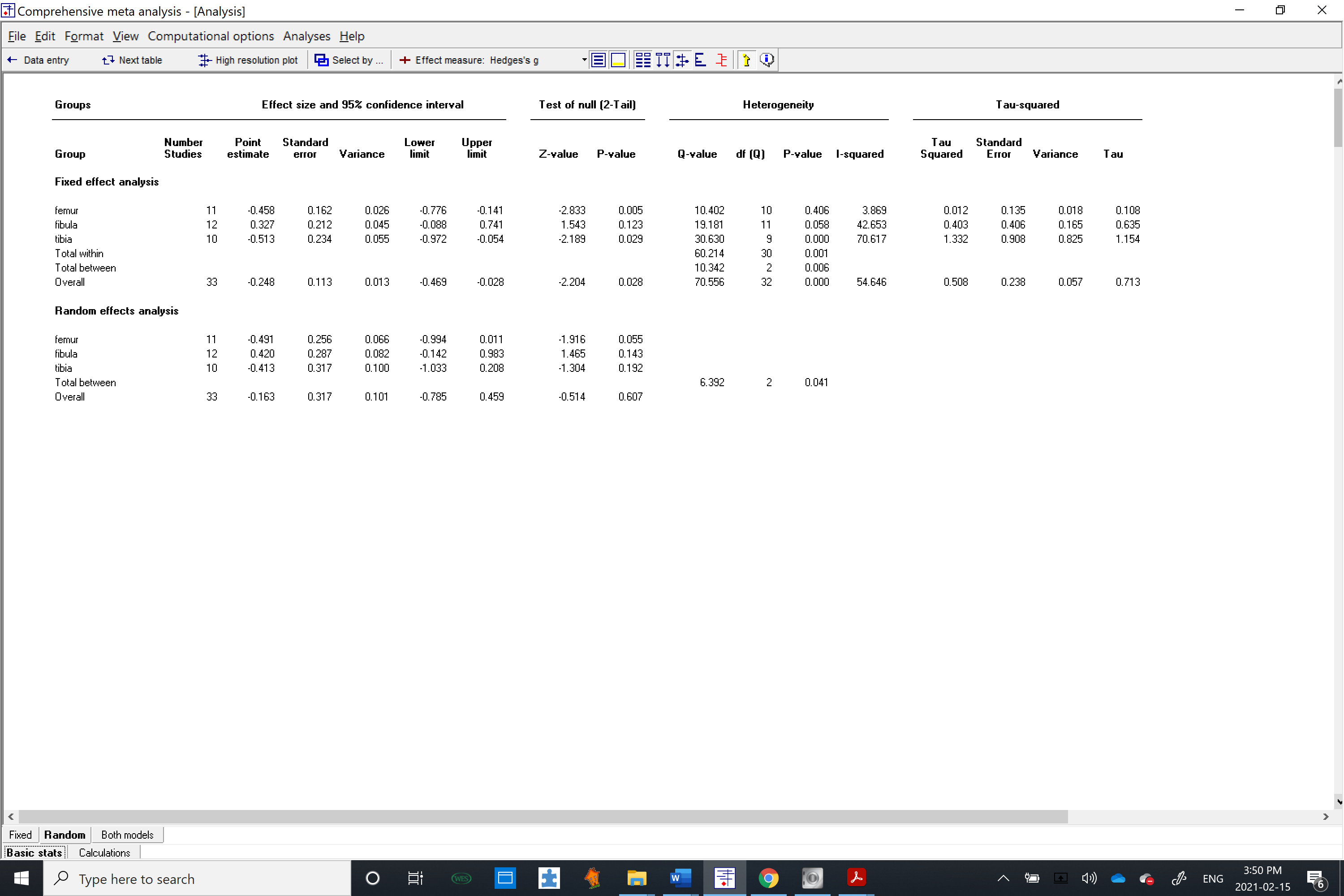


References

1. Hooijmans CR, Rovers MM, de Vries RB, Leenaars M, Ritskes-Hoitinga M, Langendam MW. SYRCLE's risk of bias tool for animal studies. BMC medical research methodology. 2014;14:43.

2. Akman Ş, Gögüs A, Sener N, Bilgiç B, Aksoy B, Seckin F. Effect of diclofenac sodium on union of tibial fractures in rats. Advances in Therapy. 2002;19(3):119-25.

3. Allen HL, Wase A, Bear WT. Indomethacin and aspirin: effect of nonsteroidal anti-inflammatory agents on the rate of fracture repair in the rat. Acta Orthopaedica Scandinavica. 1980;51(4):595-600.

4. Altman RD, Latta LL, Keer R, Renfree K, Hornicek FJ, Banovac K. Effect of nonsteroidal antiinflammatory drugs on fracture healing: a laboratory study in rats. Journal of Orthopaedic Trauma. 1995;9(5):392-400.

5. Beck A, Krischak G, Sorg T, Augat P, Farker K, Merkel U, et al. Influence of diclofenac (group of nonsteroidal anti-inflammatory drugs) on fracture healing. Archives of Orthopaedic & Trauma Surgery. 2003;123(7):327-32.

6. Bergenstock M, Min W, Simon AM, Sabatino C, O'Connor JP. A comparison between the effects of acetaminophen and celecoxib on bone fracture healing rats. Journal of Orthopaedic Trauma. 2005;19(10):717-23.

7. Brown KM, Saunders MM, Kirsch T, Donahue HJ, Reid JS. Effect of COX-2-specific inhibition on fracture-healing in the rat femur. Journal of Bone & Joint Surgery - American Volume. 2004;86(1):116-23.

8. Cappello T, Nuelle JAV, Katsantonis N, Nauer RK, Lauing KL, Jagodzinski JE, et al. Ketorolac administration does not delay early fracture healing in a juvenile rat model: A pilot study. Journal of Pediatric Orthopaedics. 2013;33(4):415-21.

9. Dimmen S, Nordsletten L, Engebretsen L, Steen H, Madsen JE. Negative effect of parecoxib on bone mineral during fracture healing in rats. Acta Orthopaedica. 2008;79(3):438-44.

10. Dimmen S, Nordsletten L, Madsen JE. Parecoxib and indomethacin delay early fracture healing: a study in rats. Clin Orthop. 2009;467(8):1992-9.

11. Donovan M, George M, Petersen E, Fredericks D, Femino JE, Lack WD, et al. Effect of aspirin on bone healing in a rabbit ulnar osteotomy model. Journal of Bone & Joint Surgery - American Volume. 2013;95(6):488-96.

12. Dorey FJ, Kody MH, Kabo JM, More RC, Meals RA. The effects of two nonsteroidal antiinflammatory drugs on limb swelling, joint stiffness, and bone torsional strength following fracture in a rabbit model. Clin Orthop. 1989;0(247):306-12.

13. Endo K, Sairyo K, Komatsubara S, Sasa T, Egawa H, Ogawa T, et al. Cyclooxygenase-2 inhibitor delays fracture healing in rats. Acta Orthopaedica. 2005;76(4):470-4.

14. Endo K, Sairyo K, Komatsubara S, Sasa T, Egawa H, Yonekura D, et al. Cyclooxygenase-2 inhibitor inhibits the fracture healing. Journal of Physiological Anthropology & Applied Human Science. 2002;21(5):235-8.

15. Gerstenfeld LC, Al-Ghawas M, Alkhiary YM, Cullinane DM, Krall EA, Fitch JL, et al. Selective and nonselective cyclooxygenase-2 inhibitors and experimental fracture-healing. Reversibility of effects after short-term treatment. Journal of Bone & Joint Surgery, American Volume. 2007;89(1):114-25.

16. Gerstenfeld LC, Thiede M, Siebert K, Mielke C, Phippard D, Svagr B, et al. Differential inhibition of fracture healing by non-selective and cyclooxygenase-2 selective non-steroidal anti-inflammatory drugs. Journal of Orthopaedic Research. 2003;21(4):670-5.

17. Giordano V, Giordano M, Knackfuss IG, Apfel MIR, Gomes RDC. Effect of tenoxicam on fracture healing in rat tibiae. Injury. 2003;34(2):85-94.

18. Hak DJ, Schulz KS, Khoie B, Hazelwood SJ. The effect of Cox-2 specific inhibition on direct fracture healing in the rabbit tibia. Journal of Orthopaedic Science. 2011;16(1):93-8.

19. Herbenick MA, Sprott D, Stills H, Lawless M. Effects of a cyclooxygenase 2 inhibitor on fracture healing in a rat model. American Journal of Orthopedics (Chatham, Nj). 2008;37(7):E133-7.

20. Hogevold HE, Grogaard B, Reikeras O. Effects of short-term treatment with corticosteroids and indomethacin on bone healing. A mechanical study of osteotomies in rats. Acta Orthopaedica Scandinavica. 1992;63(6):607-11.

21. Huo MH, Troiano NW, Pelker RR, Gundberg CM, Friedlaender GE. The influence of ibuprofen on fracture repair: biomechanical, biochemical, histologic, and histomorphometric parameters in rats. Journal of Orthopaedic Research. 1991;9(3):383-90.

22. Inal S, Kabay S, Cayci MK, Kuru HI, Altikat S, Akkas G, et al. Comparison of the effects of dexketoprofen trometamol, meloxicam and diclofenac sodium on fibular fracture healing, kidney and liver: an experimental rat model. Injury. 2014;45(3):494-500.

23. Keller J, Bak B, Bunger C, Lucht U, Andreassen TT. Bone repair inhibited by indomethacin. Effects on bone metabolism and strength of rabbit osteotomies. Acta Orthopaedica Scandinavica. 1987;58(4):379-83.

24. Krischak GD, Augat P, Blakytny R, Claes L, Kinzl L, Beck A. The non-steroidal anti-inflammatory drug diclofenac reduces appearance of osteoblasts in bone defect healing in rats. Archives of Orthopaedic & Trauma Surgery. 2007;127(6):453-8.

25. Krischak GD, Augat P, Sorg T, Blakytny R, Kinzl L, Claes L, et al. Effects of diclofenac on periosteal callus maturation in osteotomy healing in an animal model. Archives of Orthopaedic & Trauma Surgery. 2007;127(1):3-9.

26. Li K-H, Cheng L, Zhu Y, Deng G-B, Long H-T. Effects of a selective cyclooxygenase-2 inhibitor (celecoxib) on fracture healing in rats. Indian Journal of Orthopaedics. 2013;47(4):395-401.

27. Matsumoto MA, De Oliveira A, Ribeiro Junior PD, Nary Filho H, Ribeiro DA. Short-term administration of non-selective and selective COX-2 NSAIDs do not interfere with bone repair in rats. Journal of Molecular Histology. 2008;39(4):381-7.

28. Mullis BH, Copland ST, Weinhold PS, Miclau T, Lester GE, Bos GD. Effect of COX-2 inhibitors and non-steroidal anti-inflammatory drugs on a mouse fracture model. Injury. 2006;37(9):827-37.

29. Murnaghan M, Li G, Marsh DR, Murnaghan M, Li G, Marsh DR. Nonsteroidal anti-inflammatory drug-induced fracture nonunion: an inhibition of angiogenesis? Journal of Bone & Joint Surgery, American Volume. 2006;88:140-7.

30. Reikeraas O, Engebretsen L. Effects of ketoralac tromethamine and indomethacin on primary and secondary bone healing. An experimental study in rats. Archives of Orthopaedic & Trauma Surgery. 1998;118(1):50-2.

31. Sandberg O, Aspenberg P. Different effects of indomethacin on healing of shaft and metaphyseal fractures. Acta Orthopaedica. 2015;86(2):243-7.

32. Sassioto MC, Inouye CM, Aydos RD, Figueiredo AS. Bone repair in rats treated with sodic diclofenac and calcitonin. Acta Cirurgica Brasileira. 2006;21:40-4.

33. Sevimli R, Üzel M, Sayar H, Kalender AM, Dökmeci O. The effect of dexketoprofen trometamol on the healing of diaphysis fractures of rat tibia. Acta Orthopaedica et Traumatologica Turcica. 2013;47(6):423-9.

34. Simon AM, Manigrasso MB, O'Connor JP. Cyclo-oxygenase 2 function is essential for bone fracture healing. Journal of Bone & Mineral Research. 2002;17(6):963-76.

35. Simon AM, O'Connor JP, Simon AM, O'Connor JP. Dose and time-dependent effects of cyclooxygenase-2 inhibition on fracture-healing. Journal of Bone & Joint Surgery, American Volume. 2007;89(3):500-11.

36. Singh A, Shekhar S, Saraf SK, Garbyal RS. Effect of etoricoxib on fracture healing - an experimental study. Biomedical Research. 2011;22(1):52-6.

37. Spiro AS, Beil FT, Baranowsky A, Barvencik F, Schilling AF, Nguyen K, et al. BMP-7-induced ectopic bone formation and fracture healing is impaired by systemic NSAID application in C57BL/6-mice. Journal of Orthopaedic Research. 2010;28(6):785-91.

38. Tan V, Parsons JR, O'Connor JP, Manigrasso MB, Capo JT, Cottrell JA, et al. A comparison of the effects of ibuprofen and rofecoxib on rabbit fibula osteotomy healing. Acta Orthopaedica. 2009;80(5):597-605.

39. Tiseo BC, Namur GN, De Paula EJL, Mattar Jr R, De Oliveira CRGCM. Experimental study of the action of COX-2 selective nonsteroidal anti-inflammatory drugs and traditional anti-inflammatory drugs in bone regeneration. Clinics. 2006;61(3):223-30.

40. Tornkvist H, Stromberg L, Lindholm TS, Netz P, Lindholm TC. Effect of ibuprofen and indomethacin on bone metabolism reflected in bone strength. Clin Orthop. 1984;0(187):255-9.

41. Utvag SE, Fuskevag OM, Shegarfi H, Reikeras O. Short-term treatment with COX-2 inhibitors does not impair fracture healing. Journal of Investigative Surgery. 2010;23(5):257-61.

42. Bissinger O, Kreutzer K, Gotz C, Hapfelmeier A, Pautke C, Vogt S, et al. A biomechanical, micro-computertomographic and histological analysis of the influence of diclofenac and prednisolone on fracture healing in vivo. BMC musculoskeletal disorders. 2016;17(1):383.

43. Bo J, Sudmann E, Marton PF. Effect of indomethacin on fracture healing in rats. Acta Orthop Scand. 1976;47(6):588-99.

44. Ochi H, Hara Y, Asou Y, Harada Y, Nezu Y, Yogo T, et al. Effects of long-term administration of carprofen on healing of a tibial osteotomy in dogs. American Journal of Veterinary Research. 2011;72(5):634-41.

45. Karachalios T, Boursinos L, Poultsides L, Khaldi L, Malizos KN. The effects of the short-term administration of low therapeutic doses of anti-COX-2 agents on the healing of fractures. An experimental study in rabbits. The Journal of bone and joint surgery British volume. 2007;89(9):1253-60.

46. Sudmann E, Bang G. Indomethacin-induced inhibition of haversian remodelling in rabbits. Acta Orthop Scand. 1979;50(6 Pt 1):621-7.

47. Al-Waeli H, Nicolau B, Stone L, Abu Nada L, Gao Q, Abdallah MN, et al. Chronotherapy of Non-Steroidal Anti-Inflammatory Drugs May Enhance Postoperative Recovery. Scientific Reports.10(1):468.

48. Gallaher HM, Butler JR, Wills RW, Priddy LB, Elder SH, Heller SM, et al. Effects of short- and long-term administration of nonsteroidal anti-inflammatory drugs on osteotomy healing in dogs. Veterinary Surgery.48(7):1318-29.
